# Pulsed Laser Synthesis of Carbon Nanostructures from Organic Molecular Liquids: Structure, Kinetics and Photophysical Properties

**DOI:** 10.1002/advs.202505883

**Published:** 2025-07-06

**Authors:** Antonio Ribeiro‐González, Carlos Agudo‐Blanco, Sergio Ramírez‐Barroso, Cristina Navío, Luis Bañares, Roger Bresolí‐Obach, Santi Nonell, Nazario Martín, David García‐Fresnadillo

**Affiliations:** ^1^ Department of Organic Chemistry Faculty of Chemical Sciences Universidad Complutense de Madrid Avenida Complutense s/n Madrid 28040 Spain; ^2^ IMDEA‐Nanoscience Campus de Cantoblanco Madrid 28049 Spain; ^3^ Department of Physical Chemistry Faculty of Chemical Sciences Universidad Complutense de Madrid Avenida Complutense s/n Madrid 28040 Spain; ^4^ Institut Químic de Sarrià Universitat Ramon Llull Via Augusta 390 Barcelona 08017 Spain

**Keywords:** carbon nanodot, carbon nanoparticle, graphene quantum dot, pulsed laser synthesis, wavelength‐dependent fluorescence

## Abstract

The striking one‐step bottom‐up synthesis of carbon nanoparticles (CNPs) from visible and near infrared (NIR) light transparent air‐equilibrated liquid aromatic compounds (benzene, toluene, chlorobenzene, aniline, pyrrole, and thiophene) under unfocused pulsed nanosecond laser irradiation (532/1064 nm) is reported. The formation of CNPs follows zero‐order kinetics with an induction period dependent on the reactivity of the organic precursor. Experimental evidence suggests a surface‐catalyzed photochemical process involving a partial change in C‐atom hybridization from sp^2^ to sp^3^, with oxygen‐based functional groups passivating the surface of the nanostructures. The presence of additional heteroatoms depends on the structure and composition of the precursor. Contingent on the starting compound, the prepared CNPs can be classified as amorphous carbon nanodots (CNDs) or layered curved‐graphene quantum dots (c‐GQDs), resembling nano‐onion fragments. CNDs are obtained from substituted benzenes or heterocyclic compounds, whereas c‐GQDs can be synthesized from benzene. Photophysical characterization of the CNPs shows both wavelength‐dependent excitation and emission bands, with constant emission quantum yields in the 1–10% range, and wavelength‐dependent emission decays displaying several lifetime components in the range 1–20 ns. Triplet exciton lifetimes longer than 5 µs and wavelength‐dependent singlet oxygen production quantum yields in the 10–40% range have been measured.

## Graphical abstract

The non‐focused nanosecond pulsed laser irradiation of aromatic liquids produces amorphous carbon nanodots (CNDs) or curved‐graphene quantum dots (c‐GQDs) by surface‐catalyzed 0‐order reactions under kinetic control. C hybridization partially changes from sp^2^ to sp^3^. O‐based groups passivate the surface. Fluorescence bands, lifetimes, and singlet oxygen sensitization by triplet excitons, are wavelength‐dependent, while emission quantum yields are wavelength‐independent.

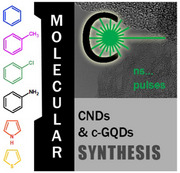

## 1 Introduction

Carbon nanoparticles (CNPs) have recently attracted great interest due to their unique structure, high specific surface area and outstanding physicochemical, optoelectronic and biological properties,^[^ ^1^ ^]^ including relatively low toxicity,^[^ ^2^ ^]^ reasonable photochemical and thermal stability,^[^ ^3^ ^]^ good dispersibility in solvents and, most importantly, functionalization or immobilization capability for the preparation of high added‐value nanomaterials (e.g., (bio)conjugates, nanocomposites or polymer‐scaffolded supported nanostructures).^[^ ^4^ ^]^ In addition, their special characteristics in terms of electronic and optical properties,^[^ ^5^ ^]^ as well as some specific features such as their size‐/excitation wavelength‐dependent fluorescence,^[^ ^6^ ^]^ make them suitable for a wide variety of applications in different fields such as bioimaging and nanomedicine,^[^ ^7^ ^]^ sensors and probes,^[^ ^8^ ^]^ energy conversion and storage,^[^ ^9^ ^]^ light emitting devices,^[^ ^10^ ^]^ microscopy,^[^ ^11^ ^]^ water treatment,^[^ ^12^ ^]^ carbon dioxide valorization,^[^ ^13^ ^]^(photo/electro)catalysis,^[^ ^14^ ^]^ information encryption,^[^ ^15^ ^]^ and as electromagnetic wave or electrochemical barriers.^[^ ^16^ ^]^

Two main preparation strategies can be used for the synthesis of carbon nanostructures (e.g., graphene quantum dots, GQDs, carbon quantum dots, CQDs, carbon nanodots, CNDs, and carbonized polymer dots, CPDs), the so‐called top‐down and bottom‐up approaches,^[^ ^17^ ^]^ and scientists search for efficient methods to synthesize and isolate highly homogeneous nanostructures, irrespective of which strategy is followed.^[^ ^18^, ^19^ ^]^ Among these methodologies, top‐down photochemical procedures, such as laser ablation of macroscopic carbon precursor targets have been extensively researched; however, bottom‐up methods starting from molecular precursors are commonly believed to be more convenient for the synthesis of nanostructures with homogeneous morphology, although the irradiation of molecular precursors in solution with laser beams has hardly been studied.^[^ ^1^, ^5^, ^20^ ^]^

Regarding laser irradiation methods to synthesize CNPs, pulsed laser ablation in liquids (PLAL) has been the most widely used top‐down process, using focused laser beams directed at macroscopic amorphous/graphitic carbon substrates or, alternatively, carbon powder suspensions and even nanomaterials such as carbon nanotubes.^[^ ^1^, ^5^, ^21^ ^]^ UV–vis/NIR laser beams with pulse durations in the femtosecond‐nanosecond range have been employed, and the role played by irradiation parameters and experimental conditions, as well as the mechanism of nanoparticle formation under PLAL have been described in detail.^[^ ^22^ ^]^ Conversely, bottom‐up approaches using pulsed laser light and small organic molecules as building blocks for the preparation of larger carbon nanostructures have been considerably less explored. These methods can be classified according to the experimental conditions used as i) laser excitation at specific wavelengths (usually UV) absorbed by the organic substrates, or ii) laser irradiation at wavelengths not absorbed by the reaction medium (i.e., organic compounds which are transparent to visible‐NIR incident light). Furthermore, focused or non‐focused laser beams may be used in the latter category, resulting in different configurations of the experimental setup and reaction medium.

In relation to those experiments where the laser light photons have a suitable wavelength to be absorbed by the organic substrates, the corresponding photochemical processes are triggered (i.e., C–X bond fission) resulting in the formation of radical species that collide and, subsequently, self‐assemble producing carbon nanostructures (e.g., photodissociation and photochemical stitching).^[^ ^23^ ^]^

Concerning those experimental setups making use of a focusing system for concentrating the laser energy in the focal volume within the solution, the extremely high power achieved, regardless of the wavelength used, produces a plasma state, causing the fragmentation of the molecular substrates in separated organic radicals, ions and electrons, whose recombination leads to the formation of carbon nanostructures. Additionally, inert inorganic substrates or microparticles can be added to the reaction medium, serving as catalysts for the production of CNPs under focused irradiation.^[^ ^22^, ^24^ ^]^ On the other hand, experiments carried out focusing the laser beam in different positions of biphasic liquid systems have also been reported.^[^ ^25^ ^]^ Finally, experiments carried out with non‐focused laser pulses and at wavelengths where the organic substrates do not absorb the incident radiation are, by far, much less common and intriguing, and only one literature reference reports on this specific method for the preparation of carbon nanoparticles.^[^ ^26^ ^]^

In this work, we systematically explore the uncharted synthesis of carbon nanostructures by non‐focused pulsed laser synthesis, their formation kinetics, structural characterization, and wavelength‐dependent photophysics, devising some of their potential applications in photosensitization by energy transfer (i.e., singlet oxygen production). The role played by the surface of the reaction container, added microparticles, light wavelength and power, and the influence of the starting organic substrates are also discussed.

## 2 Results and Discussion

### 2.1 Synthesis of Carbon Nanoparticles

A scheme of the experimental set‐up used to obtain the nanomaterials by pulsed nanosecond laser synthesis and to follow their formation kinetics is depicted in Scheme S1 (Supporting Information). CNPs were prepared from liquid organic precursors such as aromatic hydrocarbons (benzene, toluene) or aromatic compounds containing different heteroatoms (Cl, chlorobenzene; N, aniline and pyrrole; and S, thiophene) which were irradiated with a pulsed laser beam from a Nd‐YAG laser (532 or 1064 nm). Regarding the choice of these organic molecular precursors, while benzene is a highly stable molecule composed of sp^2^ carbon atoms that can be considered the reference standard for the reactivity of aromatic compounds, toluene provides a more reactive aromatic structure toward in situ generated electrophilic fragments. Additionally, toluene contains an sp^3^ carbon with highly reactive benzylic hydrogens that can enhance the potential reactivity of this CNPs precursor toward in situ generated radicals or nucleophiles, also allowing for the possibility of elimination reactions. On the other hand, chlorobenzene and aniline, which contain peripheral electron‐withdrawing or electron‐releasing functional groups, respectively, would allow testing two organic precursors of opposite reactivity. Moreover, while chlorobenzene also introduces potential reactivity with in situ generated nucleophilic fragments, in the case of weakly basic aniline, additional reactivity at the N atom could be explored. Furthermore, both reactants offer the possibility of doping the CNPs with Cl or N atoms, which may have an impact on the structural and physicochemical properties of the resulting CNPs, such as heavy atom effect allowing intersystem crossing and phosphorescence or enhanced redshifted fluorescence, respectively.^[^ ^5^, ^6^ ^]^ Finally, the choice of heterocyclic precursors such as pyrrole and thiophene, with N and S atoms in their aromatic structure, respectively, favouring reactivities similar to those of aniline or benzene, would allow testing the effect of these heteroatoms when incorporated into the CNPs. We would also like to underline that attempts to produce CNPs by our non‐focused pulsed laser synthesis method from organic precursors composed only of sp^3^ carbon atoms, such as cyclohexane or ethylene glycol, were always unsuccessful.

A laser power of 1 W (100 mJ pulse^−1^) was typically used in most experiments, although different laser fluences were tested in several experiments carried out with toluene to deeply characterize the kinetics and to improve the reaction yields as much as possible (Supporting Information). Amounts of 3 mL (quartz cuvette experiments) or up to 40 mL (reactor experiments) of the liquid organic molecular precursors (toluene, benzene, chlorobenzene, aniline, pyrrole, and thiophene) were commonly irradiated for 6‐h periods. This allowed the preparation of 1–4 mg or 10–100 mg of isolated nanomaterial for the cuvette or reactor experiments, respectively, depending on the parent molecular compound and after purification by classical laboratory procedures such as distillation, filtration, or extraction of the organic precursor (Supporting Information). The purified samples were checked by thin layer chromatography (TLC) and Fourier transform infrared (FTIR) spectroscopy to ensure the absence of molecular impurities (e.g., small molecule photoproducts) and organic precursor. An experiment with toluene in the presence of NiO microparticles was also carried out to test the influence of this medium regarding the formation of carbon nanostructures with a non‐focused nanosecond laser pulse and in order to compare with previous results using focused laser irradiation.^[^ ^23a,c^ ^]^ We would like to stress that, since this method for the preparation of carbon nanoparticles has little precedent in the literature, our main aim during the purification steps was the preparation of high‐purity nanomaterials even at the expense of isolated product yield.

### 2.2 Morphological and Structural Characterization of the Carbon Nanoparticles

**Figure 1:**
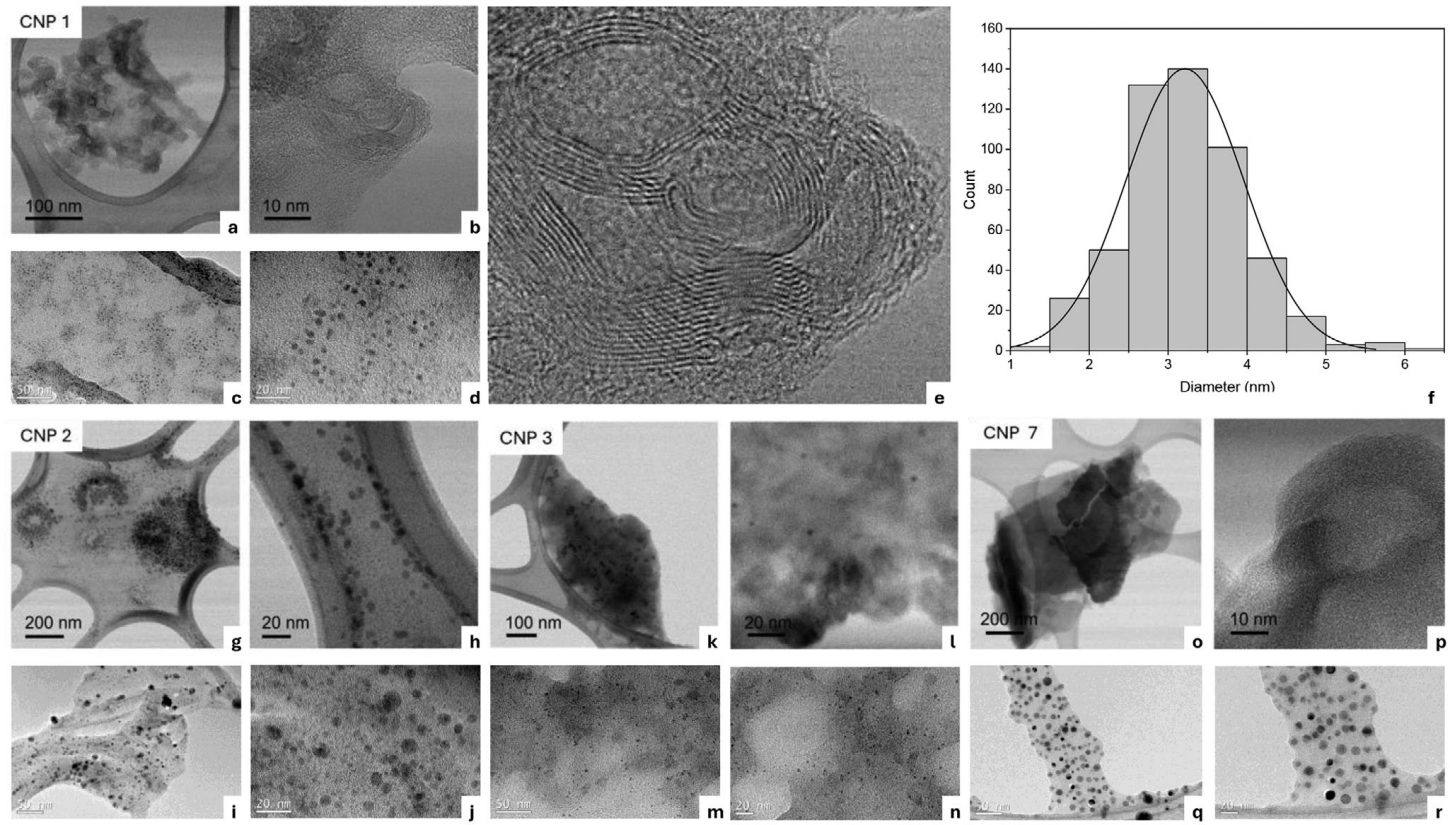
Selected high‐resolution scanning transmission electron microscopy (HR‐STEM, low magnification and high‐resolution images) and TEM images of representative samples of curved‐graphene quantum dot (c‐GQDs) and carbon nanodots (CNDs) obtained by pulsed nanosecond laser synthesis. CNP 1 from benzene: a,b) HR‐STEM, c,d) TEM, e) amplification of image (b), f) size distribution histogram. CNP 2 from toluene: g,h) HR‐STEM, i,j) TEM. CNP 3 from toluene‐NiO: k,l) HR‐STEM, m,n) TEM. CNP 7 from thiophene: o,p) HR‐STEM, q,r) TEM.

**Figures** **1** and S1 (Supporting Information) show the aspect of the individual CNPs and the remains of larger aggregates. The mean diameters of the CNPs determined from transmission electron microscopy (TEM) measurements are collected in **Table** **1**. Typical sizes of the nanostructures prepared by pulsed nanosecond laser synthesis are in the 3–11 nm mean diameter range, exhibiting quite narrow size distributions in general (Figure S1, Supporting Information).

**Table 1:** Characterization of the carbon nanoparticles from TEM, Raman spectroscopy, atomic‐force microscopy (AFM), and dynamic light scattering (DLS) measurements.

Sample	Precursor	TEM mean diameter [nm ± Std Dev]	Raman I_D_/I_G_ ^a)^	Nanomaterial type	AFM mean height [nm ± Std Dev]^b)^	DLS aggregate size [nm ± Std Dev] in 2‐propanol	DLS aggregate size [nm ± Std Dev] in toluene
CNP 1	Benzene	3.2 ± 0.7	1.02	CND / c‐GQD^c)^	2.7 ± 1.2	400 ± 100	220 ± 30
CNP 2	Toluene	6.0 ± 3.1	1.07	CND	1.5 ± 0.3	270 ± 70	160 ± 20
CNP 3	Toluene‐NiO^d)^	3.4 ± 0.6	0.99	CND	2.7 ± 1.1	100 ± 50	1.7 ± 0.3
CNP 4	Chlorobenzene	4.0 ± 0.7	1.28	CND	3.6 ± 0.7	260 ± 70	120 ± 30
CNP 5	Aniline	4.1 ± 0.8	0.97	CND	1.5 ± 0.4	200 ± 50	51 ± 5
CNP 6	Pyrrole	3.2 ± 0.8	1.14	CND	1.1 ± 0.2	530 ± 60	110 ± 10
CNP 7	Thiophene	10.8 ± 2.9	0.79	CND	1.9 ± 0.4	320 ± 60	120 ± 20

^a)^ I_D_/I_G_ is the D band to G band intensity ratio in Raman spectroscopy. D band corresponds to the disorder‐induced peak, while the G band represents the first‐order scattering from sp^2^ carbon atoms;

^b)^ Center of either Gaussian or Lorentzian fitting functions (see Table S1, Supporting Information);

^c)^ CND stands for carbon nanodot, c‐GQD stands for curved‐graphene quantum dot layers resembling nano‐onion fragments;

^d)^ Experiment with toluene‐NiO (0.255% w/w).

High‐resolution scanning transmission electron microscopy (HR‐STEM) was performed to observe the CNPs with atomic resolution. Figure 1 and Figure S2 (Supporting Information) display low magnification and high‐resolution images of the samples, showing the presence of individual nanoparticles as well as larger aggregated structures. As can be observed in the low magnification images, the nanoparticles tend to aggregate in globular structures of hundreds of nanometers. This is due to the deposition process on the carbon polymer on the Cu grids from sample dispersions in 1‐butanol or DMSO after 5 min of sonication. Amorphous‐carbon nanostructures were obtained from the precursors under laser irradiation. However, in the sample obtained from benzene (CNP 1), which is the only precursor lacking carbon sp^3^ and heteroatoms, high‐resolution images also reveal the presence of layers or disrupted carbon nano‐onion‐like fragments resembling stacked curved‐nanographene structures. The interplanar distances of the observed layers of these carbon nanostructures were measured from different images, and an average value of 3.6 Å was obtained (which is different to the typical interlayer distance of 3.34 Å in graphite). On the other hand, samples CNP 2–CNP 7, prepared from toluene, toluene‐NiO, chlorobenzene, aniline, pyrrole, and thiophene, respectively, show a marked amorphous structure. Combination of HR‐STEM with electron energy loss spectroscopy (EELS) showed that these nanomaterials were very sensitive under the electron beam, making them prone to contamination.

Interestingly, a semi‐quantitative analysis of the Csp^3^ versus Csp^2^ ratio could be performed by scanning transmission electron microscopy combined with electron energy loss spectroscopy (STEM‐EELS) with the samples containing CNP aggregates spanning the edges of the carbon polymer holes (with no carbon background). Considering that all the organic precursors contain 100% Csp^2^ (except toluene, 86%), this estimation suggested an important conversion of Csp^2^ into Csp^3^ (40% on average). These results support the aforementioned general amorphous nature of the CNPs obtained, and even the formation of curved‐nanographene structures in CNP 1, with larger than expected intershell distances. This could be due to the presence of abundant Csp^3^ defects, disrupting the formerly planar aromatic structure of the precursors when the nanomaterial is produced. A good agreement is found with the results obtained from the Raman spectra of the CNPs, where the G (≈1550 cm^−1^) and D (≈1350 cm^−1^) bands of the breathing mode of graphitic carbon and the Csp^3^ defects, respectively, show I_D_/I_G_ ratios in the 0.8–1.3 range, which indicate a relevant degree of disorder in the nanostructures under study (Table 1; Figure S3, Supporting Information). All these results show that two main types of CNPs were obtained under pulsed nanosecond laser synthesis from the organic precursors studied: i) structured curved‐graphene quantum dots (c‐GQDs) in the case of benzene, and ii) mainly amorphous CNDs, especially obtained when substituted benzene and heterocyclic precursors were used, and to some extent also from benzene (Table 1). Both types of nanostructures are compatible with the strong transformation of C hybridization from the aromatic organic precursors (basically composed of Csp^2^) to the resulting nanomaterials with important amounts of Csp^3^.

The EELS technique also allowed semi‐quantitative analysis of the C/O ratio. Some O atoms were incorporated to the mainly carbonaceous structure of the CNPs, likely from the air‐equilibrated system as well as from the SiO_2_ of the glass reactor container, which undergoes some etching in the laser beam impact zone (resulting in low amounts of organic Si that could be observed by X‐ray photoelectron spectroscopy (XPS) analysis, see below).^[^ ^27^ ^]^ Typically, the semi‐quantitative analysis found ≈10% O atoms in all the CNPs when the aggregated samples were explored by EELS. Since the organic precursors used lack the element oxygen, this result suggests a remarkable degree of O‐functionalization, as well as that the reactor bottom wall and/or the use of inorganic microparticles such as NiO could play a catalytic role in the formation of the CNPs. Moreover, this transference of oxygen atoms would contribute to the chemical functionalization of the CNPs. The low/negligible incorporation of Si/Ni in the CNPs (Ni not detected in the sample CNP 3) may be attributed to the very high melting points of the corresponding oxides (1715 and 1984 °C for SiO_2_ ‐quartz‐ and NiO, respectively, and 1648 °C for borosilicate 3.3 glass), suggesting that the temperature reached at the point of impact of the unfocused laser beam is above 1700 °C but well below 2000 °C, in good agreement with previous literature.^[^ ^28^ ^]^ Surprisingly, no significant evidence of the incorporation of other heteroatoms, such as N, S, and Cl, was observed by STEM‐EELS, considering the difficulty of the analysis due to the sensitivity of the amorphous CNPs to the electron beam, even with the instrument working at 80 kV (see above).

**Figure 2:**
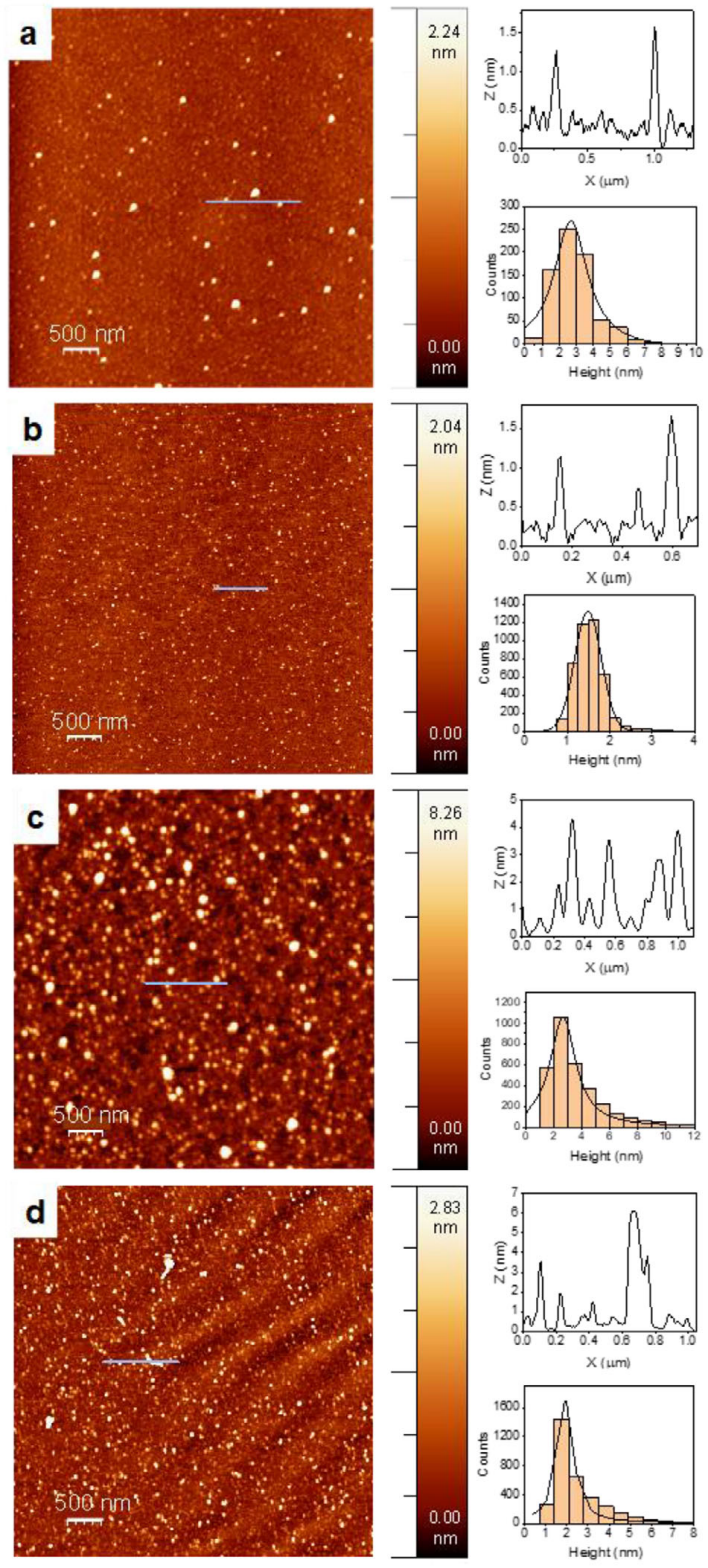
Selected AFM images, height plots, and histograms of the studied CNPs: a) CNP 1 from benzene, b) CNP 2 from toluene, c) CNP 3 from toluene‐NiO, d) CNP 7 from thiophene.

AFM experiments also made it possible to observe the CNPs individually. Average heights between 1.1 and 11.2 nm were detected for the CNPs studied, over a range of 350–6500 individual nano‐objects inspected from each different sample (Table 1; Figures S4 and S5, and Table S1, Supporting Information). In general, narrow population distributions were obtained, which explains the excellent agreement between the peak of the fitted Gaussian or Lorentzian distribution and the slightly larger median height values. The minimum heights detected were always above 0.7 nm, while the largest nano‐objects observed have heights below 40 nm (with very low relative population). **Figure** **2** shows images of selected CNPs with their size distribution histograms and Gaussian/Lorentzian fits. Considering TEM and AFM results together, nanostructures prepared by pulsed nanosecond laser synthesis show nearly spherical to discoidal shapes and, particularly in the case of precursors containing Csp^3^, N or S, disk‐shaped nanoparticles were produced from toluene, aniline, pyrrole, and thiophene.

Table 1 also shows the varying average sizes of the aggregated nanomaterials when dispersed in 2‐propanol or toluene, determined by DLS measurements, accounting for the dynamic character of the CNPs aggregation process depending on the solvent nature (polar protic vs nonpolar). DLS results in 2‐propanol show wide distributions and a mean hydrodynamic diameter of hundreds of nm (Figure S6, Supporting Information), revealing the strong tendency to form big particle aggregates, with much larger sizes compared to the individual nanoparticles, in a protic and relatively polar solvent. On the other hand, when CNPs were dispersed in hydrophobic toluene, they displayed much narrower size distributions (Figure S6, Supporting Information). However, although the hydrodynamic diameter decreases in toluene with respect to 2‐propanol suspensions by 2–5 times, it is still high compared to individual nanoparticles, an exception being sample CNP 3, prepared from toluene‐NiO, where an average hydrodynamic diameter of 1.7 nm was found in toluene (Table 1), in good agreement with TEM and AFM results. Overall, these results show that CNPs in toluene also form aggregates but much smaller and with narrower distributions than in 2‐propanol. Time‐dependent DLS measurements also evidenced that these nanomaterials tend to dynamically form larger aggregates and sediment over time.

FTIR spectra confirmed the partial Csp^2^ to Csp^3^ transformation, besides the presence of functional groups with oxygen on the surface of the CNPs (Figure S7, Supporting Information). Some common features generally detected in the IR spectra of the CNPs include: i) the ≈2957, 2924 and 2854 cm^−1^ highly characteristic multiplet due to Csp^3^–H stretching vibrations, accounting for the partial Csp^2^ to Csp^3^ transformation (also related to the presence of ether‐like adjacent O atoms, in the case of the 2854 cm^−1^ peak); ii) the 3500–3200 cm^−1^ O–H stretching (H‐bonded OH, broad, with no detectable presence of free OH sharp peaks ≈3600 cm^−1^), likely due to OH interactions between the aggregated CNPs in the sample; iii) 3050–3020 cm^−1^ Csp^2^–H stretching; iv) 1750–1660 cm^−1^ (H/R–)O–C═O, O–(C═O)–O, C═O and C═C–C═O stretching vibrations; v) 1630–1590 cm^−1^ due to C═C and C═C–O stretching; vi) 1470–1360 cm^−1^ including Csp^3^–H and Csp^2^–H bending, overlapped with O–H bending; vii) 1280–1020 cm^−1^ in the ether‐like interval of functional groups, due to Csp^3^–O–Csp,^3^ Csp^2^–O–Csp^3^ and Csp^2^–O–Csp^2^ stretching vibrations; viii) 950–700 cm^−1^ in the Csp^2^–H out of plane deformation region; and for the samples prepared from aniline and pyrrole (CNP‐5 and CNP‐6, respectively) ix) the H‐bonded N─H stretching bands superimposed with the O─H stretching but shifting the peak of the band to lower values. No evidence of S─H or S═O stretching vibrations was found in the case of CNP‐7, however, signals of O─H, H─O─C═O, and C═C─C═O stretching vibrations were detected in this sample.^[^ ^29^ ^]^

**Figure 3:**
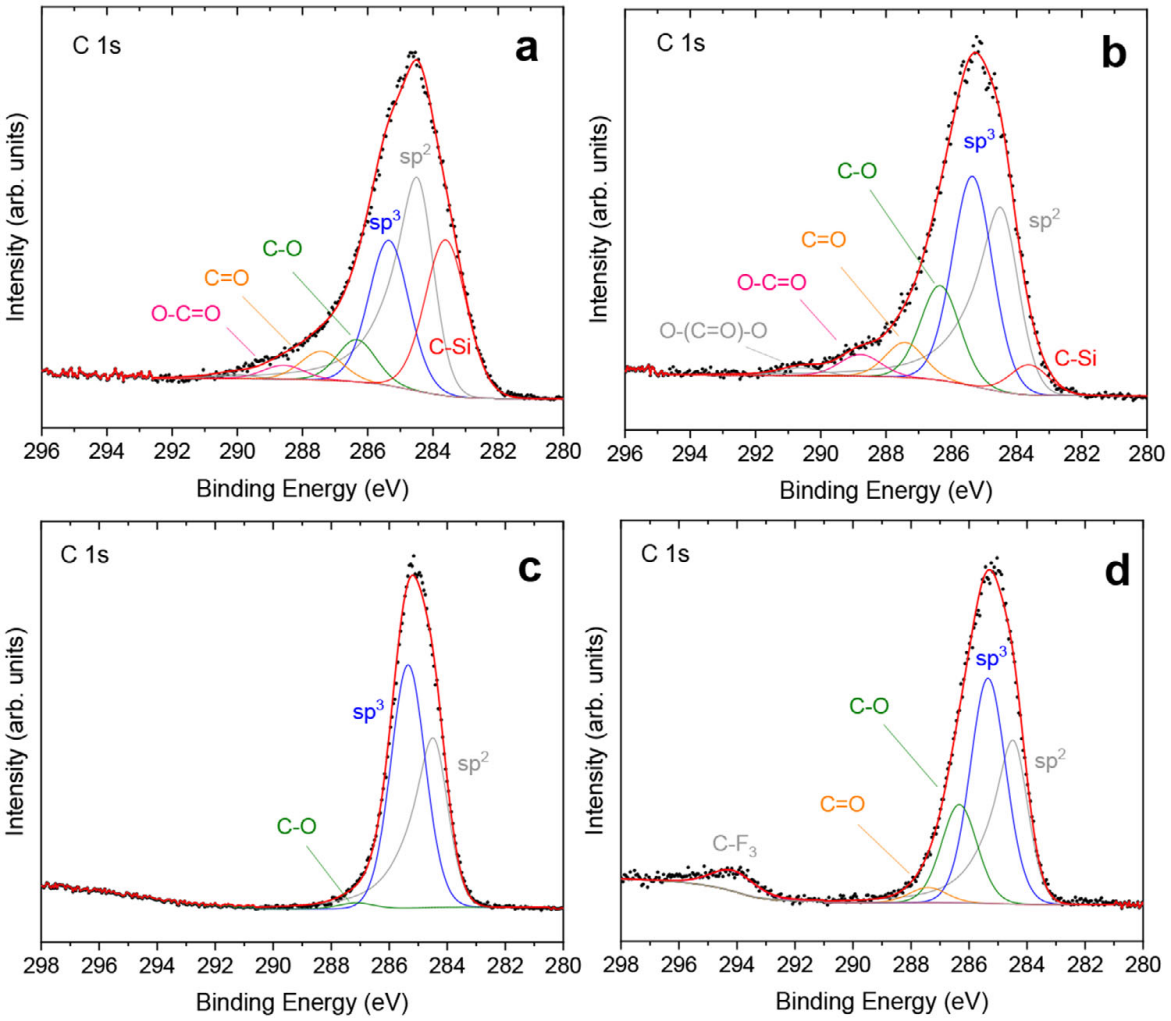
Selected XPS spectra of the C 1s core of the studied CNPs: a) CNP 1 from benzene, b) CNP 2 from toluene, c) CNP 3 from toluene‐NiO, d) CNP 7 from thiophene.

XPS measurements allowed us to determine the elemental composition and the different types of C bonds present in all samples. **Figures** **3** and S8 (Supporting Information) show the C 1s core level of each sample. In all cases, the sp^2^ component centered at 284.5 eV has been used as the binding energy reference and has the typical asymmetric shape of the graphitic Csp^2^. The rest of the components are a combination of Gaussian‐Lorentzian curves centered in the following positions: Csp^3^ at 285.4 eV, C─O at 286.4 eV, C═O at 287.4 eV, O─C═O at 288.9 eV (with small differences depending on the sample) and a low binding energy component at 283.6 eV attributed to C─Si due to the incorporation of Si from the ablated reactor walls into the CNPs.^[^ ^30^ ^]^ For samples obtained from precursors containing N (Figure S9, Supporting Information), a N 1s peak has been detected as well, and the corresponding component in the C 1s peaks overlapped with other C‐O moieties. The N 1s core level of sample CNP 6 from pyrrole has one component centered at 400.1 eV that could be assigned to pyrrolic N.^[^ ^31^ ^]^ On the other hand, in the sample CNP 5 from aniline, the N peak is too broad to be considered as a single component, so two components have been taken into account, centered at 399.8 and 401.2 eV, that can be assigned to pyridinic N and graphitic N, respectively.^[^ ^32^ ^]^ Little presence of Cl was detected in the sample prepared from chlorobenzene (CNP 4) and, in excellent agreement with FTIR results, no presence of S was detected in the sample prepared from thiophene (CNP 7). Tables S2 and S3 (Supporting Information) show, respectively, the atomic percentage of all the relevant elements detected in each sample, and the % contributions of the main functional groups detected in the XPS analysis of the CNPs studied, showing good general agreement with the FTIR results. Interestingly, the highest relative Csp^2^/Csp^3^ ratio (>1) has been detected in the sample prepared from benzene (Figure 3a; Figure S8 and Table S3, Supporting Information), in good agreement with the observation of curved‐graphene quantum dot structures in this sample (Figure 1e). Conversely, the rest of the samples (from precursors containing either a Csp^3^ or one heteroatom per molecule) always showed Csp^2^/Csp^3^ ratios <1, according to their basically amorphous nature. Moreover, the sample prepared from toluene‐NiO exhibits the lowest percentage of oxygen atoms in its composition (Table S2, Supporting Information). This finding suggests that the presence of oxygen in the CNPs is attributable to the decomposition of the SiO_2_ surface (a process that does not occur in the toluene‐NiO experiment, where laser light is incident on NiO microparticles that have a higher melting point) and not to molecular oxygen dissolved in the air‐equilibrated organic precursor. Furthermore, results in Table S3 (Supporting Information) show that sample CNP 3 contains the highest % of Csp^3^. Taken together, the high presence of Csp^3^ but low oxygen content in the sample prepared from toluene‐NiO is probably the cause of poor surface functionalization and high surface hydrophobicity, and this could be the reason for its negligible self‐aggregation in the nonpolar solvent toluene, as shown by the DLS results (Table 1; Figure S6, Supporting Information).

Thermogravimetric analysis (TGA) was carried out under a nitrogen atmosphere for all the CNPs samples. TGA plots of the CNPs are shown in Figure S10 (Supporting Information). A strong weight loss is typically observed for almost all the CNPs, in the 100–600 °C interval, associated to a 45–61% decrease: 45% for CNP 6 from pyrrole, whose CNPs retain a significant amount of N (≈12%, by XPS); while 58% for CNP 1 (CNDs/c‐GQDs from benzene) and 61% for CNP 7 from thiophene (where no S was detected in the pristine CNPs). Conversely, two samples exhibit much better thermal stability, CNP 3 (from the singular toluene‐NiO experiment) displaying only two small weight losses in the intervals of 100–500 °C (16%) and 500–600 °C (11%), and CNP 5 from aniline, with some graphitic N, showing only 11% weight loss. Generally, losses up to 600 °C in the CNPs can be attributed to the presence of oxygenated functional groups that are successively detached from the carbon nanostructure, such as inner alcohol, ether/epoxy, carboxylic acid, carbonyl, or outer/edge alcohol that are gradually removed from the CNPs.^[^ ^33^ ^]^ Therefore, the most labile functional groups, attached to Csp^3^ fragments, would be lost at lower temperatures, while the less labile functional groups would be removed at higher temperatures, close to 500–600 °C. The Csp^2^ graphitic domains existing in the 600–800 °C range seem to be rather stable in all the CNPs and only at temperatures above 800 °C these domains start their decomposition.^[^ ^34^ ^]^ Overall, samples CNP 3 (from toluene‐NiO) and CNP 5 (from aniline) seem to be particularly thermally stable, followed by the sample prepared from pyrrole (CNP 6), highlighting the role played by N heteroatoms; while samples CNP 1 (with stacked c‐GQDs, prepared from benzene) and CNP 7 (from thiophene) are more prone to thermal decomposition. The higher lability of CNPs 1 and 7 is probably due to inherent structural differences in CNP 1 (facilitating thermal decomposition in the case of the defect‐rich c‐GQDs resembling nano‐onion fragments), or to a higher content of structural defects in the amorphous CNDs prepared from thiophene, caused by efficient loss of S oxides during CNP 7 formation, respectively.

### 2.3 Kinetics of Carbon Nanoparticles Formation

Regarding the kinetics of CNPs generation, CNPs formation was monitored by following the onset of sample fluorescence and the increase in emission during laser irradiation, assuming that the observed fluorescence in the UV–vis region increases with the population or concentration of excited states of the CNPs, and that no self‐quenching effect occurs due to the high dilution of CNPs in the reaction crude. The visible excitation and detection wavelengths generally employed for the monitorization of CNPs formation were specifically chosen to avoid interference with the UV absorption and fluorescence of precursors and from potential UV‐absorbing impurities. Also note that while the possible fluorescent molecular impurities should show a constant emission band, the synthesized CNPs (either c‐GQDs or CNDs) show excitation‐wavelength dependent emission, as will be discussed in the next section.

The effect of the irradiation wavelength was first tested with experiments performed in quartz cells. Toluene was used as the molecular organic precursor irradiated with nanosecond laser pulses of 2.3 W cm^−2^ irradiance at either 1064 or 532 nm. Figure S11 (Supporting Information) shows the linear increase of the fluorescence intensity detected at 510 nm upon excitation at 443 nm. A much steeper slope (≈30 times) was measured when the experiment was carried out with 532 nm irradiation, which is evidence that higher energy per photon clearly enhances the formation of CNPs.

Next, the effect of flux density was also tested with toluene as the carbon source. Figure S12 (Supporting Information) shows that the effect on the linear increase of CNPs fluorescence by irradiation of the sample with 2.3 or 3.5 W cm^−2^ is ≈2‐fold only. Formation of CNPs did not occur at flux densities ≈1.8 W cm^−2^ or below. Interestingly, slower kinetics were observed with both 1064 and 532 nm irradiation if a continuous monitorization of CNPs formation via sample fluorescence was performed (i.e., with continuous excitation at 443 nm during the synthesis experiments) compared to excitation only every 5 min for the acquisition of the corresponding fluorescence spectrum. This suggests that the formation of CNPs after the laser pulse, once the photochemical breakdown of the molecular precursors has been triggered, is a dark thermally‐driven process, since continuous excitation at 443 nm appears to divert the reaction intermediates from CNPs generation (Figure S13, Supporting Information). This interesting result is consistent with the abovementioned etching observed in the laser beam impact zone of the reactor glass vessel (≈90% light transmission at the 1064 or 532 nm irradiation wavelengths used), suggesting that hot spots formed on the irradiated glass surface, above a certain flux density threshold (≈2 W cm^−2^), promote the formation of CNPs under kinetic control conditions. That is, the radicals and radical ions generated after the short and intense laser pulse react rapidly and form covalent bonds following pathways with the lowest possible activation energy. This fact also justifies the partial conversion of Csp^2^ to Csp^3^ and the abundant formation of C–C bonds (with less steric and energy requirements over C═C bonds) and the predominant amorphous nature of the nanostructures obtained by pulsed laser synthesis. Furthermore, the use of a precursor without any substituent or functional group, such as benzene, can also explain the additional formation of c‐GQDs with higher regularity and relative Csp^2^ content.

**Figure 4:**
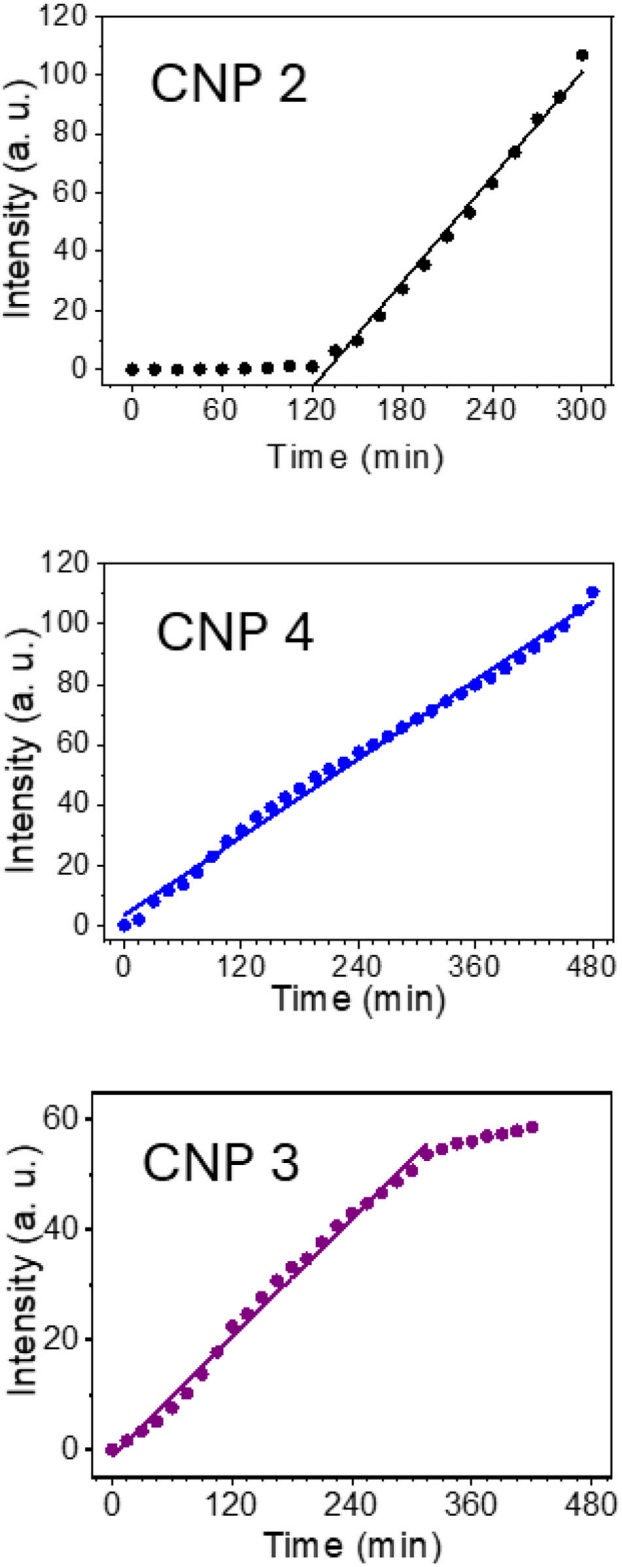
Time‐dependent fluorescence plots (*λ*_exc_ = 400 nm, *λ*_det_ = 510 nm), representing the three kinetic behaviors found in the pulsed laser synthesis of CNPs: i) CNP 2 from toluene, with an induction period; ii) CNP 4 from chlorobenzene, without induction period; and iii) CNP 3 from toluene‐NiO, without induction period and with saturation plateau.

**Figures** **4** and S14 (Supporting Information) show the fluorescence intensity versus time plots of experiments carried out in the photochemical reactor (see Supporting Information), displaying the different behaviors found.

Three types of kinetics have been observed:

- Samples in which an induction period of 60–150 min is required before the formation of fluorescent CNPs is detected. This behavior is probably associated with the formation and slow growth of nonfluorescent reaction intermediates (small molecular fragments with radical, radical ion, and/or ionic nature). Following the induction period, a linear increase in the concentration of fluorescent CNPs was observed. Examples of this behavior are the CNPs prepared from benzene and toluene, both with induction times ≈150 and 120 min, respectively; or from aniline and pyrrole, with induction times ≈1 h. Interestingly, the induction time increases in this order: aniline ≈ pyrrole < toluene < benzene; following a trend opposite to the increasing electron‐releasing effect due to the presence of the substituent (–CH_3_, –NH_2_) or heteroatom (pyrrolic N), suggesting that an increased electron density on the aromatic ring relative to benzene decreases the induction period. However, it has to be mentioned that the slopes (*s*) of the kinetic profiles are higher in the case of benzene (*s* = 0.50) and toluene (*s* = 0.60) precursors compared to aniline (*s* = 0.05) and pyrrole (*s* = 0.05), suggesting that the formation of stable fluorescent CNPs, once enough nonfluorescent reaction intermediates have been formed, proceeds faster in precursors without N atoms (i.e., the presence of N atoms slows down the formation of fluorescent CNPs). Therefore, N‐containing precursors, although more reactive than C‐based ones, are likely to produce intermediates that divert the reaction pathways toward the formation of molecular or ionic species that do not lead to the formation of fluorescent nanoparticles, especially if the N atom belongs to a peripheral functional group (aniline), whereas if it belongs to the heteroaromatic ring (pyrrole) there is more chance of nitrogen incorporation into CNPs (see XPS discussion above, and Table S2, Supporting Information).
- Samples with reaction kinetics in which no induction period is necessary for the generation of CNPs, as in the CNPs prepared from chlorobenzene and thiophene precursors. These heteroatom‐containing precursors have less activated aromatic rings than aniline or pyrrole, however, they comprise labile C–Cl bonds (Cl being a good leaving group) or C–S bonds (being thiophene a molecule with less aromaticity than benzene). Furthermore, incorporation of S atoms into the CNPs after molecular breakdown was not detected (see discussion of FTIR and XPS results above), probably due to the formation of volatile sulfur oxides or their nonvolatile salts, which are removed during processing of the reaction products. Therefore, formation of reactive intermediates occurs faster in the case of chlorobenzene and thiophene compared to aniline, pyrrole, toluene, and benzene; suggesting that the presence of heteroatoms, particularly if involved in labile covalent bonds, increases the general reactivity of the corresponding precursors under our experimental conditions. However, despite the induction period required by the aromatic hydrocarbons, the subsequent growth of the reaction intermediates to form detectable fluorescent CNPs occurs much more rapidly with benzene (*s* = 0.50) and toluene (*s* = 0.60) compared to the heteroatom‐containing precursors, chlorobenzene (*s* = 0.23) and thiophene (*s* = 0.08), reflecting that slowlier kinetics of CNP formation are observed as the electron withdrawing character decreases (chlorobenzene > thiophene > aniline ≈ pyrrole). This fact can be explained by taking into account that the incorporation of N, Cl or S into CNPs is low or negligible (S) and that the extruded heteroatoms of the broken molecular precursor do not generally participate in the formation of reactive intermediates that are finally incorporated into the resulting CNPs but are, instead, involved in the formation of non‐fluorescent impurities that are eventually removed during the purification processes.
- The toluene‐NiO sample, where higher reaction temperatures at the laser impact zone are achieved due to light absorption by the green NiO microparticles. In this case, a linear increase of CNPs production with no induction period can be observed (as opposed to only toluene). This regime is followed by a plateau that can be attributed to saturation or depletion of the active sites creating hot spots on the NiO surface, in good agreement with the color change from green to gray observed in the NiO microparticles throughout the experiments. Again, although no induction period is required, slower kinetics of fluorescent CNPs formation is observed in this case (*s* = 0.18, sligthly lower than that of the experiment with chlorobenzene). This behavior can be connected to the highest % Csp^3^ and lowest content of O atoms detected in these CNPs, compared to the rest of samples (XPS results in Tables S2 and S3, Supporting Information), due to the high melting point of green NiO enabling the attainment of elevated temperatures within the laser impact zone without the release of Ni or O atoms.

From the discussion above on the influence of precursors on the formation kinetics and characteristics of these CNPs, remarkably intricate behavior is revealed. The formation of CNPs under our relatively mild method, based on non‐focused pulsed laser irradiation of different organic precursors, is therefore dependent on several structural and reactivity aspects that occur simultaneously. These aspects include molecular aromaticity, the nature of the functional groups on the precursor, and their ability to release or withdraw electrons from the aromatic ring. In addition, the existence of heteroatoms associated with labile covalent bonds must be considered. In summary, it has been shown that the presence of heteroatoms forming labile covalent bonds enhances the formation of reactive species, while concomitantly reducing the reaction kinetics leading to the production of fluorescent CNPs.

Finally, as far as the functionalization of CNPs is concerned, the most important contribution is related to O‐containing functional groups, which are not derived from the organic precursor but rather from the reaction vessel made of SiO_2_ (see discussion above on EELS, FTIR, and XPS results). On the other hand, irrespective of the necessity for an induction period in certain precursors, the previously mentioned observation that heteroatoms impede the formation kinetics of CNPs may be the ultimate rationale for the generally low incorporation of heteroatoms, such as N, Cl, or S into CNPs. The only exception to this phenomenon is found in pyrrole, where the nitrogen atom is part of a highly stable aromatic ring in this precursor.

Summarizing, during the formation of CNPs by pulsed laser irradiation, linear time‐dependent increases in fluorescence intensity were observed, i.e., constant reaction rates in all cases (Figure 4; Figures S11–S14, Supporting Information). This fact would point to zero‐order kinetics governing the pulsed nanosecond laser synthesis of the nanostructures. Zero‐order kinetics exhibit constant reaction rates, which are independent of the reactant concentration provided that any reactant is always present. Since the concentration of organic precursors is in the range 9.3–14.4 m in these experiments, and laser radiation with homogeneous fluence is continuously supplied, the above assumptions can be fulfilled. Moreover, zero‐order rate laws are typical of heterogeneous reactions and surface‐catalyzed processes. Therefore, it is plausible that the irradiated surface of the reaction vessel or the solid microparticles added to the reactor (in the case of the experiments performed in the presence of NiO) can play a catalytic role, photochemically generating hot spots that promote the formation of intermediates that subsequently evolve following a reaction mechanism under kinetically controlled conditions. The growth of these reaction intermediates may require an induction time but eventually leads to the formation of nanoparticles, mainly of the CND type, especially in the case of precursors bearing sp^3^ hybridized carbon or heteroatoms, since the kinetic control tends to favor fast‐forming but less stable products, i.e., defect‐rich nanoparticles. In the case of the Csp^2^‐based benzene precursor, in the absence of heteroatoms that would hinder reaction kinetics, the formation of c‐GQDs is also possible.

### 2.4 Photophysics of Carbon Nanoparticles Prepared by Pulsed Nanosecond Laser Synthesis

**Figure 5:**
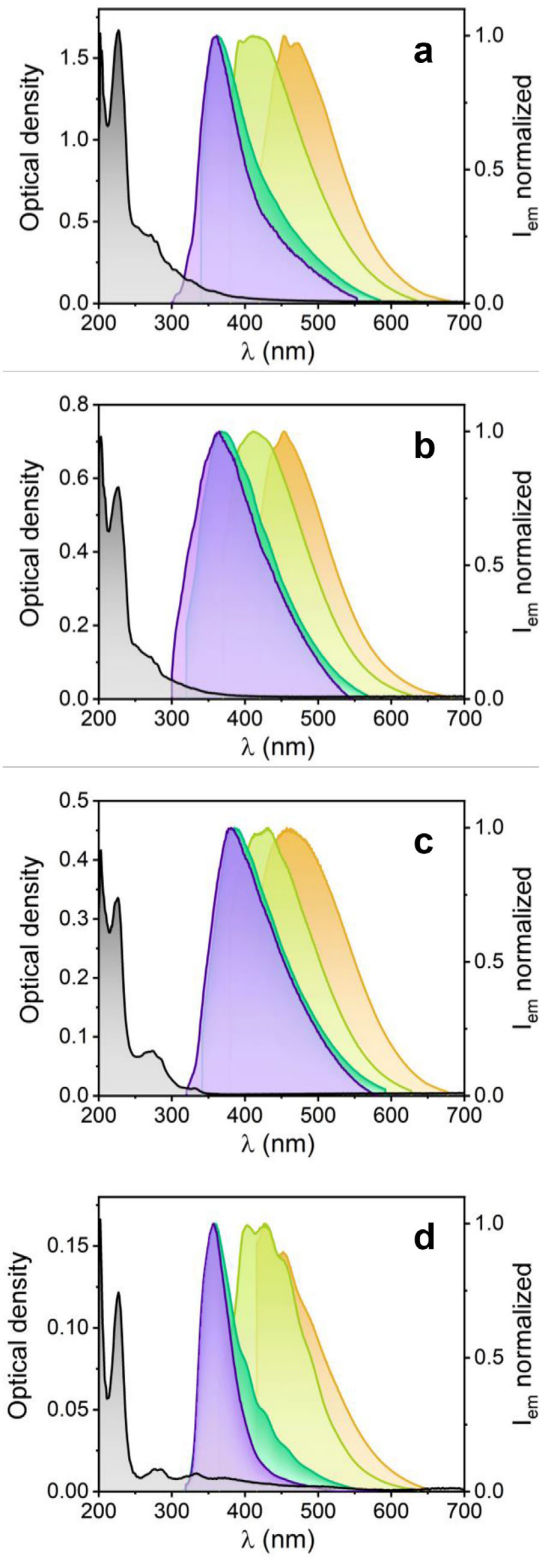
UV–vis absorption (gray) and normalized emission spectra of selected CNPs in 2‐propanol. Absorption spectra have been corrected for Rayleigh scattering. a) CNP 1 from benzene (2.90 µg mL^−1^), b) CNP 2 from toluene (3.33 µg mL^−1^), c) CNP 3 from toluene‐NiO (0.42 µg mL^−1^), d) CNP 7 from thiophene (66.7 µg mL^−1^). Emission spectra acquired with absorbance ≤0.1 at *λ*_exc_ (280 nm (purple), 300 nm (blue), 350 nm (yellow), and 400 nm (orange)).

**Figure** **5**; Figure S15–S17 (Supporting Information) show the absorption, excitation, and emission spectra of the CNPs in 2‐propanol, a protic and relatively polar solvent suitable for interacting with the surface polar functional groups as well as the hydrocarbon structure of CNPs. The UV–vis absorption spectra have been corrected for Rayleigh scattering,^[^ ^35^, ^36^ ^]^ due to the observed strong tendency for nanoparticle aggregation. All the absorption spectra show an intense band with two or more peaks between 220–280 nm (Table S4, Supporting Information), and one or more peaks/shoulders in the UV–vis region above 300 nm, with a tail extending the absorption of light beyond 400 nm. The samples CNP 1–3, prepared from benzene, toluene, and toluene‐NiO exhibit less pronounced visible absorption compared to the samples CNP 4–7 prepared from heteroatom‐containing precursors (chlorobenzene, aniline, pyrrole, and thiophene, respectively).

Figure S16 (Supporting Information) shows the UV–vis excitation spectra of the CNPs, which are dependent on the emission detection wavelength, and display excitation bands with several peaks/shoulders in the 260–300, 300–340, and 360–400 nm intervals, depending on the detection wavelength. Similarly, Figure S17 (Supporting Information) displays the UV–vis emission spectra of the CNPs, with absorbance <0.1 at the corresponding excitation wavelength to avoid primary and secondary inner filter effect artifacts causing diminished fluorescence and distortions in the high‐energy flank of the measured emission bands. All the emission spectra recorded show excitation‐wavelength dependent fluorescence broad bands, as usually observed in many carbon nanoparticles reported in the literature, commonly associated to the distribution of nanoparticle sizes in the sample, although other justifications such as those involving the presence of diverse fluorophoric groups with different number of electrons involved, emission from the surface/core of the nanoparticle or clusterization‐triggered emission should not be discarded.^[^ ^37^ ^]^ Interestingly, CNPs 3, 5 and 6, prepared from toluene‐NiO, aniline and pyrrole, respectively, which show smaller weight losses in the TGA analysis (suggesting more stable structures), tend to show redshifted emission bands (>10 nm; Table S5, Supporting Information) compared to CNPs prepared from precursors with no heteroatom, like benzene or toluene (CNPs 1 and 2) or samples from chlorobenzene and thiophene (CNPs 4 and 7). The latter showed slightly blueshifted fluorescence bands in fact. No significant effect of the excitation wavelength was observed concerning the FWHM values of the emission bands. The above observations suggest that fine‐tuning of the fluorescent properties of these CNPs can be achieved by selecting a suitable organic precursor.

**Table** **2** collects the fluorescence emission quantum yields (*Φ*_em_) of the CNPs in 2‐propanol, determined at different excitation wavelengths. The values of *Φ*_em_ were independent of the excitation wavelength within experimental error. Remarkably, the CNPs prepared from benzene and toluene displayed higher *Φ*_em_ compared to those prepared in the toluene‐NiO experiment or from heteroatom‐containing precursors. Interestingly, in CNPs prepared by hydrothermal methods, the presence of heteroatoms usually has an opposite effect, leading to higher *Φ*_em_ values.^[^ ^38^ ^]^

**Table 2:** Fluorescence quantum yields, amplitude‐weighted average emission lifetimes, and radiative and nonradiative deactivation rate constants of the CNPs in 2‐propanol, determined at different excitation wavelengths.

Sample^a)^	*Φ*_em_	*τ*_0AMP_ [ns]	*k*_r_ [s^−1^]	*k*_nr_ [s^−1^]
CNP 1	0.09 ± 0.01	2.3 ± 0.2	3.9 10^7^	4.0 10^8^
CNP 2	0.09 ± 0.01	2.1 ± 0.3	4.3 10^7^	4.3 10^8^
CNP 3	0.05 ± 0.01	1.9 ± 0.5	2.6 10^7^	5.0 10^8^
CNP 4	0.03 ± 0.01	1.6 ± 0.7	1.9 10^7^	6.1 10^8^
CNP 5	0.03 ± 0.01	2.0 ± 0.1	1.5 10^7^	4.9 10^8^
Sample^b)^	*Φ*_em_	*τ*_0AMP_ [ns]	*k*_r_ [s^−1^]	*k*_nr_ [s^−1^]
CNP 6	0.01 ± 0.003^c)^	3.3 ± 0.2	3.0 10^6^	3.0 10^8^
CNP 7	0.03 ± 0.01^d)^	2.4 ± 0.2	1.3 10^7^	4.0 10^8^
Sample^e)^	*Φ*_em_	*τ*_0AMP_ [ns]	*k*_r_ [s^−1^]	*k*_nr_ [s^−1^]
CNP 1	0.08 ± 0.01	2.8 ± 0.7	2.9 10^7^	3.3 10^8^
CNP 2	0.08 ± 0.01	2.5 ± 0.6	3.2 10^7^	3.7 10^8^
CNP 3	0.04 ± 0.01	1.9 ± 0.4	2.1 10^7^	5.1 10^8^
CNP 4	0.02 ± 0.01	2.6 ± 0.1	7.7 10^6^	3.8 10^8^
CNP 5	0.04 ± 0.01	2.0 ± 0.4	2.0 10^7^	4.8 10^8^

^a)^ *λ*_exc_ 355 nm, 0.01–0.1 mg mL^−1^ concentration range, corresponding to absorbance ≤0.1 at *λ*_exc_;

^b)^ *λ*_exc_ 405 nm, absorbance ≤0.1 at *λ*_exc_;

^c)^ *Φ*_em_ = 0.01 was also determined and doubly checked at *λ*_exc_ 375 nm using quinine sulfate (QS) as the fluorescence quantum yield standard (*Φ*_em QS_ = 0.55 ± 0.05, in H_2_SO_4_ 0.5 M);

^d)^ *Φ*_em_ = 0.03 was also determined and doubly checked at *λ*_exc_ 375 nm using QS and 300 nm using 2,5‐diphenyloxazole (PPO) as the fluorescence quantum yield standard (*Φ*_em PPO_ = 0.842 ± 0.042, in cyclohexane);

^e)^ *λ*_exc_ 457 nm, 0.02–0.4 mg mL^−1^ concentration range, corresponding to absorbance ≤0.1 at *λ*_exc_.

Table S6 (Supporting Information) collects the fluorescence emission lifetimes (*τ*) of the CNPs in 2‐propanol under air‐equilibrated conditions and at several excitation wavelengths. Accurate fitting of the emission decays usually required triexponential functions (except for *λ*_exc_ = 502 nm, where a biexponential fit was of sufficient quality). Interestingly, the lifetime of the longest‐lived component (*τ*_1_), typically above 8 ns, was strongly dependent on the excitation wavelength, following the energy‐gap law, while the lifetimes of the shorter‐lived components (*τ*_2_ and *τ*_3_) remained constant, independently of the excitation wavelength, within the experimental uncertainty (8–22% for *τ*_2_, and 13–26% for *τ*_3_, due to excitation light scattering strongly affecting the shortest‐lived component in the latter case). The amplitude‐averaged fluorescence lifetime of each CNP (*τ*_AMP_) was essentially independent of the excitation wavelength, while the intensity‐weighted average lifetime (*τ*_INT_) typically decreased upon increasing the excitation wavelengths due to the larger contribution of the longer‐lived component. As a final observation, the emission lifetimes under Ar‐purged conditions were longer for CNP 1 and CNP 2 samples compared to CNPs 3–7, again highlighting the role played by the presence of the NiO catalyst in the reaction crude or by heteroatoms in the organic precursor (Table S7, Supporting Information).

From the values of *Φ*_em_ and *τ*_0AMP_ (amplitude‐weighted average lifetime under Ar‐purged conditions) collected in Table 2, the radiative (*k*_r_) and nonradiative (*k*_nr_) deactivation rate constants were estimated.^[^ ^39^, ^40^ ^]^ *k*_nr_ was about one order of magnitude higher than *k*_r_, particularly for the CNPs obtained from heteroatom‐containing precursors, and both *k*_r_ and *k*_nr_ values were slightly dependent on the excitation wavelength, reflecting its influence on the longest lifetime *τ*_1_ (Table S7, Supporting Information), as well as justifying the invariance of *Φ*_em_ with the excitation wavelength despite the observed changes in the emission lifetimes.

At this point, a very important correlation arises between the reaction kinetics, the structure of the CNPs and their photophysical properties, since the kinetically controlled conditions prevailing during the synthesis of CNPs cause the formation of defect‐rich nanoparticles (mainly CNDs, but also c‐GQDs in the sample prepared from benzene, see Table 1 and Figure 1). The samples with the higher *Φ*_em_ values (≈0.09), CNP 1 and CNP 2 prepared from benzene and toluene, respectively, are also the samples with the higher relative Csp^2^/Csp^3^ ratios (1.22, from benzene) and (0.79, from toluene), compared to the rest of the samples, CNPs 3–7, synthesized from precursors bearing heteroatoms or from the toluene‐NiO experiment, for which *Φ*_em_ is always <0.05 and Csp^2^/Csp^3^ ratios are always <0.60, i.e., for structures richer in defects (Table 2; Table S3, Supporting Information).

Moreover, the reason for the aforementioned correlation can be established when the *k*_r_ and *k*_nr_ values of the different CNPs are compared under identical experimental conditions (because these deactivation rate constants are slightly dependent on the excitation wavelength, and results in Table 2 can only be compared for CNPs 1–5 with *λ*_exc_ 355 nm (first five entries) or *λ*_exc_ 457 nm (last five entries)). In this way, it can be clearly appreciated that samples CNP 1 and CNP 2 (with higher Csp^2^/Csp^3^ ratios and, therefore, a lower degree of structural defects) always show higher *k*_r_ but lower *k*_nr_ values when compared with the corresponding *k*_r_ or *k*_nr_ values of the samples prepared from heteroatom‐containing precursors or in the presence of NiO additive (with lower Csp^2^/Csp^3^ ratios and, therefore, a higher degree of structural defects, see Table S3 (Supporting Information) and Table 2, independently of the excitation wavelength considered).

Furthermore, if a correlation between *k*_nr_ and the presence of structural defects in the CNPs is assumed, the fact that the *k*_nr_ of CNP 2 is larger than that of CNP 1 (irrespective of the excitation wavelength considered, Table 2), also shows excellent agreement with the CND nature (i.e., defect‐rich) of CNP 2 nanoparticles (with Csp^2^/Csp^3^ = 0.79) compared to CNP 1 nanoparticles, with the highest Csp^2^/Csp^3^ ratio (1.22), suggesting the important contribution of more structured c‐GQDs in the CNP 1 sample.

Regarding oxygen effects on the CNPs fluorescence, Table S7 (Supporting Information) collects the fluorescence emission decay lifetimes (*τ*_i_, *i* = 1–3, for the discrete components; and *τ*_INT_ and*τ*_AMP_, for the mean lifetimes) of the CNPs in 2‐propanol under Ar‐purged, air‐equilibrated and O_2_‐purged conditions, with 375, 405 or 457 nm excitation wavelengths for the different samples. Interestingly, the longest‐lived decay component, *τ*_1_, and the intensity‐weighted average lifetime, *τ*_INT_, were highly dependent on the O_2_ concentration in solution as well as the excitation wavelength (as mentioned above), while *τ*_AMP_ was found to be insensitive and kept constant within experimental uncertainty.

Analysis of the corresponding Stern‐Volmer plots from steady‐state and time‐resolved experiments allowed the calculation of the dynamic bimolecular deactivation rate constants for the quenching of the singlet exciton of the CNPs by molecular oxygen in 2‐propanol (Figure S18 and Table S8, Supporting Information). The values of the quenching rate constants were estimated at several excitation wavelengths from *τ*_INT_ data (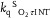) and from emission intensity measurements (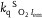).^[^ ^40^ ^]^ Good linear fits (commonly with *R* ^2^ >0.993) were obtained with 375 and 405 nm excitation. The rate constants obtained by the two methods were similar, despite the typically large experimental error inherent to the multiexponential decays characteristic of nanoheterogeneous systems.^[^ ^41^ ^]^ Attempts to use the O_2_‐sensitive *τ*_1_ values to determine 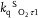 and correlate them with 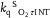 or 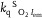 were unsuccessful due to the previously mentioned reasons. This suggests that the discrete lifetime components cannot be interpreted separately and lack physicochemical meaning, indicating high heterogeneity among the emissive centers. Furthermore, the 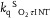 and 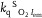 values are close to the 10^10^ m ^−1^s^−1^ diffusion‐controlled limit,^[^ ^42^ ^]^ suggesting rapid quenching of the singlet excitons of the CNPs by molecular oxygen despite their short emission lifetime, also probably linked to the dynamic nature of the CNP aggregates dispersed in solution (see above). The probability of singlet exciton quenching by O_2_ (*P*o_2_^S^) was estimated for a given % O_2_, from both the fluorescence intensity and lifetime values.^[^ ^43^ ^]^ Under air‐equilibrated conditions *P*o_2_^S^ *_τ_*_INT_ and *P*o_2_^S^ *_I_*_em_ values are in the 0.03–0.15 range and agree within experimental error, suggesting that no static quenching of the excited CNPs occurs in the presence of molecular oxygen.^[^ ^41^ ^]^

Transient absorption spectroscopy experiments with CNPs 1–5 in a 2‐propanol‐glycerol mixture (1:1, v/v) revealed the formation of long‐lived transient species. Under Ar‐purged conditions, lifetimes (*τ*_T0_) in the 5–30 µs range could be observed, which were reduced in the presence of oxygen and thus were assigned to triplet excitons (**Table** **3**). From the decay traces of each sample, the lifetimes of the transients in air‐equilibrated and O_2_‐purged conditions were determined, allowing the estimation of the dynamic bimolecular quenching rate constant of the triplet excitons of the CNPs by ground‐state molecular oxygen (Table 3; Figure S19, Supporting Information). The rate constants for triplet exciton quenching (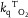) are one order of magnitude lower than those of singlet exciton quenching (Table S8, Supporting Information, 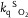), in good agreement with the spin‐statistical effect on the diffusion‐controlled oxygen quenching of molecular triplet and singlet excited states, respectively.^[^ ^42^ ^]^ Concomitant production of singlet oxygen (^1^O_2_) was observed by time‐resolved detection of the ^1^O_2_ emission at 1270 nm. The signals observed were unequivocally attributed to ^1^O_2_ since complete depletion was achieved upon addition of NaN_3_ (25 mm), a well‐known ^1^O_2_ quencher.^[^ ^44^ ^]^ Moreover, the lifetime of photosensitized ^1^O_2_ was measured at two excitation wavelengths with values of 19.6 ± 0.9 µs and 20.1 ± 0.6 µs, at 355 and 473 nm excitation wavelengths, respectively. While showing good agreement with the reported value of 22.3 µs,^[^ ^45^ ^]^ the slightly shorter lifetimes suggest some quenching of ^1^O_2_ by the CNPs themselves. The probability of triplet exciton trapping by O_2_ (*P*o_2_^T^) under air‐equilibrated conditions is almost quantitative, in good agreement with the long triplet exciton lifetimes in oxygen‐free solutions and a diffusional quenching rate constant. Finally, the ^1^O_2_ production quantum yield (*Φ*_Δ_), measured at two excitation wavelengths (355 and 473 nm), showed a strong dependence on the excitation wavelength, unlike what was observed for *Φ*_em_. Thus, *Φ*_Δ_ values determined with visible excitation showed a ≈10‐fold decrease relative to those determined under UV. Interestingly, the highest photosensitization quantum yields are shown by CNPs prepared from benzene, toluene, and aniline, revealing a notable influence of the chosen precursor.

**Table 3:** CNPs triplet lifetimes from transient absorption experiments of the CNPs 1–5 in a 2‐propanol‐glycerol mixture (1:1, v/v) under Ar‐purged, air‐equilibrated, and O_2_‐purged conditions; dynamic bimolecular quenching rate constant by O_2_, probability of quenching by O_2_, and singlet oxygen photosensitization quantum yields.

Sample	% O_2_	*τ*_T_ [µs]^a)^	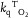 [m ^−1^s^−1^]^b)^	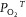	*Φ*_Δ_ ^c)^	*Φ*_Δ_ ^d)^
CNP 1	0	5.0 ± 0.1	5.6 10^8^ (± 4%)			
	21	0.83	*R* ^2^ 0.9985	0.83	0.37	0.04
	100	0.17				
CNP 2	0	15.3 ± 0.5	6.9 10^8^ (± 1%)			
	21	0.66	*R* ^2^ 0.9999	0.96	0.35	0.02
	100	0.14				
CNP 3	0	30.6 ± 0.6	6.7 10^8^ (± 3%)			
	21	0.77	*R* ^2^ 0.9992	0.97	0.28	0.01
	100	0.15				
CNP 4	0	17.4 ± 0.3	2.6 10^8^ (± 10%)			
	21	1.10	*R* ^2^ 0.9906	0.94	0.09	0.01
	100	0.35				
CNP 5	0	21.4 ± 0.8	3.6 10^8^ (± 1%)			
	21	1.16	*R* ^2^ 1.0000	0.95	0.24	0.04
	100	0.27				

^a)^ *λ*_exc_ 355 nm, *λ*_det_ 600 nm;

^b)^ ± % Relative error in parentheses, *R* ^2^ of linear regression fit of Stern‐Volmer plots. Oxygen solubility values as in neat 2‐propanol have been assumed;

^c)^ *λ*_exc_ 355 nm. Experimental uncertainty ± 20%;

^d)^ *λ*_exc_ 473 nm. Experimental uncertainty ± 20%.

Lastly, by using the data in Tables 2 and 3, and under the assumption that every triplet quenching‐by‐oxygen event leads to the production of singlet oxygen, we have estimated the values of the triplet quantum yield (*Φ*_T_), the rate constant for internal conversion (*k*_ic_), the rate constant for intersystem crossing (*k*_isc_), and the quantum yield for internal conversion (*Φ*_ic_) of CNPs 1–5 under the same experimental conditions (*λ*_exc_ 355 nm, Table S9, Supporting Information). Further support to our previous deductions regarding the correlation between reaction substrates, structural and photophysical properties can be obtained from these results, since higher intersystem crossing rate constants and triplet quantum yields but lower internal conversion rates and quantum yields are observed in the case of hydrocarbon‐derived CNPs.

## 3 Conclusion

The intriguing and unexplored bottom‐up synthesis of carbon nanostructures from air‐equilibrated liquid organic precursors under unfocused nanosecond pulsed laser irradiation with visible or NIR light has been investigated.

The most relevant relationships found in this research are presented below. These correlations refer to the type of organic precursor used, the reaction mechanism, and the kinetics of CNP formation by nanosecond pulsed laser synthesis, as well as the resulting photophysical properties of the produced nanomaterial:

- The non‐focused nanosecond pulsed laser irradiation of air‐equilibrated liquid organic aromatic precursors causes photothermal decomposition of the molecular entities in the hot spots generated on the solid surface of the reaction vessel or of added microparticles (NiO). The subsequent zero‐order reactions depend on the structure and reactivity of the organic precursors and on the irradiation light parameters. More energetic photons (532 nm) drive more efficient reactions compared to photons with less energy (1064 nm). Furthermore, suitable flux densities of laser light, above an irradiance threshold of ≈2 W cm^−2^, are required for the generation of CNPs.
- The dark evolution under kinetic control of the reaction intermediates may require an induction time, especially for N‐containing precursors with electron‐rich aromatic structures (aniline, pyrrole), that require moderate induction periods, or even longer induction times in the case of precursors based on aromatic hydrocarbons (toluene, benzene). No induction time is required if less electron‐rich aromatic substrates are used (chlorobenzene or thiophene), particularly if they contain labile covalent bonds.
- However, despite the need for an induction time in some cases, aromatic hydrocarbons display faster kinetics for CNPs formation than less activated aromatics such as chlorobenzene or thiophene, and even faster than electron‐rich substrates like aniline and pyrrole. The presence of a reactive Csp^3^ in toluene improves the reaction kinetics. On the other hand, heteroatom‐containing substrates slow down the formation of CNPs, since the heteroatoms extruded from the broken molecular precursor do not generally participate in the formation of reactive intermediates that are finally incorporated into the resulting CNPs, but promote the creation of structural defects. As a consequence, the resulting CNPs contain a lower % of heteroatom than the organic precursor, and the relevant presence of N atoms in the CNPs has only been observed in the case of heterocyclic pyrrole.
- The use of microparticles of high melting point inorganic additives, as green NiO that absorbs light, allow the achievement of higher reaction temperatures (near 2000 °C) in the laser impact zone, promoting kinetics without induction period for aromatic hydrocarbons, which are as fast as those occurring with substrates containing labile bonds.
- Important changes in carbon hybridization, from ≈100% Csp^2^ in the aromatic molecular precursor to between 30–60% Csp^3^ in the resulting carbon nanoparticles, occur during the formation of the CNPs. This is due to the kinetic control conditions prevailing in the reaction medium. As a result, defect‐rich CNPs of the CND type (with high content of Csp^3^ and Csp^2^/Csp^3^ ratios <1) are generally obtained, especially from heteroatom‐containing substrates or from hydrocarbons containing Csp^3^, like toluene, regardless of the presence of the NiO additive. Csp^2^‐based benzene, on the other hand, promotes the additional formation of c‐GQDs, with a Csp^2^/Csp^3^ ratio >1 and with less structural defects than CNDs.
- The morphology of the CNPs varies from nearly spherical to discoidal shapes in the 1–11 nm interval. Toluene, aniline, pyrrole, and thiophene precursors tend to produce disk‐shaped nanoparticles, while the experiments with benzene, toluene‐NiO, and chlorobenzene yield quasi‐spherical nanomaterials. When the carbon nanostructures are dispersed in organic solvents, there is a dynamic equilibrium between isolated nanoparticles and their aggregates, which reach sizes ranging from tens to hundreds of nanometers.
- The nanoparticles are composed mainly of C, H, and O, with a small incorporation of heteroatoms from the organic precursor. Important incorporation of oxygen atoms into the CNPs (up to ≈20%) basically occurs due to etching of the reaction vessel surface (promoting some minor incorporartion of Si as well). The use of NiO additive minimizes the incorporation of O to the CNPs, below 5%, but maximizes the %Csp^3^, up to ≈60%, highly impacting the surface hydrophobicity of the resulting CNPs and minimizing the aggregation of these CNPs in nonpolar aromatic solvents.
- The CNPs display strong UV light absorption, and the absorption of visible light is modulated by the presence of heteroatoms from the organic precursor. Excitation and emission spectra vary with the corresponding emission or excitation wavelength selected, respectively, and redshifted emission bands are observed for the samples with higher thermal stability, prepared from toluene‐NiO, aniline, and pyrrole.
- Conversely to the wavelength‐dependent spectral changes observed in all the CNPs, wavelength‐independent fluorescence quantum yields have been measured. This is due to the balance between both, the wavelength‐dependent emission lifetimes and the unimolecular deactivation rate constants, *k*_r_ and *k*_nr_, whose values depend on the Csp^2^/Csp^3^ ratio in the CNPs. Therefore, the c‐GQDs and CNDs with less structural defects (i.e., with greater Csp^2^/Csp^3^ ratios) like those prepared from benzene or toluene, respectively, exhibit higher *k*_r_ but lower *k*_nr_ values, determining their higher *Φ*_em_ values and fluorescence lifetimes, as well as higher triplet quantum yields.
- The nanosecond‐lived singlet and microsecond‐lived triplet excitons of the CNPs are efficiently quenched by molecular oxygen, with dynamic bimolecular deactivation rate constants close to their respective diffusion‐controlled limits. Furthermore, the triplet excitons can photosensitize singlet oxygen production with moderate quantum yields, mainly in the case of CNPs produced from aromatic hydrocarbons. Conversely to the constant fluorescence quantum yields, *Φ*_Δ_ values depend on the excitation wavelength.

Our experimental results and the hitherto unknown correlations between organic precursors subjected to unfocused pulsed laser reactions and the structural and photophysical properties of the resulting CNPs, which we have now established for the first time, pave the way for the development of applications based on the rich photophysics of these new nanomaterials, whose properties can be tuned by a judicious choice of the most suitable organic substrates.

## Conflict of Interest

The authors declare no conflict of interest.

## Supporting information



Supporting Information

## Data Availability

The data that support the findings of this study are available from the corresponding author upon reasonable request.
