# Supplementary material for: Pulsed Laser Synthesis of Carbon Nanostructures from Organic Molecular Liquids: Structure, Kinetics and Photophysical Properties
Source: Adv Sci (Weinh). 2025 Jul 6;12(37):e05883. doi: 10.1002/advs.202505883 (PMC12499501; doi:10.1002/advs.202505883)
Supplement: Supplementary file 1 — Supporting Information [file ADVS-12-e05883-s001.docx]

Supporting Information

Pulsed Laser Synthesis of Carbon Nanostructures from Organic Molecular Liquids: Structure, Kinetics and Photophysical Properties

Antonio Ribeiro-González,^#^ Carlos Agudo-Blanco,^#^ Sergio Ramírez-Barroso,^#^ Cristina Navío, Luis Bañares, Roger Bresolí-Obach, Santi Nonell, Nazario Martín* and David García-Fresnadillo*

A. Ribeiro-González, C. Agudo-Blanco, S. Ramírez-Barroso, N. Martín, D. García-Fresnadillo

Department of Organic Chemistry, Faculty of Chemical Sciences
Universidad Complutense de Madrid
Avenida Complutense s/n, 28040 Madrid, Spain

E-mail: [nazmar@ucm.es](mailto:nazmar@ucm.es), [dgfresna@ucm.es](mailto:dgfresna@ucm.es)

C. Navío, L. Bañares, N. Martín

IMDEA-Nanoscience
Campus de Cantoblanco, 28049 Madrid, Spain

L. Bañares

Department of Physical Chemistry, Faculty of Chemistry, Universidad Complutense de Madrid, Avenida Complutense s/n, 28040 Madrid, Spain

R. Bresolí-Obach, S. Nonell

Institut Químic de Sarrià, Universitat Ramon Llull, Via Augusta 390, 08017 Barcelona, Spain

**Table of contents**

1. Experimental section and methods S3

**2. TEM images and histograms** S10

**3. STEM images** S14

**4. Raman spectra** S18

**5. AFM images and histograms** S20

**6. Graphs of DLS experiments** S33

**7. FTIR spectra** S36

**8. XPS spectra** S40

**9. TGA plots** S43

**10. Kinetics of carbon nanoparticles formation** S47

**11. UV-vis. absorption spectra** S50

**12. UV-vis. excitation spectra** S52

**13. UV-vis. emission spectra** S54

**14. Emission lifetimes, deactivation rate constants and Stern-Volmer plots** S57

**References** S65

1. Experimental section and methods

*Synthesis and purification of CNPs*: The organic precursors used in the pulsed laser synthesis experiments were benzene (HPLC grade, ≥99.8% Fisher Chemical or ≥99.9% Sigma-Aldrich), toluene (HPLC grade, ≥99.8% Fisher Chemical), chlorobenzene (+99% Thermo Scientific Chemicals), aniline (99% PanReac), pyrrole (98% Sigma-Aldrich), and thiophene (≥99% Sigma-Aldrich). Thorough purification of aniline was performed by addition of KOH to adsorb impurities and remove moisture, followed by vacuum distillation over KOH. Pyrrole was also purified by vacuum distillation over CaH_2_. Thiophene was purified from fluorescent stabilizers by vacuum distillation over KOH and subsequently protected from light.^[46]^ These three precursors were kept under vacuum until irradiation with the pulsed laser, to avoid the formation of dark impurities. An additional experiment with toluene was carried out in the presence of nickel(II) oxide green powder (NiO, 400 mesh, <37 μm diameter, Alfa Aesar, mp 1984 ºC) to check the influence of these microparticles (with similar light absorption at 1064 or 532 nm)^[47]^ on the formation of the CNPs with a non-focused laser beam,^[25a,c]^ compared to the experiments performed with no additive in the colorless and transparent quartz or borosilicate 3.3 glass vessels used as the reactant containers (mp 1715 ºC and 1648 ºC for quartz and borosilicate 3.3 glass, respectively). The solvents used to purify and characterize the carbon nanoparticles were distilled and deionized water, diethyl ether (99.7% Panreac) and HPLC-grade methanol (≥99% Fisher Chemical), 2-propanol (≥99.5% Carlo Erba), 1-butanol (99% Fisher Chemical), glycerol (≥99% Sigma Aldrich), acetone (HPLC grade, ≥99.8% Fisher Chemical) and dimethylsulfoxide (≥99.7%, HPLC Sigma-Aldrich).

A Nd-YAG (Continuum Surelite, 10 Hz, 6 ns pulse width and 6 mm beam diameter) providing laser radiation either of 1064 or 532 nm (2nd harmonic) was used in the pulsed laser experiments to synthesize carbon nanoparticle samples from the different molecular organic precursors at 25 ± 2 ºC. No laser beam focusing system was used during the experiments. The non-focused laser beam was appropriately guided with a suitable combination of mirrors to irradiate the stirred and air-equilibrated samples from their top side (Scheme S1). Some experiments where the kinetics of carbon nanoparticle formation was studied in detail were performed in a standard 1 × 1 cm quartz cell for spectrofluorimetry filled with 3 mL of precursor at 25 ± 2 ºC, while the rest of pulsed laser synthesis experiments were carried out with a home-made photochemical reactor at 25 ± 2 ºC. The emission from the quartz cell was guided with an optical fiber to a CCD device (Ocean Optics) for fluorescence signal detection. The home-made reactor consists of a metal structure that comprises three parts: i) a cylinder-shaped aluminum container lined with aluminum foil inside, where a borosilicate 3.3 glass vessel (5 cm outer diameter, 4 cm height) is placed; ii) a stainless-steel lid, with two holes each covered by a rubber septum where a temperature probe, gas purge or sampling system can be fitted, and a replaceable pyrex glass window (90% transmittance in the range 2000–350 nm) that allows the laser beam to irradiate the reaction mixture; and iii) a stainless-steel clamp to fit the container with the lid. The reactant was left to reach ambient temperature (25 ± 2 ºC) while magnetically stirred with a Teflon-coated magnet (600 rpm for liquid samples or 1200 rpm for NiO experiments) and, once the photoreactor was sealed, the system was typically irradiated for 6 hours with the laser beam eccentric to the magnetic stirring axis, in order to avoid any damage of the magnetic stirrer by the laser beam (see Supporting Information and XPS details on sample CNP 7 in Tables S2 and S3). The typical temperature reached by the reactor at the end of the experiments was 33 ± 2 ºC. Yellowish to brownish reaction crudes were obtained from these experiments.

**Scheme S1.** Experimental set-up of the pulsed laser synthesis experiments for the preparation of CNPs. Experiments performed with a quartz cell allowed the *in situ* study of the reaction kinetics by detecting the fluorescence changes of the reaction crude due to the generated carbon nanoparticles. In the case of the photochemical reactor, 300 μL aliquots were drawn from the reactor and diluted to 3 mL total volume with 2-propanol. Experiments carried out with the photochemical reactor allowed the preparation of larger amounts of nanomaterial. M stands for the mirrors used to guide the non-focused laser beam and irradiate the organic precursor.

Regarding the purification of the nanomaterials, the carbon nanoparticles were isolated from the remaining liquid precursor and from potential molecular impurities by rotavaporation to dryness or vacuum distillation (in the case of the experiments with aniline, pyrrole and thiophene). Subsequently, the solid product was suspended in diethyl ether followed by centrifugation (3×10 min., 6000 rpm) to remove any impurity soluble in diethyl ether, finally, the nanomaterial was recovered by rotavaporation of the remaining solvent, followed by vacuum drying in a dessicator containing a capsule with P_2_O_5_. In the case of the NiO-toluene experiment, before crude rotavaporation, an additional filtration process with a Fluoropore 0.2 μm pore filter (Merck) was used to remove the nickel(II) oxide microparticles. TLC (hexane/ethyl acetate, 3:2, 2:3 or 1:1, v/v) and FTIR analysis evidenced the different nature of the isolated nanomaterial and complete removal of the organic precursor. Experiments of pulsed laser synthesis of carbon nanoparticles were performed in triplicate at least, and 10–100 mg of isolated nanomaterial was typically obtained with the photochemical reactor. Once purified, the carbon nanoparticles were finally dissolved in 2-propanol, toluene or dimethyl sulfoxide for further characterization.

*Structural characterization of CNPs*: Transmission Electron Microscopy (TEM) measurements were performed with a JEOL JEM 2100 apparatus (acceleration voltage 200 kV) on holey carbon-Cu, 200 mesh, 50 micron supports (Electron Microscopy Sciences). In order to estimate the Csp^2^/Csp^3^ content by the signatures of the π* and σ* bonds at 282-288 eV and 290-320 eV, respectively,^[48]^ Scanning Transmission Electron Microscopy (STEM) combined with electron energy loss spectroscopy (EELS spectrometer, Quantum GIF) was carried out using an aberration corrected JEOL JEM ARM200 (with a cold Feld Emission Gun (FEG) using a voltage of 80 kV. Electron microscopy was carried out at the ICTS-CNME at the University Complutense of Madrid campus. ImageJ 1.54d software is used for particle counting, with a minimum of 200 particles for each sample.

Atomic force microscopy (AFM) measurements were carried out in a commercial AFM system (Ntegra Prima, NT-MDT) using semicontact (dynamic) scanning mode in ambient conditions. Rectangular aluminum coated cantilevers HQ:NSC15/Al BS (Mikromash) were used, with a tip radius <8 nm. Their nominal spring constant is 40 N/m and its resonance frequency is around 325 kHz. The samples were prepared by spin coating in DMSO/H_2_O on a mica support. Analysis and background correction was performed with Gwyddion 2.64 and WSxM 5.0 Develop software programs.^[49]^

The hydrodynamic size of the carbon nanostructure aggregates was measured by dynamic light scattering (DLS) from dilute suspensions of the samples in 2-propanol and toluene in a standard cuvette, using a Zetasizer NanoZS device (Malvern Instruments). All measurements were performed in triplicate.

Raman spectroscopy measurements were performed on a SiO_2_/Si (001) crystal with a Senterra II Raman microscope (Bruker), which combines a Raman spectrometer equipped with 532, 633 and 785 nm laser lines and a confocal microscope module (Olympus BX51). Data analysis was done with OPUS software.

The Fourier transform infrared (FTIR) spectra were obtained using a Bruker Alpha-T equipped with an attenuated total reflection (ATR) accessory. Acquisition was made either from samples dispersed in volatile organic solvents such as methanol or acetone deposited on an ATR accessory or, when necessary for quality reasons, from KBr pellets. Data analysis was done with OPUS software.

X-ray photoelectron spectroscopy (XPS) measurements were performed using a monochromatic Al-k_α_ as excitation source (1486.7 eV). The ejected electrons were collected in a hemispherical electron analyzer (SPHERA U7), using a pass energy of 20 eV to have a resolution of 0.6 eV. A flood gun (Specs FG-500) was used to compensate the possible built-up charge (3 eV, 40 μA) and all the spectra have been corrected in binding energy, taking the sp^2^ component of the C 1s core level centered at 284.5 eV as the binding energy reference. The preparation of the samples for the XPS measurements has been twofold: (i) by attaching the powder samples on a double-sided C conductive tape, or (ii) by dropping the sample/solution on a SiO_2_/Si (001) crystal. This last method has been used for samples CNP 3, CNP 5 and CNP 7, and for their atomic percentage quantification, all the Si signal (with its corresponding associated O) has been removed.

Thermogravimetric analysis (TGA) measurements were performed with a TGA Q500 apparatus (TA instruments) by heating around 1 mg of sample in platinum pans under N_2_ current (≥ 99.999 %, 40 mL/min), using an isotherm of 30 minutes at 100 °C in order to eliminate any solvent residue from the nanoparticles, with a temperature ramp (10 °C min^–1^) up to 1000 ºC to ensure that heating is not too rapid.

*Photophysical characterization of CNPs*: UV-vis. absorption spectra of the samples previously sonicated for 15 minutes were recorded on UV-vis. spectrophotometers (Varian Cary 50 or Varian Cary 5000) using 1 cm path length quartz cuvettes (Suprasil). The absorption spectra have been corrected for Rayleigh scattering, when necessary, due to the tendency of the carbon nanoparticles to strongly interact forming aggregates. Correction of the absorption spectra was performed with the correction function shown in Equation 1:^[35]^

$A=\log\left[ \frac{1}{1-c\lambda^{4}} \right]$ (1)

where, *A* is the absorbance, *c* is a proportionality constant (10^9^) and *λ* is the wavelength of light in the UV-vis. region used for recording the spectra (200–800 nm, with negligible light absorption by the samples in the 700–800 nm interval). The correction function was subtracted from the experimental optical density and this spectral correction allowed the determination of several features (peaks or shoulders) in the UV end of the spectra below 360 nm.

Room-temperature photoluminescence (PL) and photoluminescence excitation (PLE) spectra of the samples previously sonicated for 15 minutes were acquired on a spectrofluorometer (Horiba FluoroLog 3) equipped with a high-pressure Xenon lamp and a Hamamatsu R928P photomultiplier tube; the PLE and PL spectra were corrected for the characteristics of the lamp source and of the detection system, respectively. Most spectra were acquired with air-balanced solutions, and some samples were purged with Ar (5.0, Linde) or O_2_ (5.0, Linde).

The fluorescence quantum yields of the Ar-purged samples (*Φ*_em_) were determined at room temperature (25 ± 2 ºC) using different excitation wavelengths (355, 375, 405 and 457 nm) relative to quinine sulfate (≥99 % BioReagent, suitable for fluorescence, Aldrich) with *Φ*_em_ = 0.55 ± 0.05 in 0.5 M H_2_SO_4_ (95-98 % Scharlau),^[50]^ or 2,5-diphenyloxazole (scintillation grade, Acros Organics) with *Φ*_em_ = 0.842 ± 0.042 in cyclohexane (HPLC-grade, ≥99 % Thermo Scientific Chemicals),^[51]^ and riboflavin (Sigma Aldrich) with *Φ*_em_ = 0.30 ± 0.03 in methanol (HPLC-grade, ≥99 % Fisher),^[52]^ respectively. Identical emission wavelength ranges were used for all the samples to determine the areas under the spectral curves. The optical density (≤0.1 at *λ*_exc_) of each solution was checked before and after collection of the corresponding emission spectrum. In these conditions, the unknown *Φ*_em_ values were calculated by using Equation 2:

$\Phi_{em}=\Phi_{emRe f}\frac{I}{I_{Re f}}\frac{A_{Re f}}{A}\frac{n^{2}}{n_{Re f}^{2}}$ (2)

where *Ι* is the integrated area of the emission intensity under the spectral curve, *A* is the absorbance at the excitation wavelength, and *n* is the refractive index of the solvent (1.332988 for water, 1.329 for methanol, 1.3756 for 2-propanol and 1.42623 for cyclohexane),^[53]^ and *Ref* stands for the corresponding values of the reference standard.

Time-resolved fluorescence experiments (TRF) were performed with a custom-made PicoQuant Fluotime 200 fluorescence lifetime system. Fluorescence spectra were acquired with excitation at 290, 457 and 502 nm by pulsed LEDs (PLS-290, PLS-450, PLS-500, PicoQuant, and at 375 nm and 405 nm by picosecond pulsed laser diodes working at a repetition rate of 10 MHz (LDH-P-C-375 and LDH-P-C-405, PicoQuant). Emission maxima were observed by keeping the count frequency below 1%. In cases of fluorescence emission with excitation at 375, 405 and 457 nm, measurements were made under Ar-purged, and at different O_2_ concentrations (air-equilibrated and O_2_-saturated). For all other excitation wavelengths, measurements were made only under air-equilibrated conditions. Fluorescence decays were analyzed using PicoQuant Fluofit v4.6.5 data analysis software using Equation 3 to fit the decays by a built-in deconvolution method:

$I(t)=\int_{-\infty}^{t} IRF(t'){\sum_{i=1}^{n} A_{i}e}^{-\frac{t-t'}{\tau_{i}}}dt'$ (3)

where *I(t)* stands for the fluorescence intensity at time *t*, *IRF(t')* is the instrumental response time function, *t'* takes a value of 0.08 due to the instrumental time delay, *A*_i_ are the pre-exponential factors, and *τ*_i_ are the discrete lifetime components of the multi-exponential fitting of the decay curve.

The amplitude-weighted (*τ*_AMP_) and intensity-weighted (*τ*_INT_) average emission lifetimes have been calculated taking into account, for each *i*_th_ lifetime component, the corresponding discrete amplitudes (*A*_i_, related to the population of the emissive species contributing to that fluorescence lifetime), and intensities (*I*_i_, where *I*_i_ = *A*_i_*τ*_i_, related to the greater or lesser contribution to the overall fluorescence by the given discrete species, i.e., its emission quantum yield). Therefore, *τ*_AMP_ = [Σ_i_(*A*_i_*τ*_i_)]/[Σ_i_(*A*_i_)] (for the apparent decay time at the first fitting range channel at time zero, excluding scattered light contributions), and *τ*_INT_ = [Σ_i_(*I*_i_*τ_i_*)]/[Σ_i_(*I*_i_)] (for the average arrival time of a photon after excitation pulse).

Similarly, the corresponding % *A*_i_ and % *I*_i_, expressed relative to the total number of species, for each *i*_th_ lifetime component, are % *A*_i_ *=* [*A*_i_/(Σ_i_*A*_i_)] ×100, and % *I*_i_ = [*I*_i_/(Σ_i_*I*_i_)] ×100, respectively.

From the *Φ*_em_ and *τ*_0AMP_ values (where the subindex 0 refers to lifetime measurements performed in the absence of any quencher), the radiative (*k*_r_) and nonradiative (*k*_nr_) deactivation rate constants were estimated as follows: *k*_r_ = *Φ*_em_ / *τ*_0AMP_; *k*_nr_ = (1 / *τ*_0AMP_) – *k*_r_.

From the Stern-Volmer plots for singlet and triplet excitons, the corresponding dynamic bimolecular quenching rate constants could be estimated, from stedy-state and time-resolved experiments, as follows: *I*_0_ / *I* = 1 + *K*_SV_ × [O_2_], for emission intensity measurements; or *τ*_0INT_ / *τ*_INT_ = 1 + *K*_SV_ × [O_2_], for time-resolved measurements; where *I*_0_ stands for the area under the emission spectrum curve in the absence of O_2_ quencher, and *I* stands for the area under the emission spectrum curve in the presence of O_2_ quencher; and *τ*_0INT_ stands for the intensity-weighted mean emission lifetime in the absence of molecular oxygen, and *τ*_INT_ stands for the intensity-weighted mean emission lifetime in the presence of molecular oxygen*. K*_SV_ stands for the Stern-Volmer constant, and *K*_SV_ = *k*_q_^S^_O2_*_I_*_em_ × *τ*_0INT_ or *K*_SV_ = *k*_q_^S^_O2_*_τ_*_INT_ × *τ*_0INT_ in the case of singlet excitons, while *K*_SV_ = *k*_q_^T^_O2_ × *τ*_0T_ for triplet excitons (see below).

Triplet exciton lifetimes were determined for Ar-purged samples by laser flash photolysis experiments with a Q-switched Nd-YAG laser (Surelite I-10, Continuum) at 355 nm excitation and with a Xe lamp (PTI, 75 W) at right-angle geometry for analysis. The white-light probe beam passed through a grating monochromator (mod. 101, PTI) observing at 600 nm, and was detected by a photomultiplier (R928, Hamamatsu) whose output was finally fed to a Lecroy Wavesurfer 454 oscilloscope.

Time-resolved phosphorescence detection of ^1^O_2_ emission at 1270 nm produced by the samples previously sonicated for 15 minutes was performed using a customized Fluotime 200 fluorescence lifetime system (PicoQuant, Germany) described elsewhere.^[54]^ Briefly, a diode-pumped Q-switched Nd-YAG laser (FTSS355-Q, Crystal Laser, Berlin, Germany) and a solid state AOM Q-switched laser (AO-Z-473, Changchun New Industries Optoelectronics Technology Co., China) were used to excite samples at 355 nm (1 kHz repetition rate) and at 473 nm (1 kHz repetition rate), respectively. To remove any residual component of its fundamental emission in the NIR region, a 1064-nm rugate notch filter (Edmund Optics, U.K.) and an uncoated SKG-5 filter (CVI Laser Corporation) were placed at the exit port of the laser. The NIR luminescence exiting from the side of the sample was filtered by a long-pass filter of 1000 nm and a narrow bandpass filter at 1270 nm. In this way any scattered laser radiation was removed and the NIR emission from singlet oxygen was isolated. A thermoelectrically-cooled Hamamatsu NIR sensitive photomultiplier tube assembly (H9170-45, Hamamatsu, Japan) coupled to a multichannel scaler (Nanoharp 250, PicoQuant) was used for single photon counting detection. The time-dependent ^1^O_2_ phosphorescence signal *S*(*t*) was analyzed using the GraphPad Prism 7 software to fit the data to Equation 4, in which *τ*_T_ and *τ*_Δ_ are the lifetimes of the photosensitizer triplet state and of ^1^O_2_ respectively, and *S*(0) is the phosphorescence signal at zero time, just after pulsed excitation, which is proportional to *Φ*_Δ_:

$S_{1270}(t)=S_{1270}(0)\times\frac{\tau_{\Delta}}{\tau_{\Delta}-\tau_{T}}\times\left( e^{-t/\tau_{\Delta}}-e^{-t/\tau_{T}} \right)$ (4)

The *Φ*_Δ_ values of the different samples were obtained by comparison of the slopes of *S*_1270_(0) *vs.* absorbed-laser-energy plots obtained at different sample and reference concentrations following Equation 5:

$\Phi_{\Delta,sample}=\Phi_{\Delta,Re f}\frac{slope_{sample}}{slope_{Re f}}$ (5)

Phenalen-1-one (*Φ*_Δ_ = 1.00 ± 0.05, in 2-propanol) and Rose Bengal (*Φ*_Δ_ = 0.75 ± 0.05, in 2-propanol) references were used for the excitation wavelengths of 355 and 473 nm, respectively.^[42, 44, 55]^ Oxygen solubility data in 2-propanol are 2.2 10^–3^ M and 10.3 10^–3^ M for 21% and 100% O_2_ equilibrated solutions, respectively.^[56]^

Under the assumption that every triplet quenching by oxygen event leads to the production of singlet oxygen, the triplet quantum yield values can be estimated as *Φ*_T_ = *Φ*_Δ_ / *P*_O2_^T^ (see reference 42). Then the rate constant for intersystem crossing can be derived as *k*_isc_ = *Φ*_T_ / *τ*_0AMP_. This allows to also calculate the rate constant for internal conversion as *k*_ic_ = (1 / *τ*_0AMP_) – *k*_r_ – *k*_isc_. And, from this value, the internal conversion quantum yield *Φ*_ic_ can be estimated as *Φ*_ic_ = *k*_ic_ × *τ*_0AMP_.

*Statistical Analysis:* The software OriginPro 2023b and SigmaPlot 11.0 have been used for the processing, plotting and statistical analysis of all the experimental data. Results of the analysis of TEM, AFM and DLS measurements were presented as mean ± standard deviation (Table 1 and Table S1). For TEM and AFM analysis, Gaussian or Lorentzian fitting functions are used depending on the quality of the fit (R^2^ ≥ 0.96). Also, descriptive statistics have been included for the AFM measurements (Table S1). Stern-Volmer plots allow the calculation of the dynamic bimolecular quenching rate constant by molecular oxygen, *k*_q_, where relative errors are obtained from linear regression of the data.

**2. TEM images and histograms**

| a  | b **** |
| --- | --- |
| c  | d  |
| e  | f  |
| g **** | h **** |
| i **** | j  |
| k **** | l **** |
| m **** | n **** |
| o **** | p **** |
| q **** | r **** |
| s **** | t **** |
| u **** | v **** |
| w **** |  |

**Figure S1.** TEM images and diameter histogram of CNPs prepared from benzene (CNP 1, a-d), toluene (CNP 2, e-g), toluene-NiO (CNP 3, h-j), chlorobenzene (CNP 4, k-m), aniline (CNP 5, n-q), pyrrole (CNP 6, r-t) and thiophene (CNP 7, u-w).

**3. STEM images**

| a 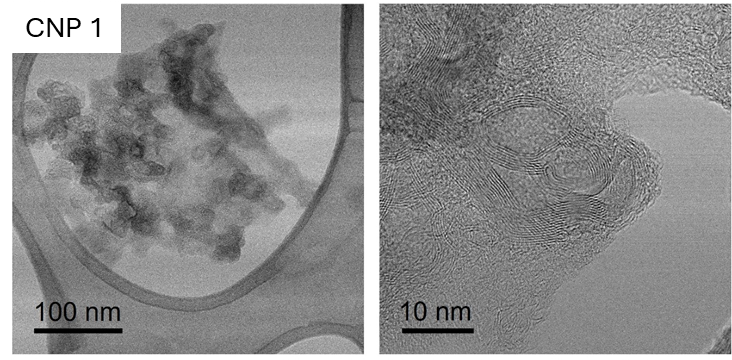 |
| --- |
| b  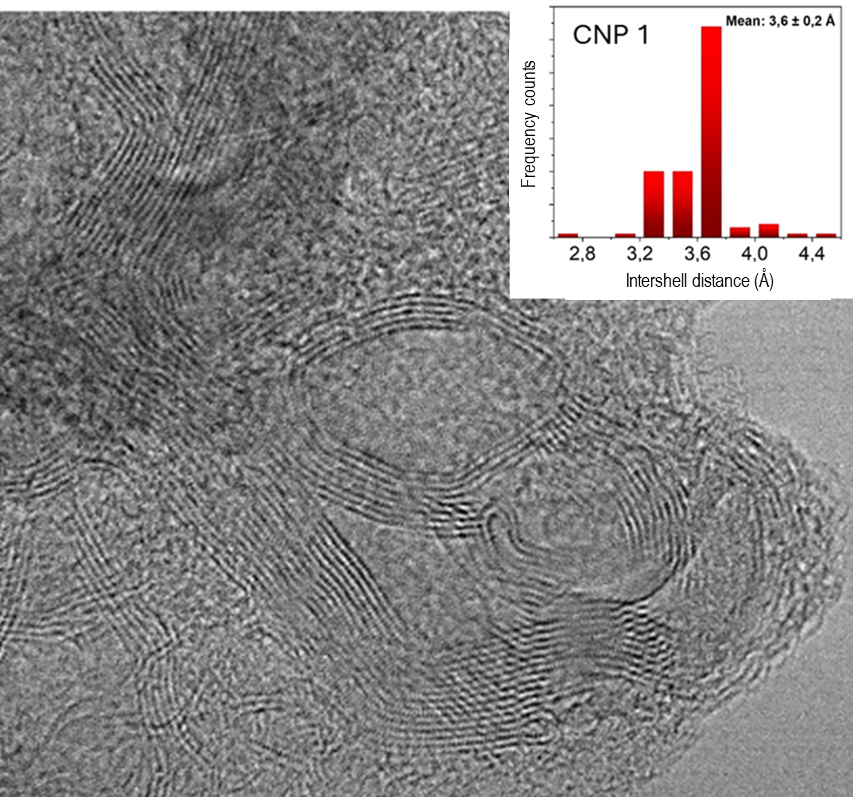 |
| c 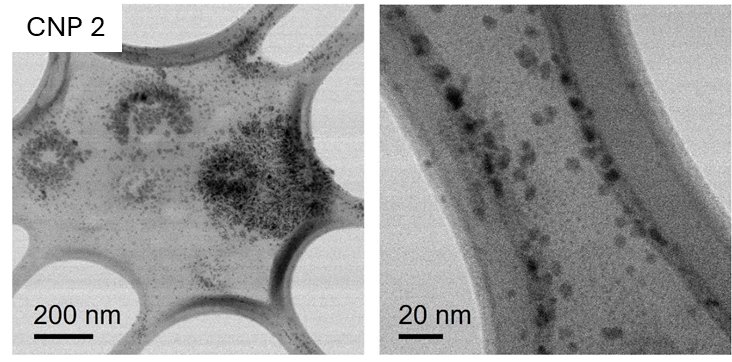 |
| d 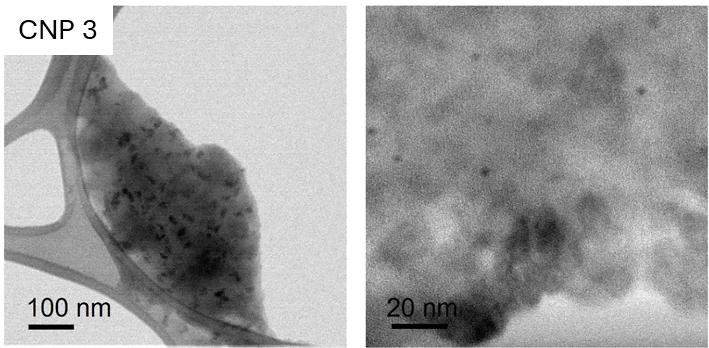 |
| e 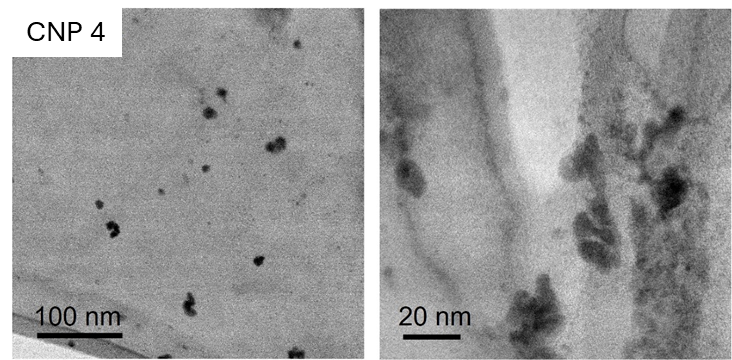 |
| f 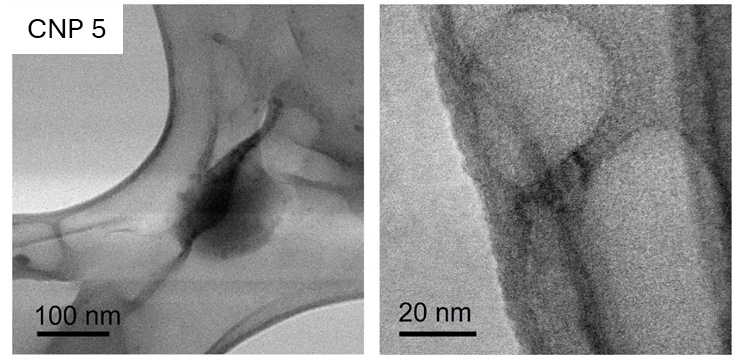 |
| g 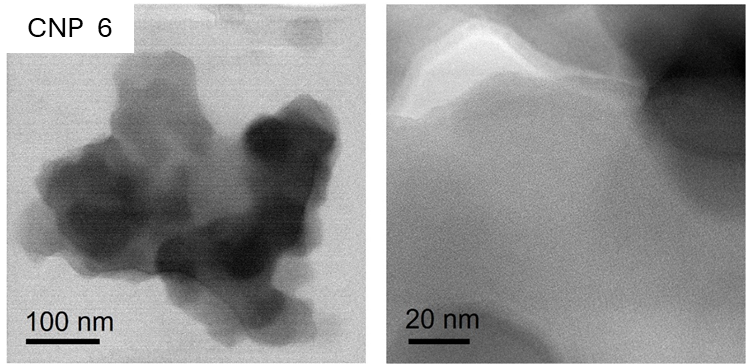 |
| h 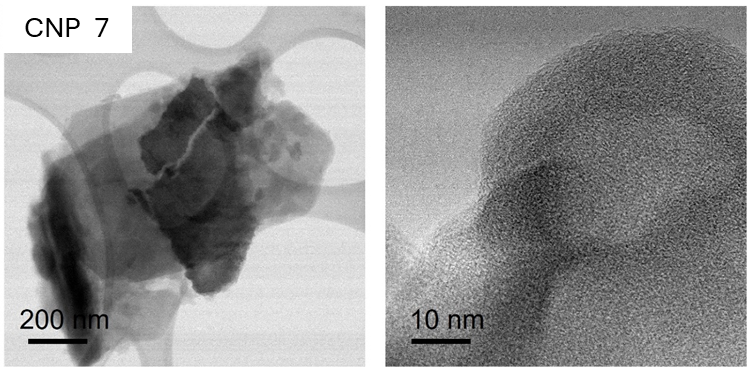 |

**Figure S2.** Low magnification (left) and high-resolution (right) ABF-STEM images of the CNPs prepared from benzene (CNP 1, a) and (b) detail of the nano-onion fragments observed in this sample (2–15 layers can be stacked) and its histogram showing the intershell distances determined from 115 observed layers (mean separation 3.6 Å), toluene (CNP 2, c), toluene-NiO (CNP 3, d), chlorobenzene (CNP 4, e), aniline (CNP 5, f), pyrrole (CNP 6, g) and thiophene (CNP 7, h).

**4. Raman spectra**

The intensities ratio (I_D_/I_G_) of the characteristic Raman bands was calculated after subtraction of the large background due to sample autofluorescence.

| a 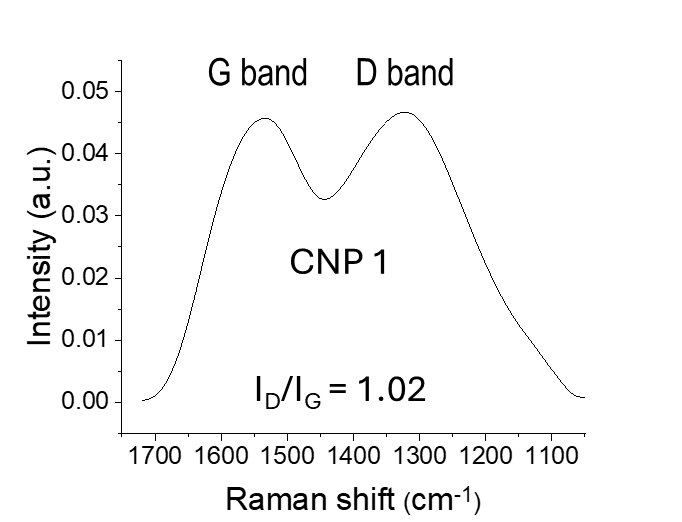 | b 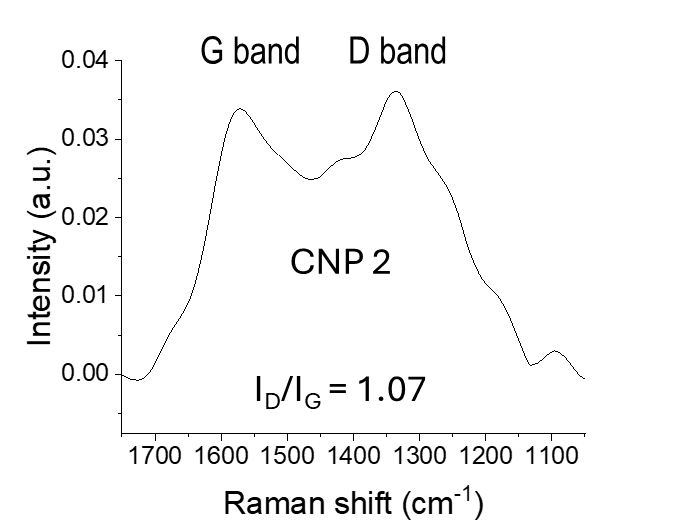 |
| --- | --- |
| c 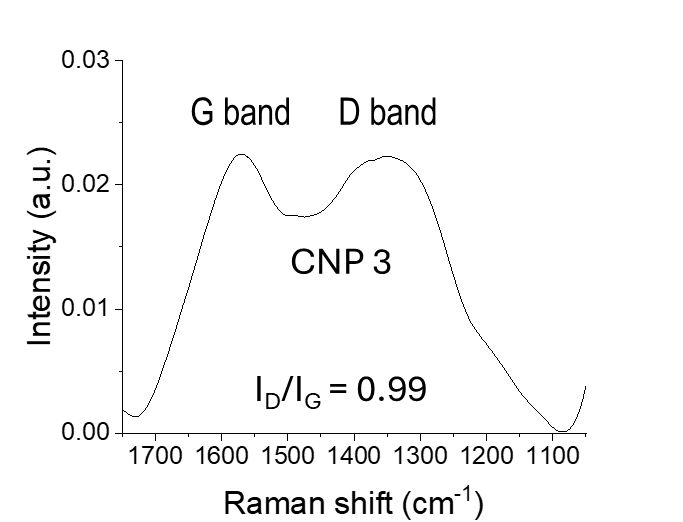 | d 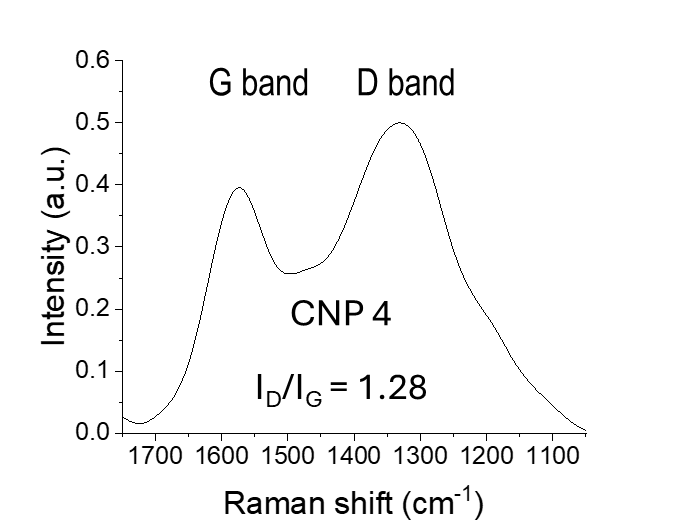 |
| e 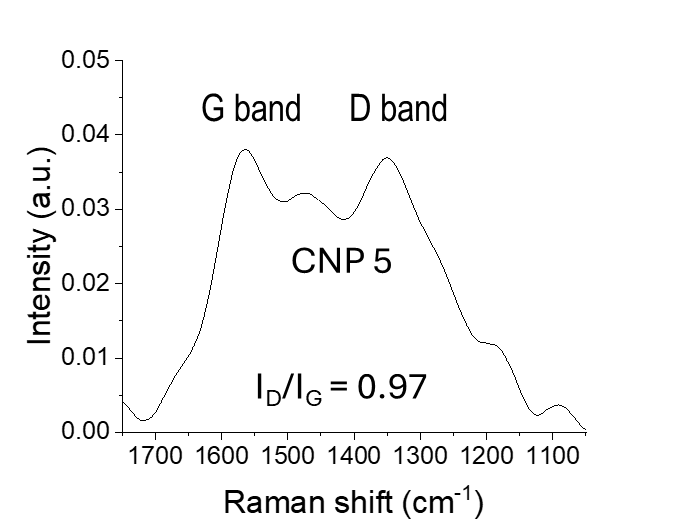 | f 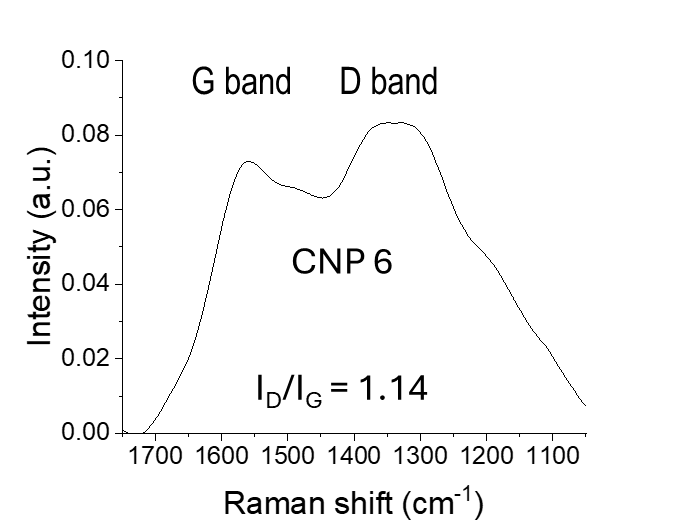 |
| g 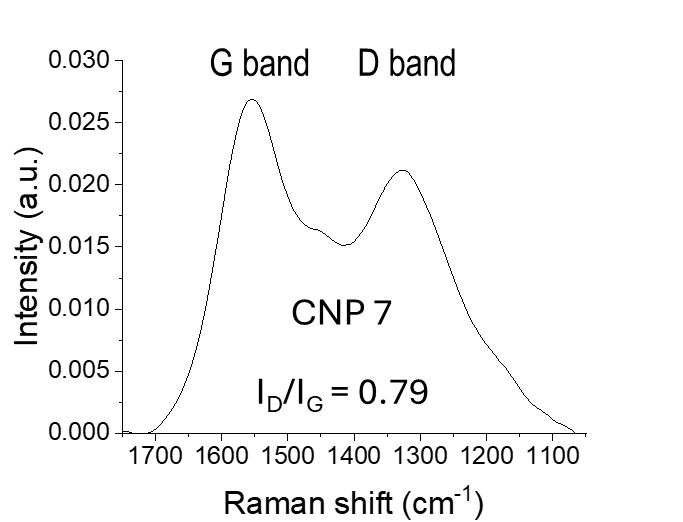 | h  |

**Figure S3.** Raman spectra (λ_exc_ = 633 nm) and I_D_/I_G_ ratio of the CNPs prepared from benzene (CNP 1, a), toluene (CNP 2, b), toluene-NiO (CNP 3, c), chlorobenzene (CNP 4, d), aniline (CNP 5, e), pyrrole (CNP 6, f) and thiophene (CNP 7, g). Figure (h) shows the Raman spectrum of the samples supported on SiO_2_/Si (001) crystal, showing the bands below 1000 cm^–1^ of the support.

**5. AFM images and histograms**

| a  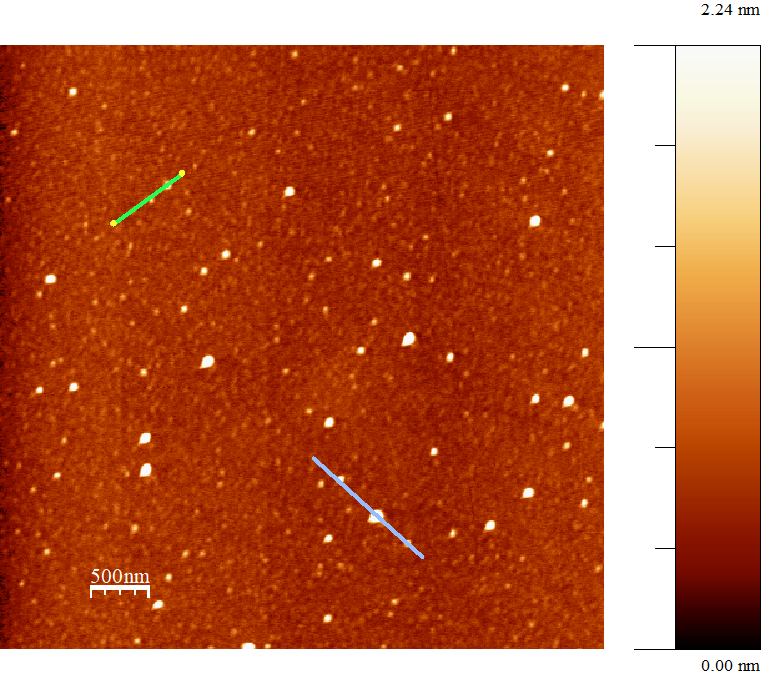    |
| --- |

| b  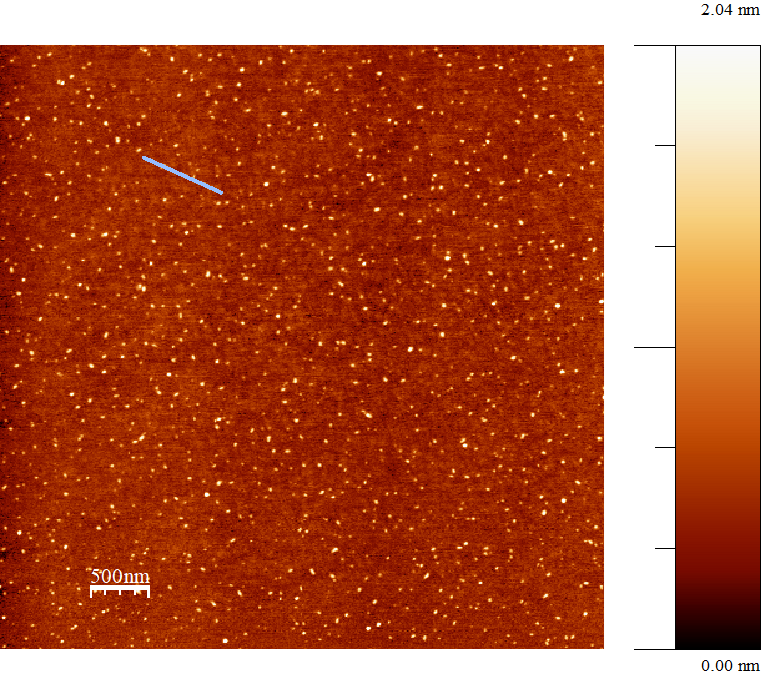 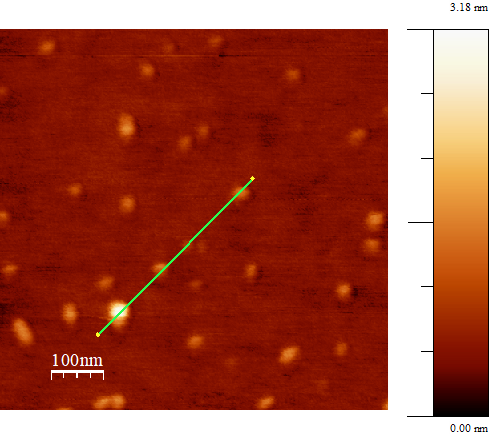  |
| --- |

| c  **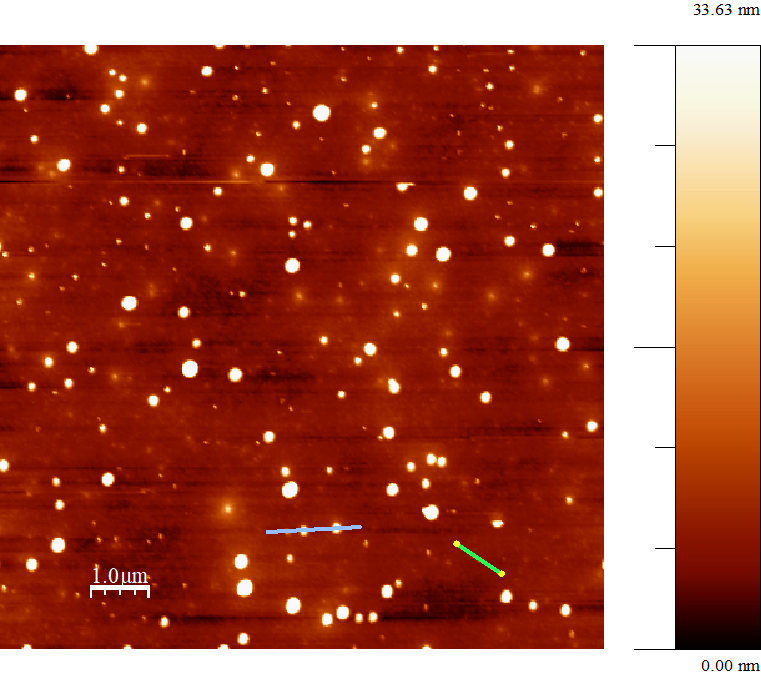** **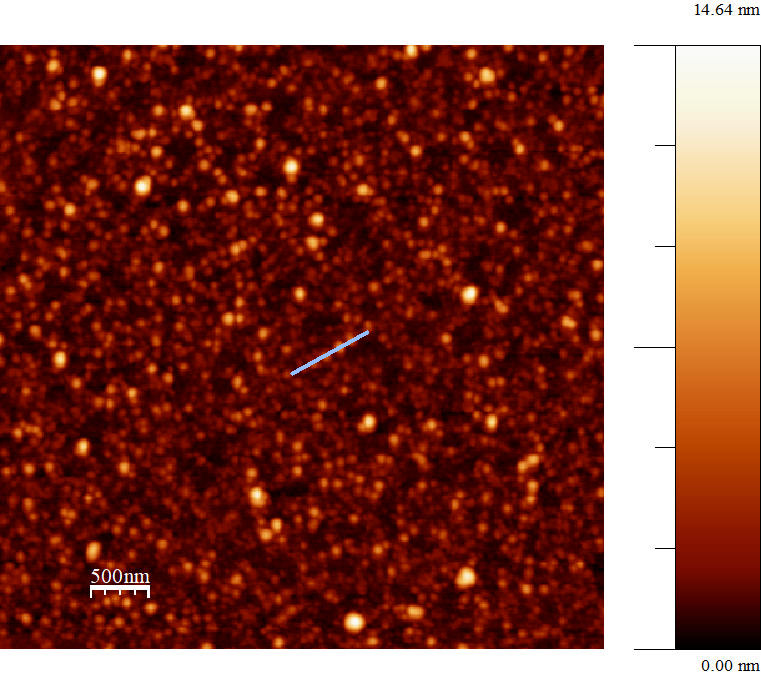** ****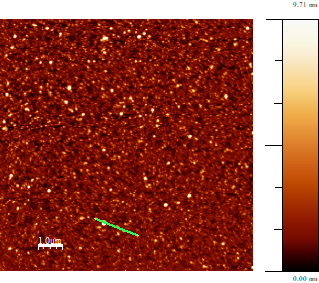 **** |
| --- |

| d  **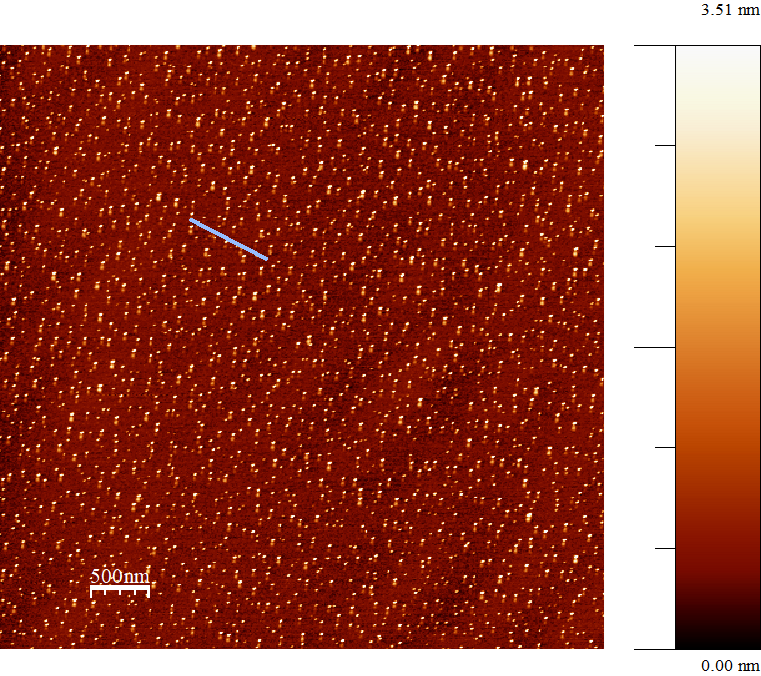** **** |
| --- |
| 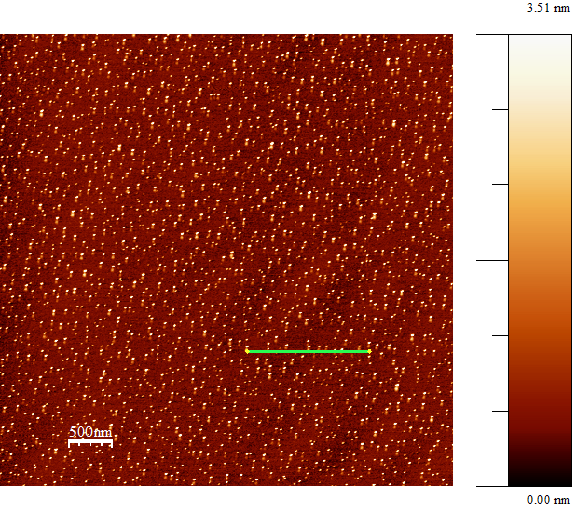  |

| e  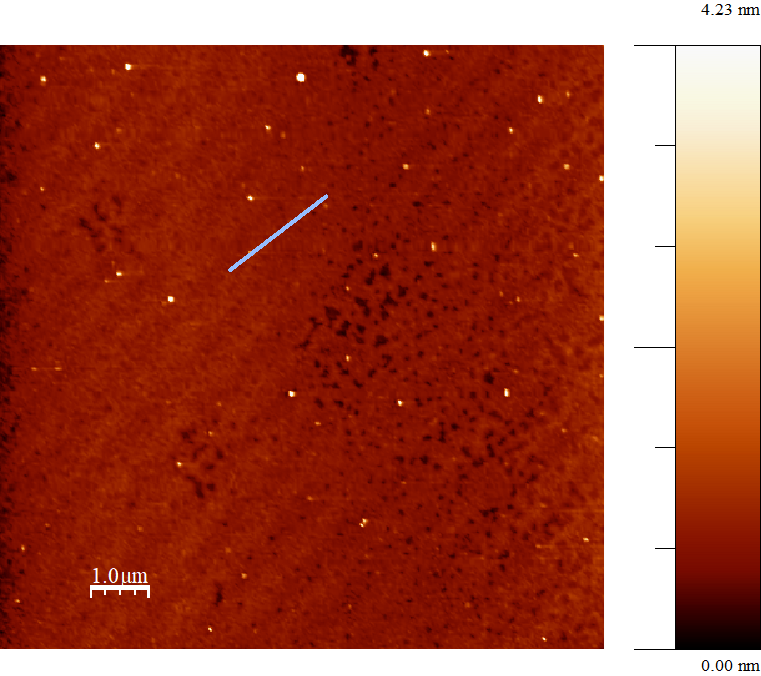 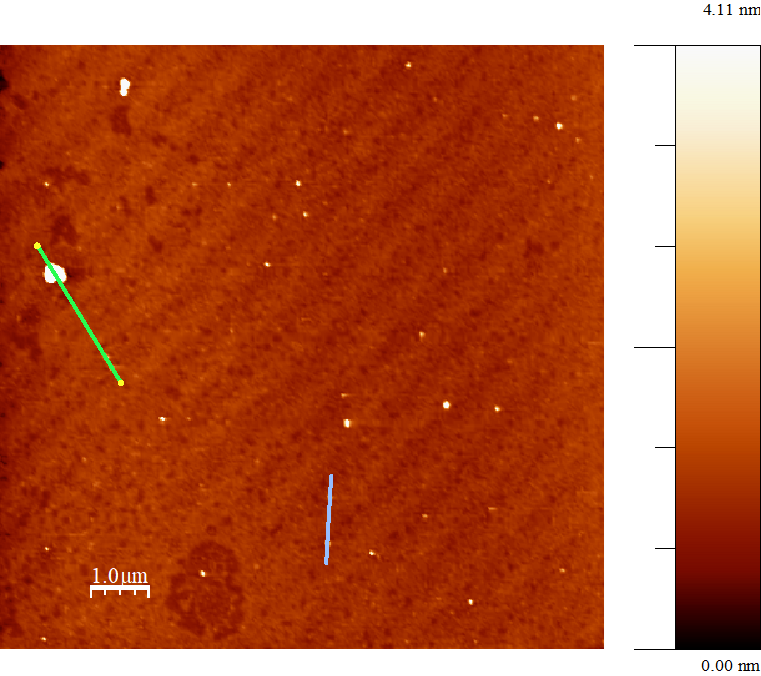 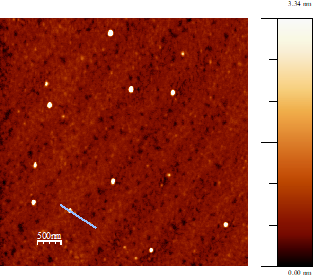  |
| --- |

| f  **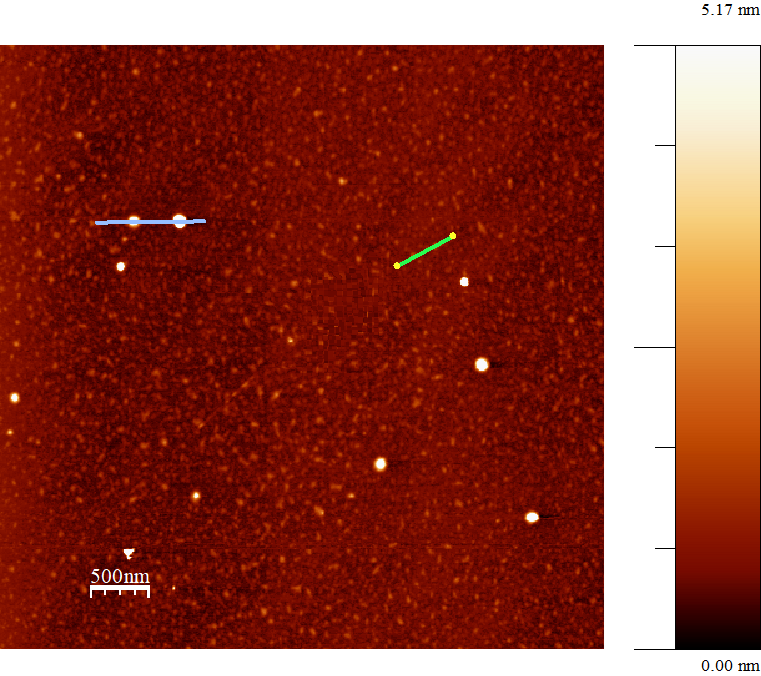** **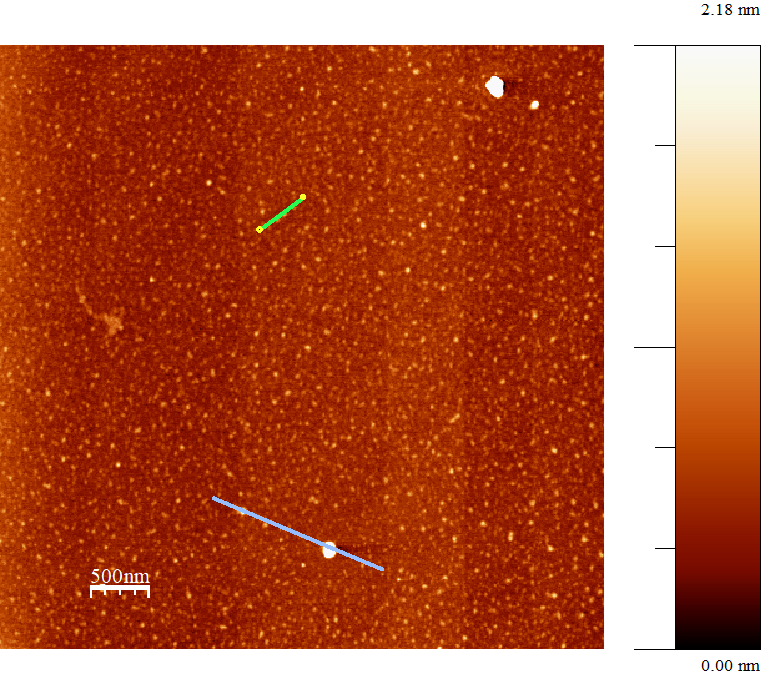** **** |
| --- |

| g  **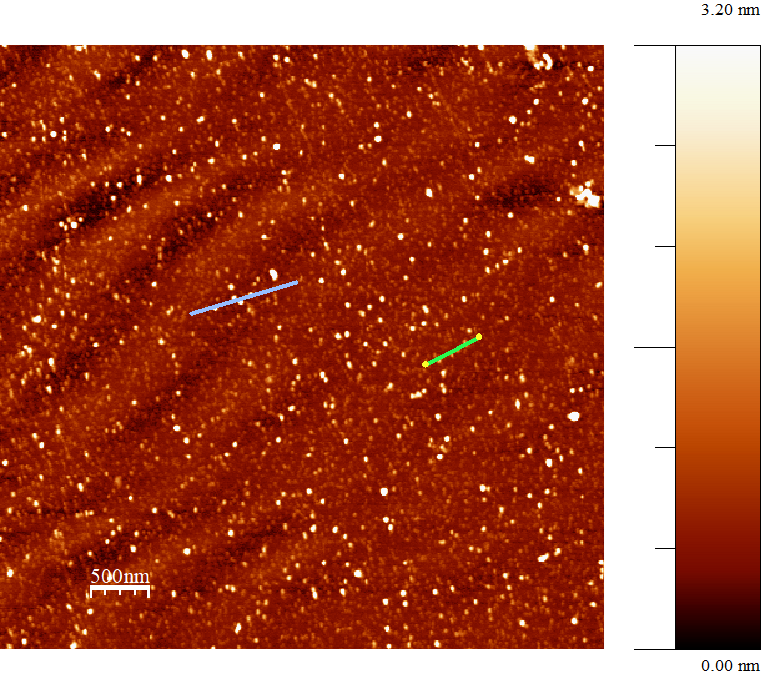** **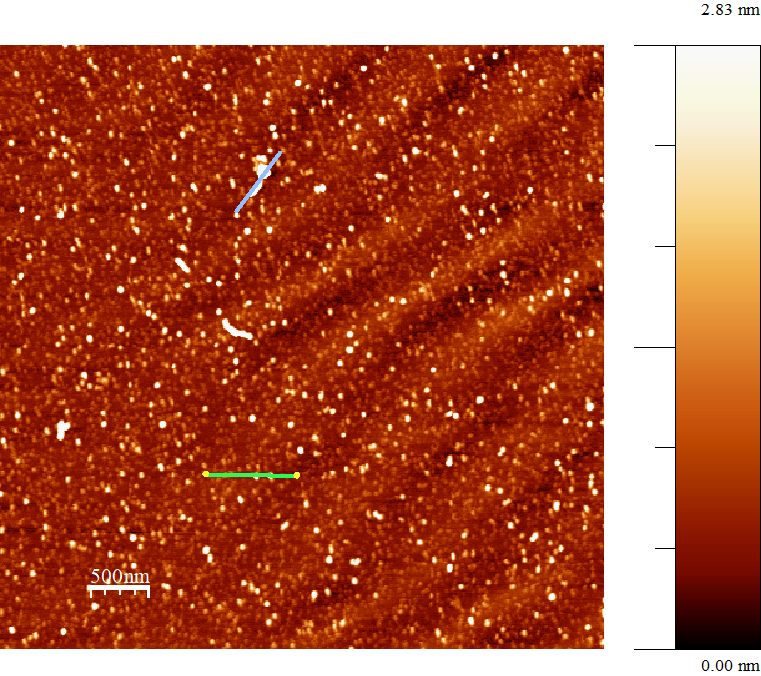** **** |
| --- |

**Figure S4.** AFM images of the CNPs prepared from benzene (CNP 1, a), toluene (CNP 2, b), toluene-NiO (CNP 3, c), chlorobenzene (CNP 4, d), aniline (CNP 5, e), pyrrole (CNP 6, f) and thiophene (CNP 7, g).

| a   | b   |
| --- | --- |
| c   | d   |
| e   | f   |

| g   |  |
| --- | --- |

**Figure S5.** AFM histograms of the CNPs prepared from benzene (CNP 1, a), toluene (CNP 2, b), toluene-NiO (CNP 3, c), chlorobenzene (CNP 4, d), aniline (CNP 5, e), pyrrole (CNP 6, f) and thiophene (CNP 7, g).

**Table S1**. Parameters and statistical data obtained from AFM images.

| Sample | Fitting model | Fit center [nm] | σ [nm] | FWHM [nm] | N total | Mean [nm] | σ [nm] | Minimum [nm] | Median [nm] | Maximum [nm] |
| --- | --- | --- | --- | --- | --- | --- | --- | --- | --- | --- |
| CNP 1 | Lorentz | 2.7 | 1.2 | 2.41 | 720 | 2.8 | 1.3 | 0.98 | 2.92 | 12.1 |
| CNP 2 | Gauss | 1.5 | 0.3 | 0.74 | 4214 | 1.5 | 0.4 | 0.72 | 1.50 | 12.1 |
| CNP 3 | Lorentz | 2.7 | 1.1 | 2.21 | 3194 | 3.6 | 2.1 | 1.64 | 2.96 | 36.88 |
| CNP 4 | Lorentz | 3.6 | 0.7 | 1.48 | 3338 | 3.5 | 0.7 | 1.33 | 3.56 | 5.82 |
| CNP 5 | Gauss | 1.5 | 0.4 | 0.87 | 483 | 2.4 | 2.4 | 0.95 | 1.61 | 33.47 |
| CNP 6 | Gauss | 1.1 | 0.2 | 0.45 | 3147 | 1.3 | 1.3 | 0.73 | 1.16 | 34.75 |
| CNP 7 | Lorentz | 1.9 | 0.4 | 0.86 | 3308 | 2.5 | 1.5 | 1.15 | 2.05 | 39.65 |

**6. Graphs of DLS experiments**

| a 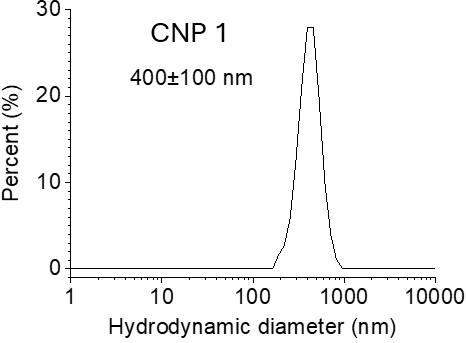 | b 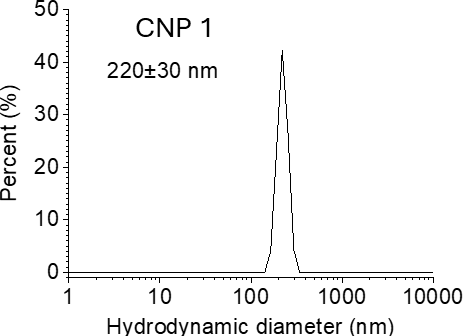 |
| --- | --- |
| c 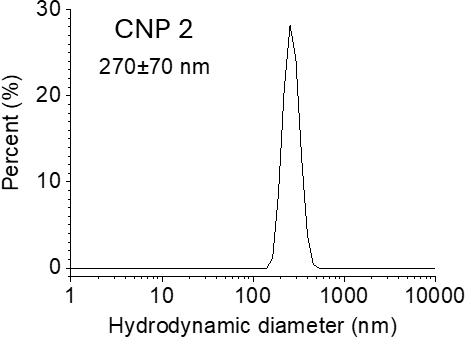 | d 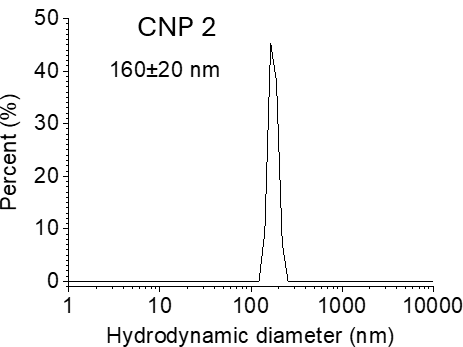 |
| e 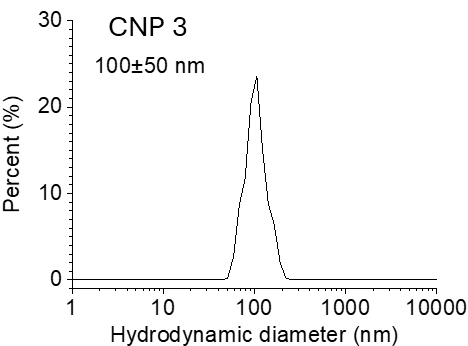 | f 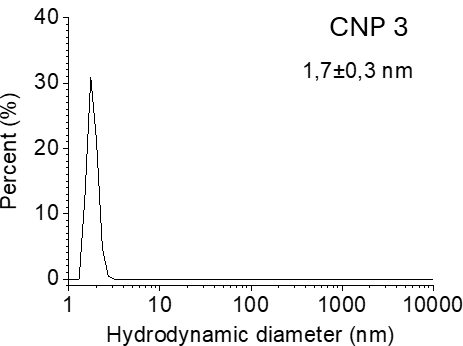 |
| g 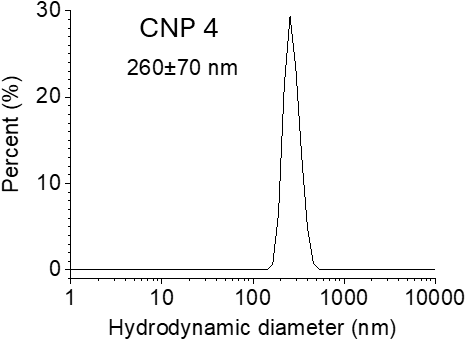 | h 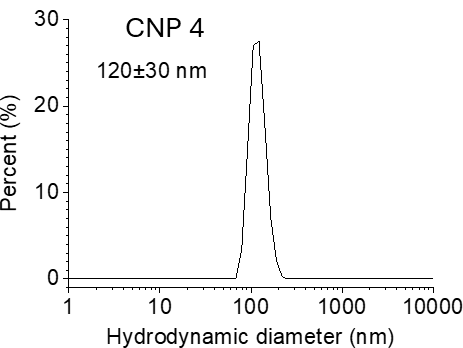 |
| i 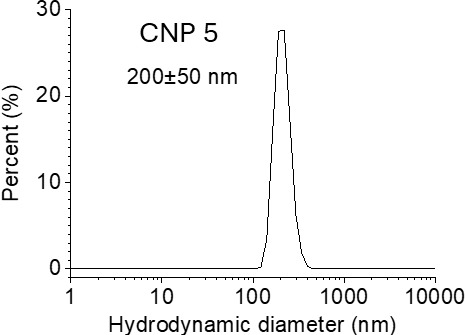 | j 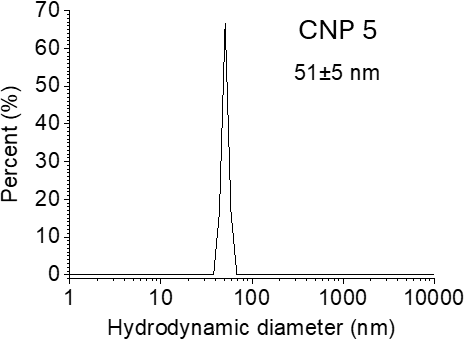 |
| k 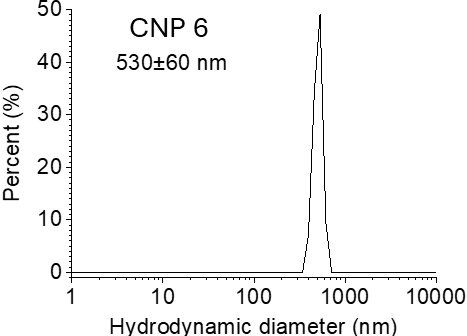 | l 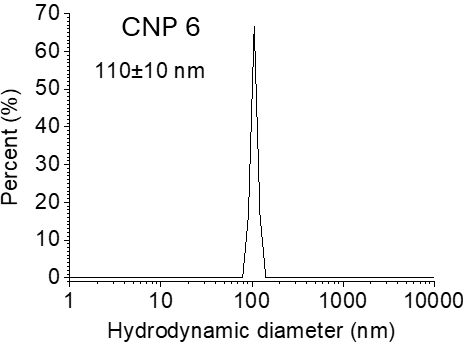 |
| m 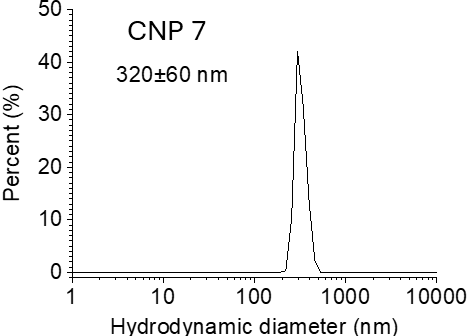 | n 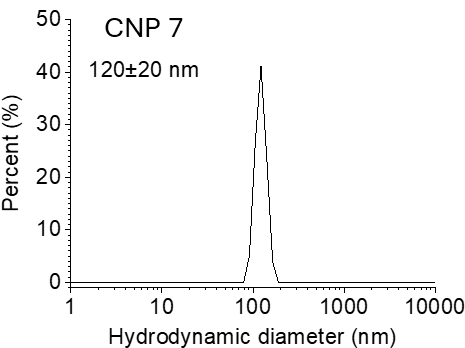 |

**Figure S6.** Graphs of the DLS experiments performed in 2-propanol (left) or toluene (right), respectively, of the CNPs prepared from benzene (CNP 1, a-b), toluene (CNP 2, c-d), toluene-NiO (CNP 3, e-f), chlorobenzene (CNP 4, g-h), aniline (CNP 5, i-j), pyrrole (CNP 6, k-l) and thiophene (CNP 7, m-n).

**7. FTIR spectra**

| a  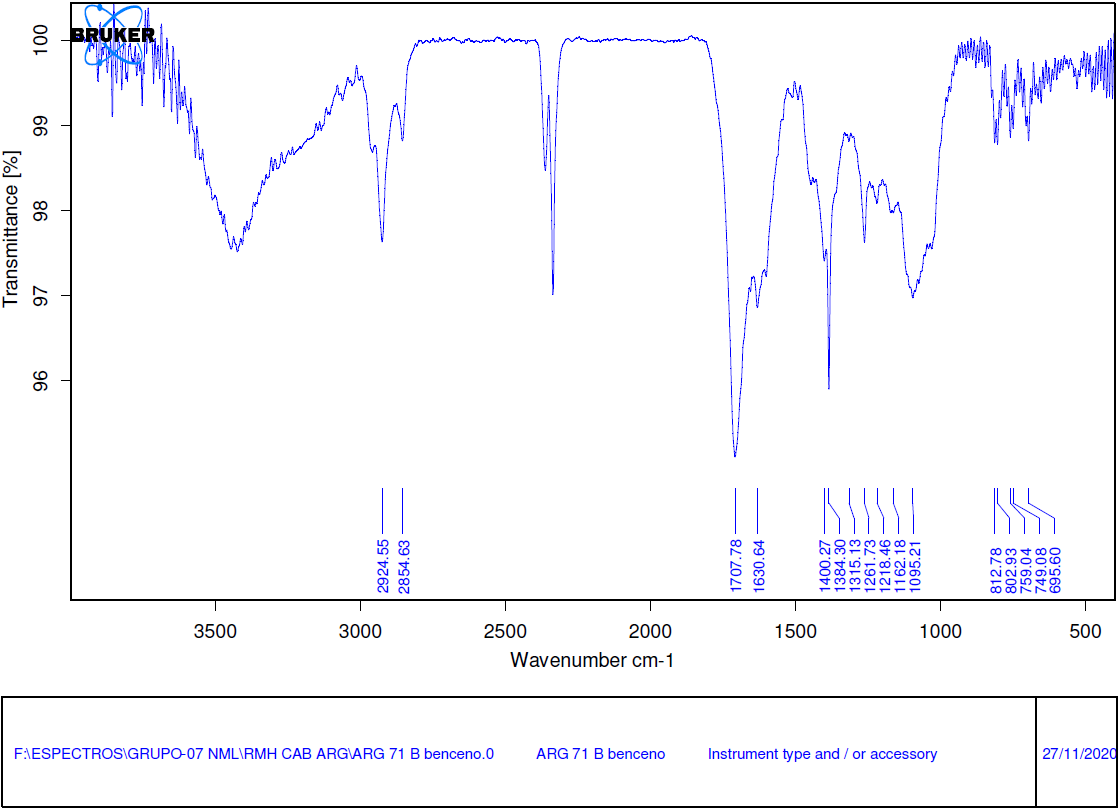 |
| --- |
| b  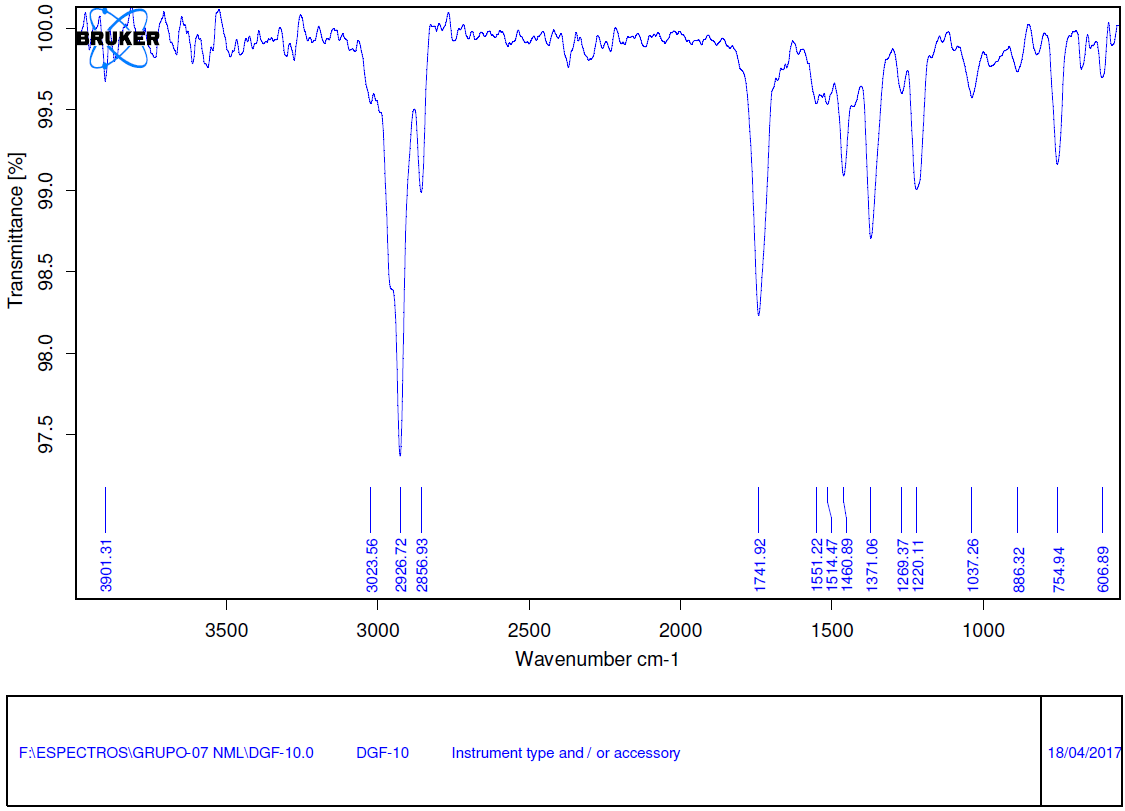 |

| c  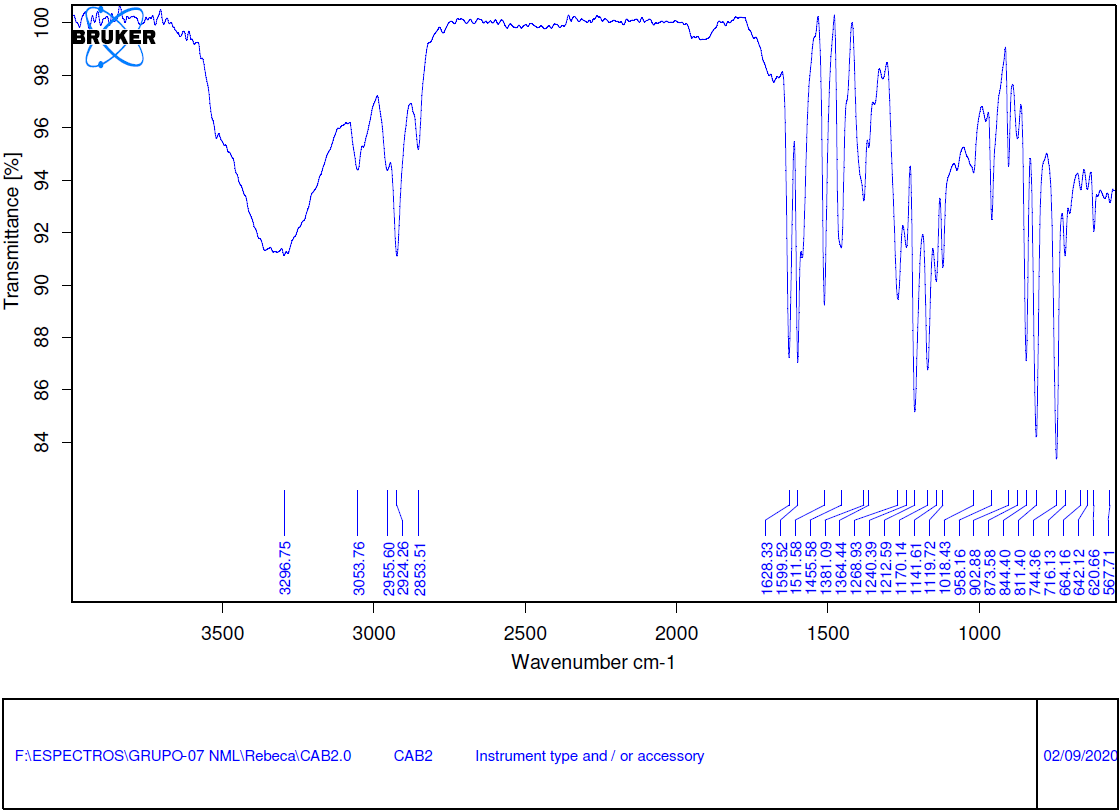 |
| --- |
| d  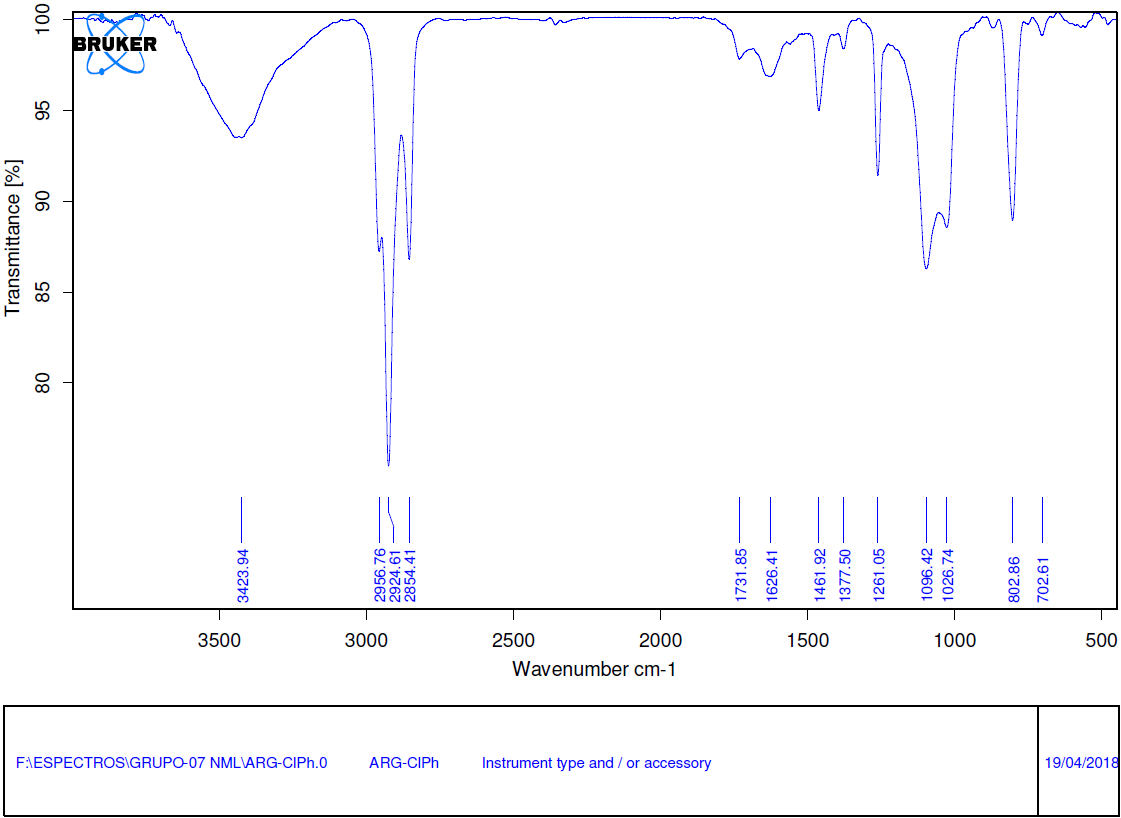 |

| e  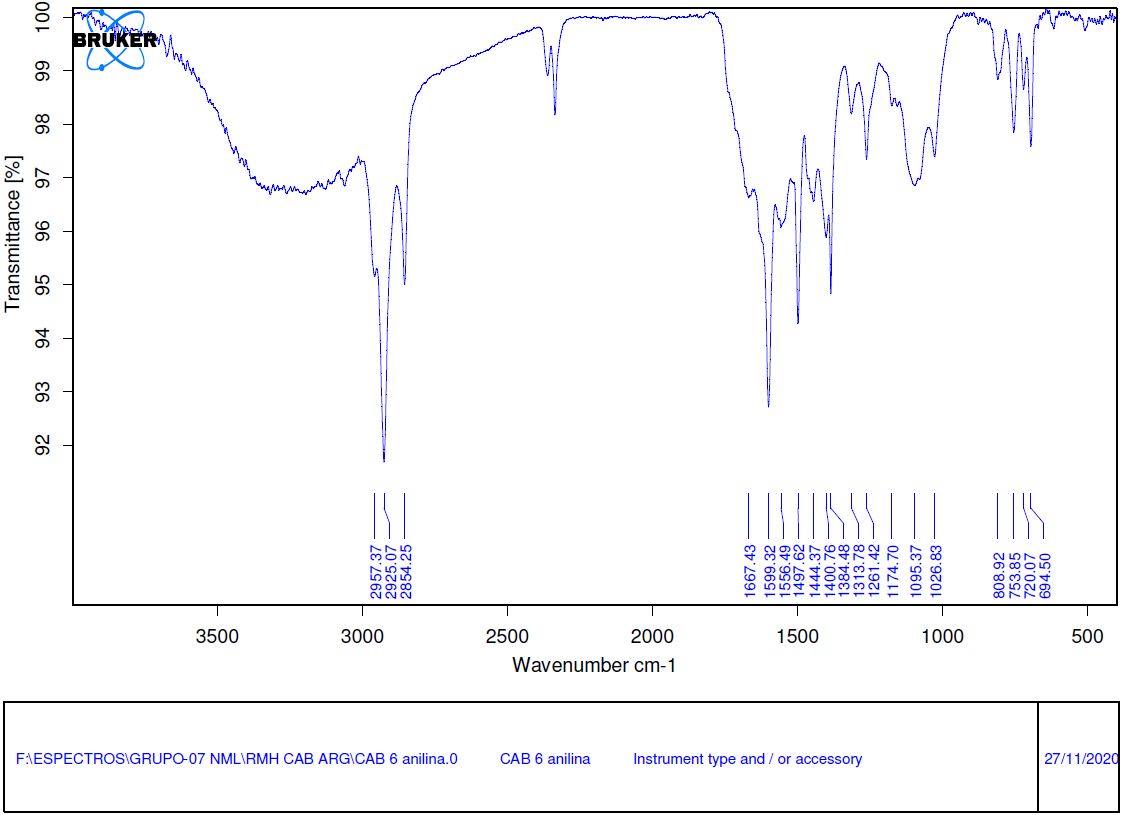 |
| --- |
| f  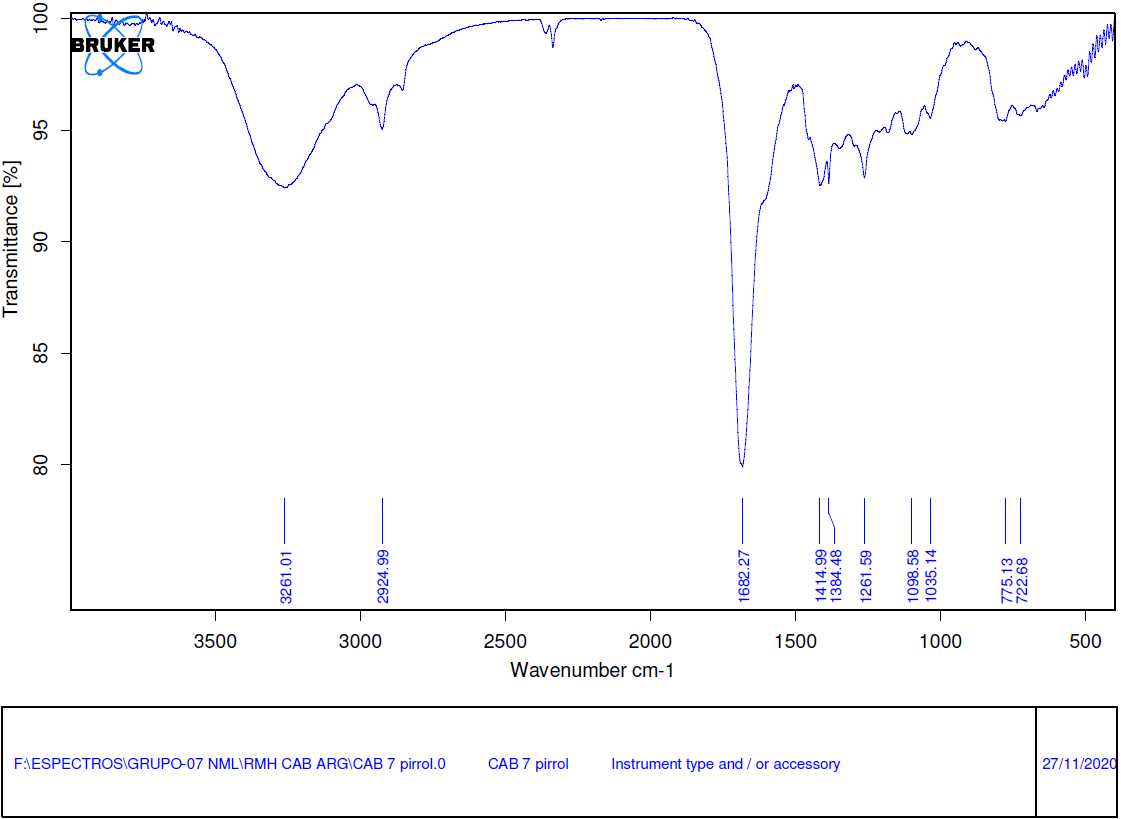 |

| g  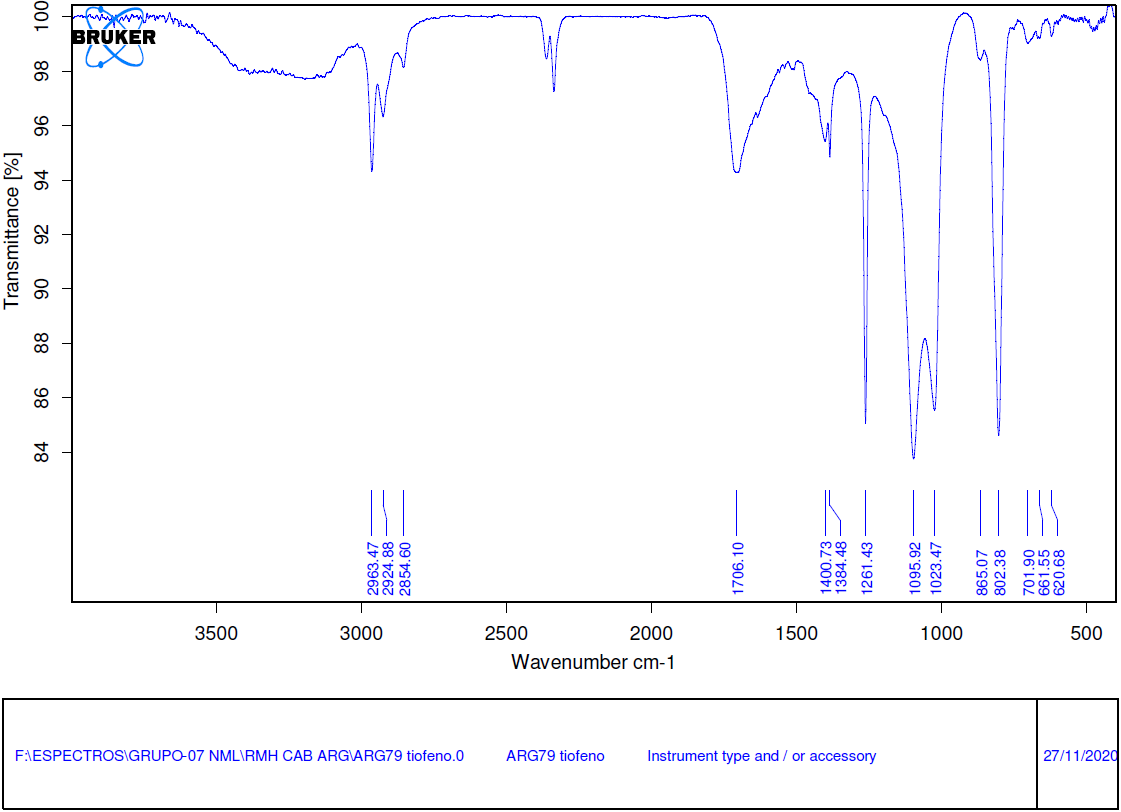 |
| --- |

**Figure S7.** FTIR spectra of the CNPs prepared from benzene (CNP 1, a), toluene (CNP 2, b), toluene-NiO (CNP 3, c), chlorobenzene (CNP 4, d), aniline (CNP 5, e), pyrrole (CNP 6, f) and thiophene (CNP 7, g).

**8. XPS spectra**

| a  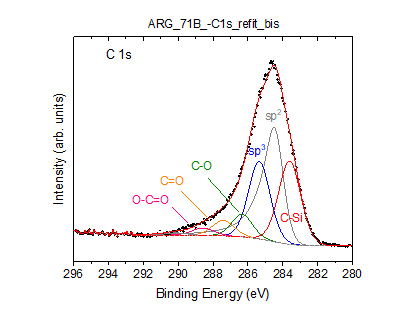 | b  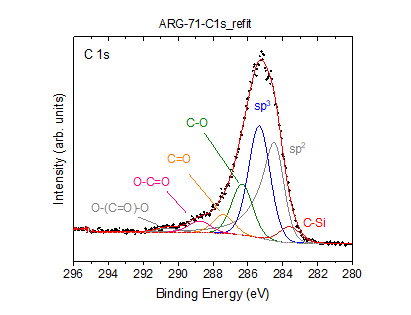 |
| --- | --- |
| c  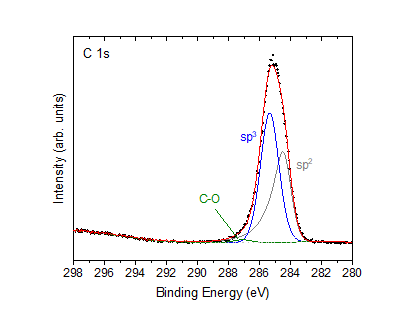 | d  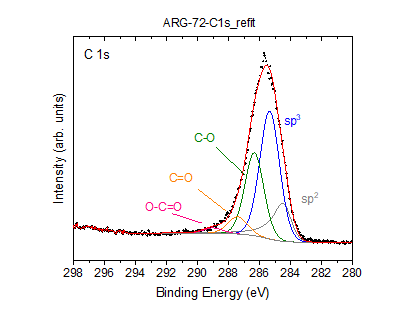 |
| e  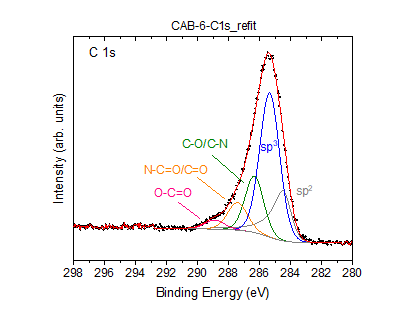 | f  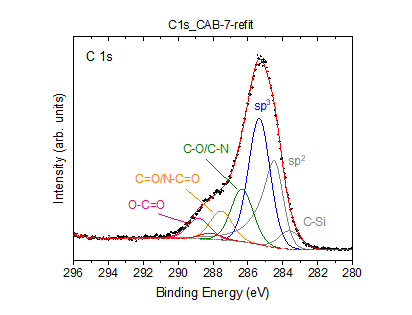 |
| g 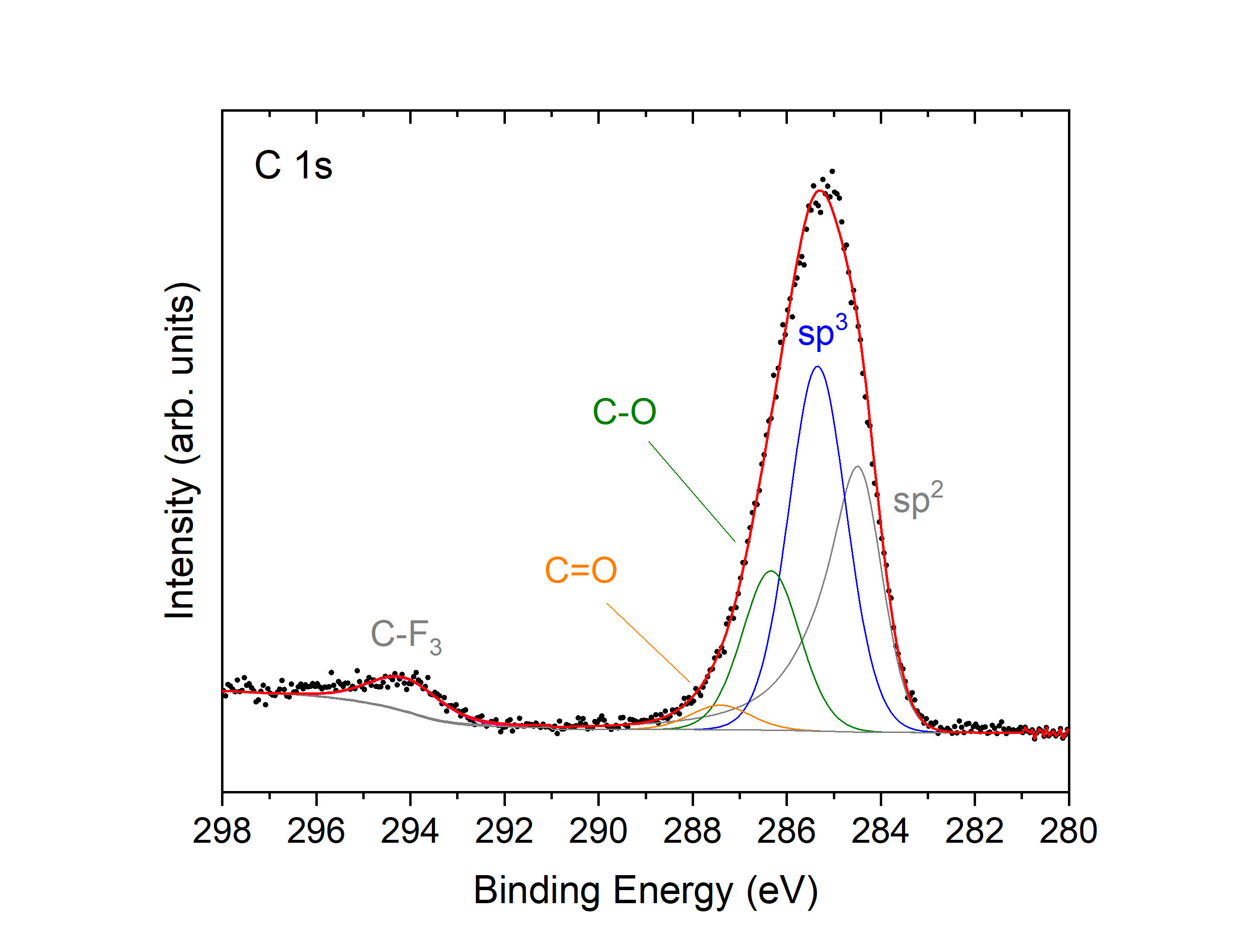 | h  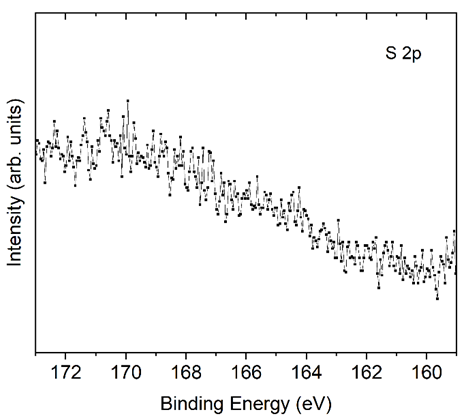 |

**Figure S8.** XPS spectra and C 1s peak fitting of the CNPs prepared from benzene (CNP 1, a), toluene (CNP 2, b), toluene-NiO (CNP 3, c), chlorobenzene (CNP 4, d), aniline (CNP 5, e), pyrrole (CNP 6, f) and thiophene (CNP 7, g). Figure h shows the S 2p peak region of the sample CNP 7, proving the absence of sulfur.

| a   | b   |
| --- | --- |

**Figure S9.** XPS spectra and N 1s peak fitting of the CNPs prepared from aniline (CNP 5, a) and pyrrole (CNP 6, b).

**Table S2**. Atomic % of the main elements detected in each sample.

| Sample | Precursor | % C | % O | % Si | % (other element) |
| --- | --- | --- | --- | --- | --- |
| CNP 1 | Benzene | 72.4 | 23.0 | 4.6 | - |
| CNP 2 | Toluene | 76.0 | 21.9 | 2.1 | - |
| CNP 3 | Toluene-NiO | 96.0 | 4.0 | -^[a]^ | - |
| CNP 4 | Chlorobenzene | 73.8 | 20.0 | 4.1 | 2.2 (Cl) |
| CNP 5 | Aniline | 73.3 | 22.1 | -^[a]^ | 4.6 (N) |
| CNP 6 | Pyrrole | 69.6 | 17.1 | 1.7 | 11.6 (N) |
| CNP 7 | Thiophene | 67.4 | 17.5 | -^[a]^ | 15.1 (F)^[b]^ |

[a] The contribution of Si in this CNP has been removed because it could not be accurately determined due to the use of a Si crystal support with a native SiO_2_ surface. [b] The presence of F is attributed to laser impacts on the Teflon coating of the stirrer bar.

**Table S3.** % Contribution of the main functional groups detected in the XPS analysis of the CNPs studied in the C 1s core level.

| Sample | Precursor | % Csp^2^ | % Csp^3^ | Csp^2^/Csp^2^ | % C-O/C-N | % C=O | % (H/R)O–C=O/N–C=O/ (other) |
| --- | --- | --- | --- | --- | --- | --- | --- |
| CNP 1 | Benzene | 30.9 | 25.4 | 1.22 | 7.1 | 5.2 | 3.3 ((H/R)O–C=O)/28.1 (C-Si) |
| CNP 2 | Toluene | 29.4 | 37.4 | 0.79 | 16.6 | 6.0 | 4.0 ((H/R)O–C=O)/1.4 (O–CO_2_)/5.2 (C-Si) |
| CNP 3 | Toluene-NiO | 36.9 | 61.6 | 0.60 | 1.5 | 0 | - |
| CNP 4 | Chlorobenzene | 11.8 | 49.0 | 0.24 | 30.5 | 6.6 | 2.1 |
| CNP 5 | Aniline | 14.8 | 52.6 | 0.28 | 19.2 | 10.1 | 3.3 |
| CNP 6 | Pyrrole | 22.0 | 38.6 | 0.57 | 16.4 | 9.1 | 6.4((H/R)O–C=O)/1.9 (N-C=O)/5.6 (C-Si) |
| CNP 7 | Thiophene | 27.0 | 45.5 | 0.59 | 20.0 | 3.2 | -/4.3 (C-F)^[b]^ |

[a] This sample contained 4.3 % of F due to contamination by the Teflon coating of the magnet, caused by the impacts of the laser beam on the stirrer.

**9. TGA plots**

| a  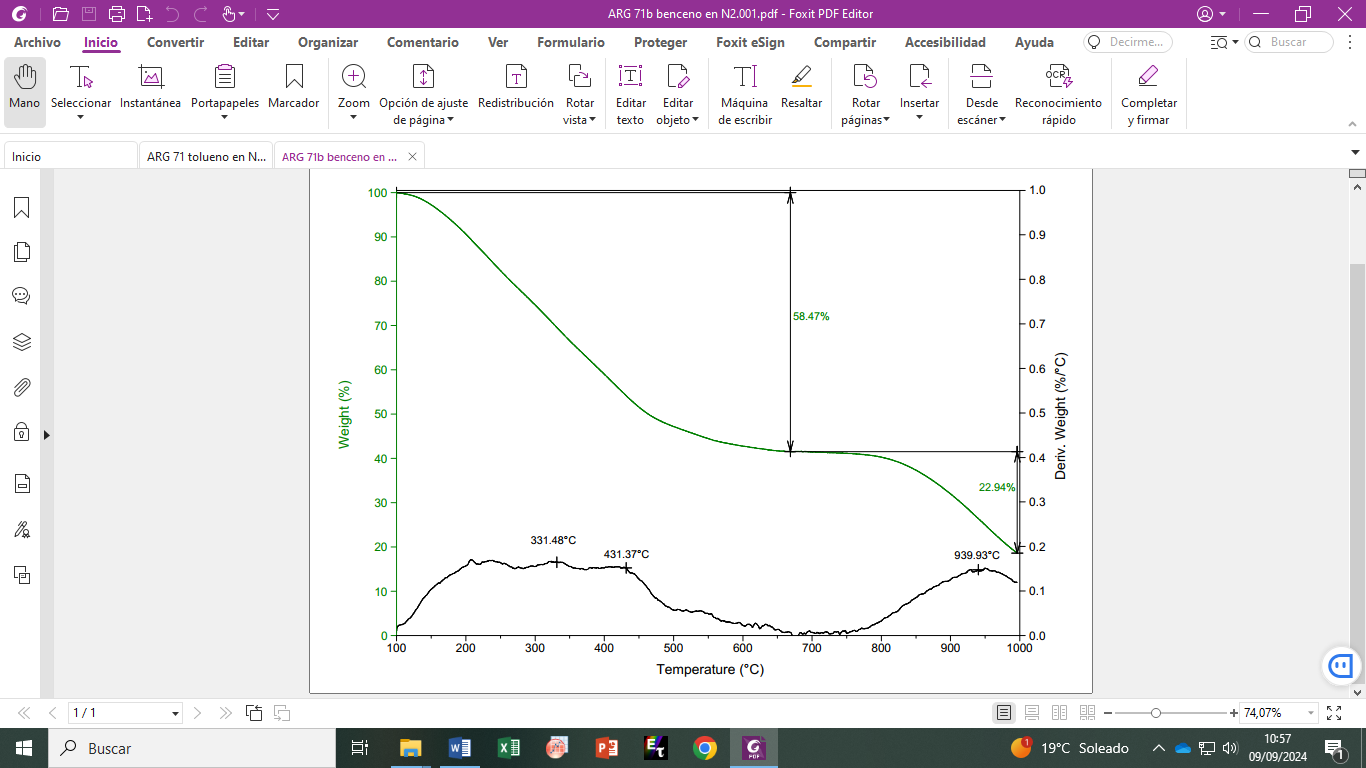 |
| --- |
| b  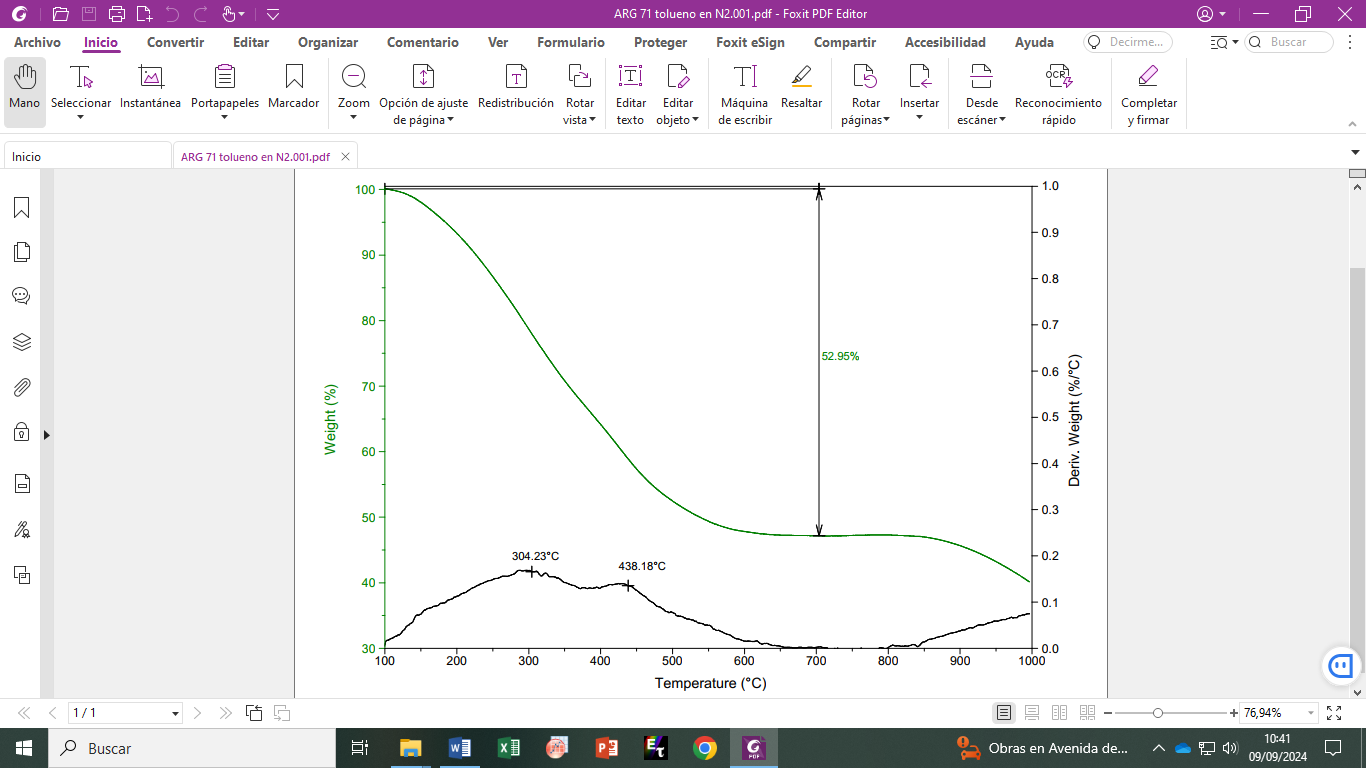 |

| c  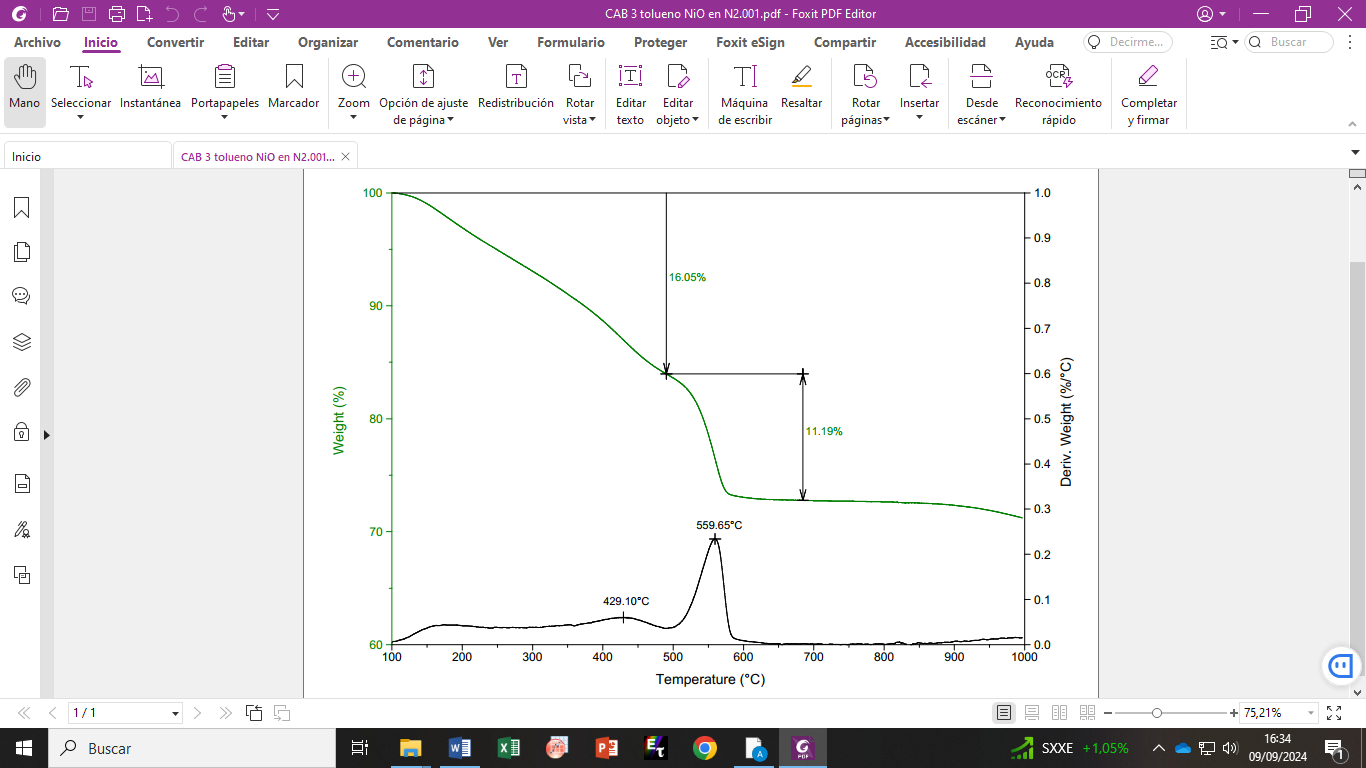 |
| --- |
| d  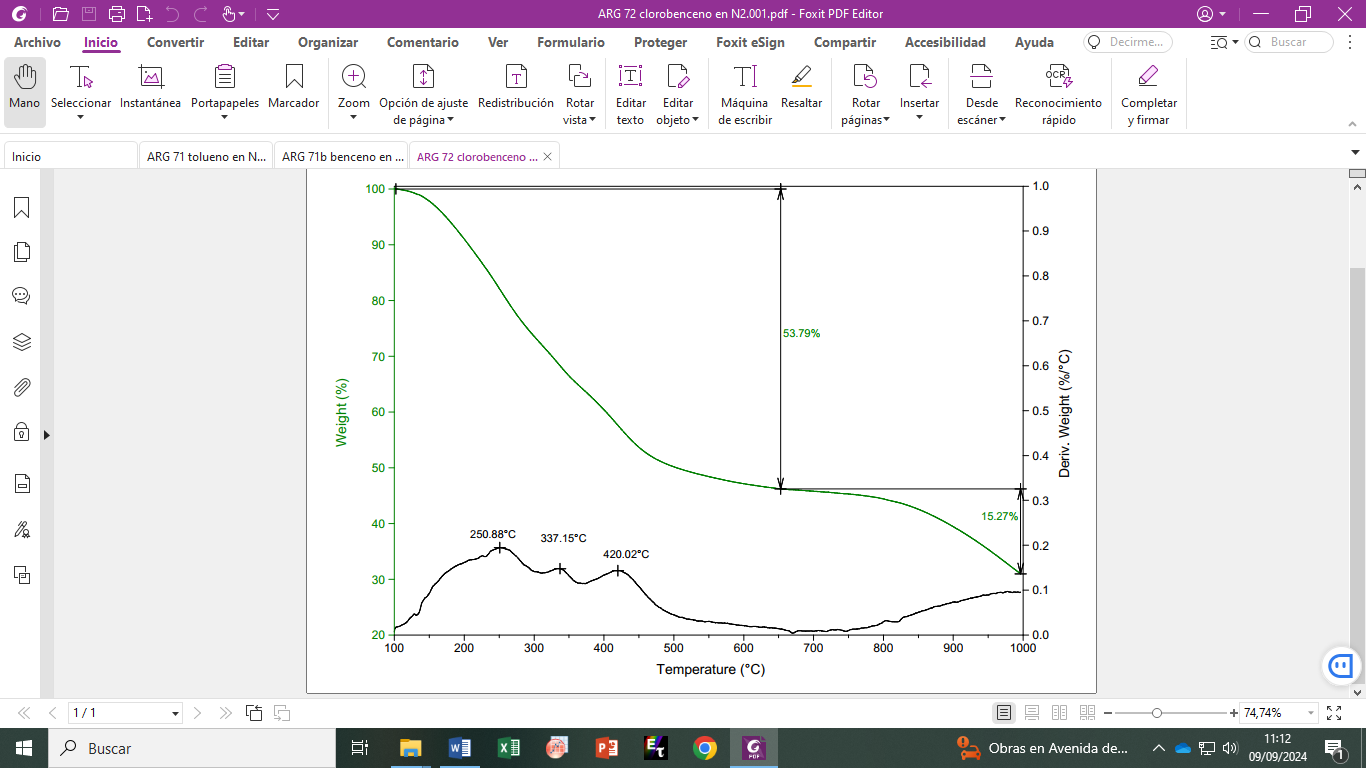 |

| e  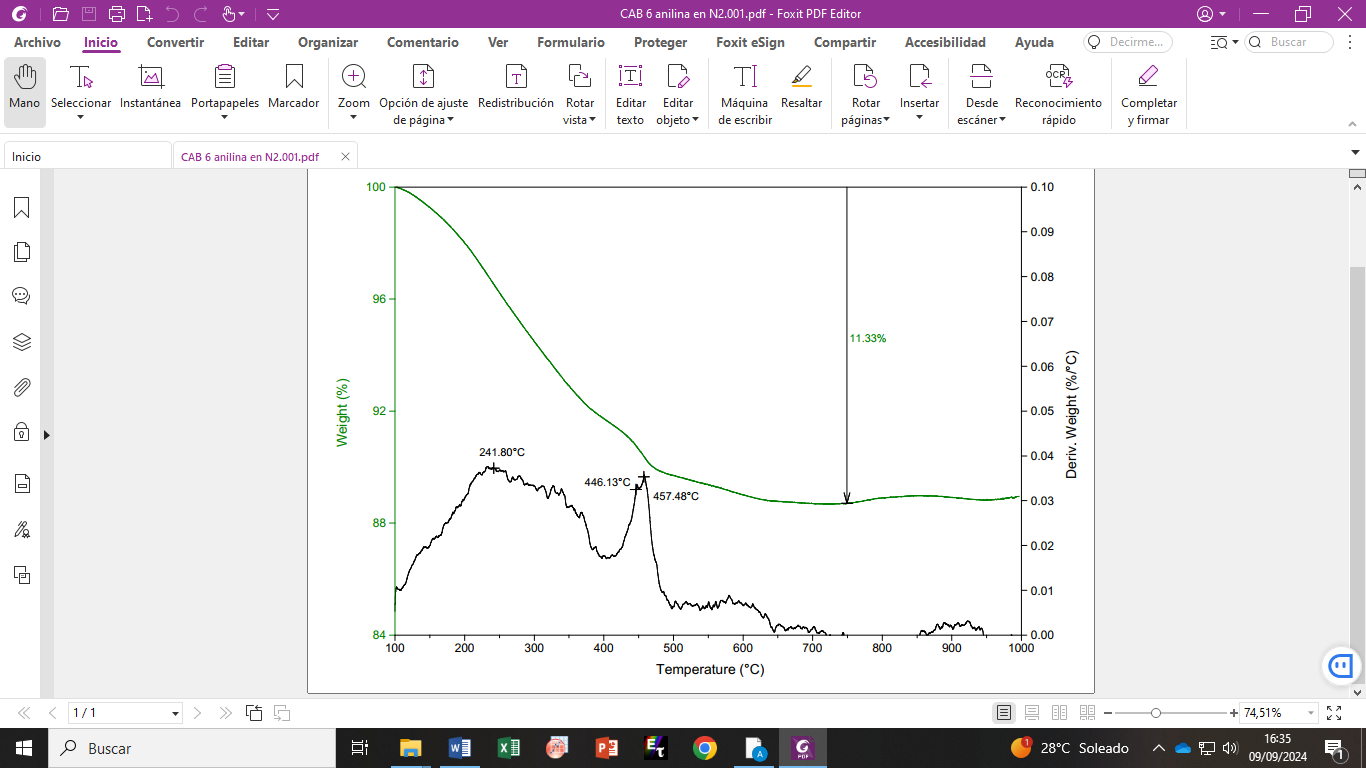 |
| --- |
| f  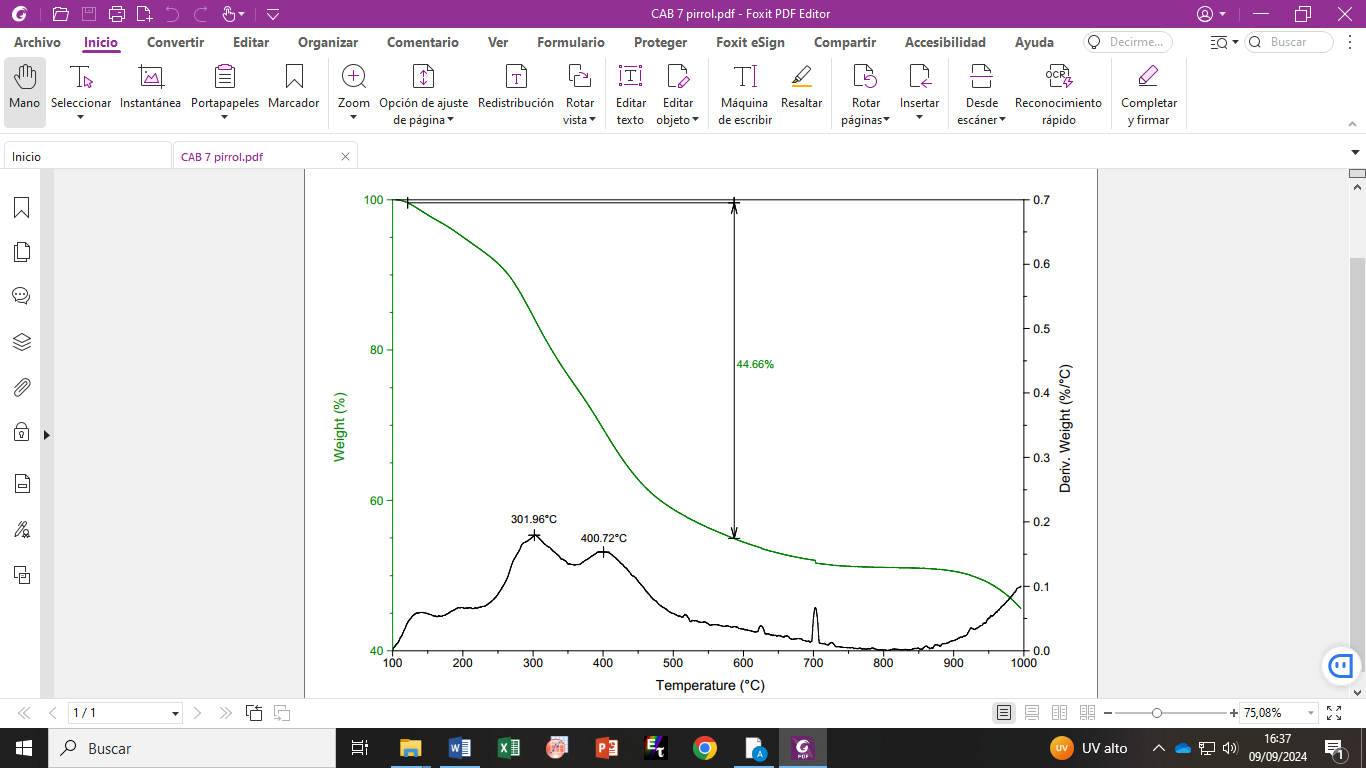 |

| g  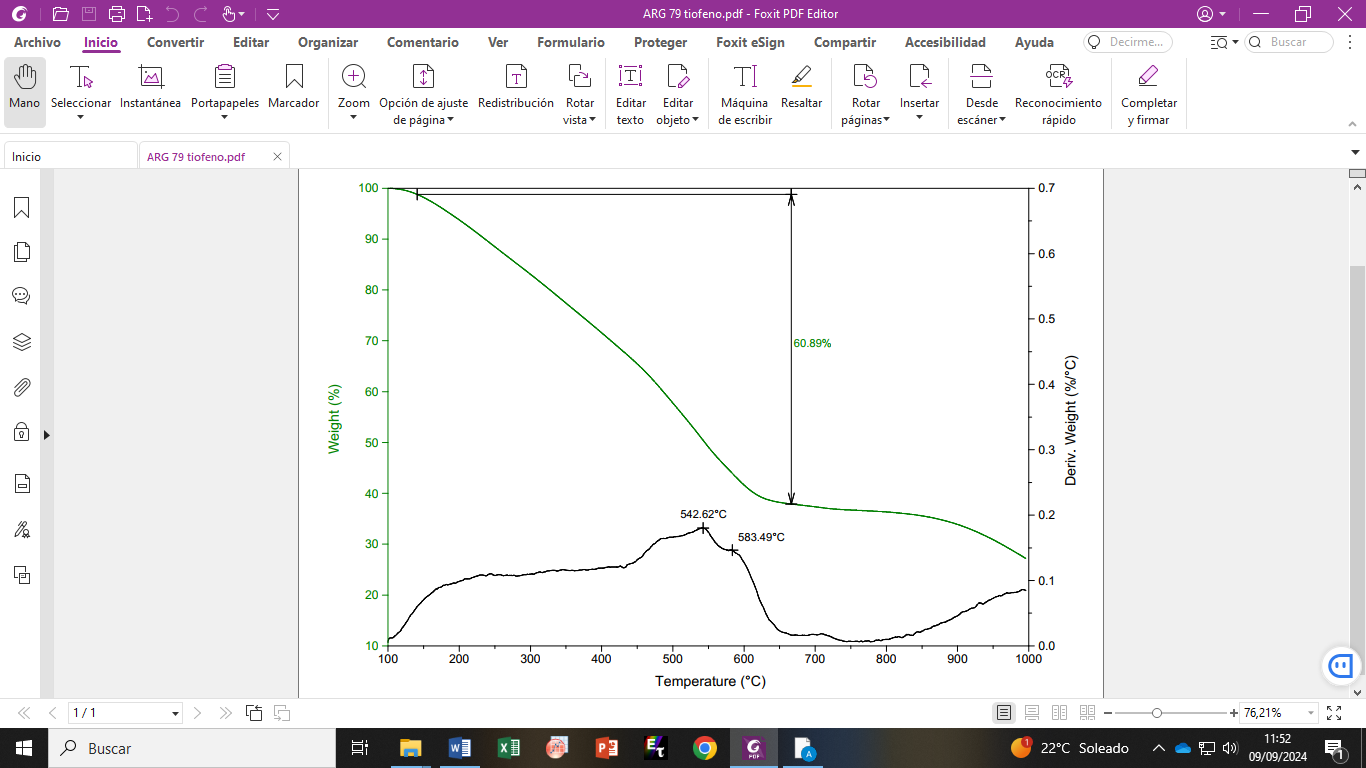 |
| --- |

**Figure S10.** TGA plots of the CNPs prepared from benzene (CNP 1, a), toluene (CNP 2, b), toluene-NiO (CNP 3, c), chlorobenzene (CNP 4, d), aniline (CNP 5, e), pyrrole (CNP 6, f) and thiophene (CNP 7, g).

**10. Kinetics of carbon nanoparticles formation**


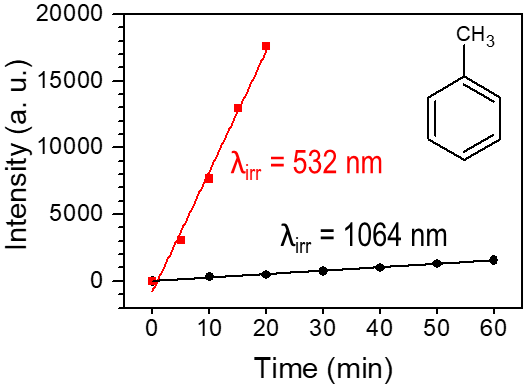


**Figure S11.** Fluorescence intensity vs time plots (*λ*_exc_ = 443 nm, *λ*_det_ = 510 nm, irradiance 2.3 W cm^–2^) of the CNPs prepared from toluene (CNP 2) in a quartz cell.

**Figure S12.** Fluorescence intensity vs time plots (*λ*_exc_ = 443 nm, *λ*_det_ = 510 nm) of the CNPs prepared from toluene (CNP 2) irradiated with 532 nm light in a quartz cell at different flux densities: 3.5 W cm^–2^ (red line) and 2.3 W cm^–2^ (black line).


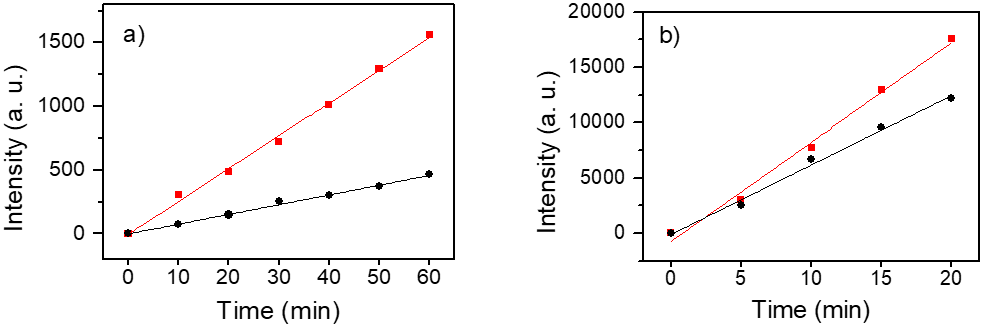


**Figure S13.** Fluorescence intensity vs time plots (*λ*_exc_ = 443 nm, *λ*_det_ = 510 nm) of the CNPs prepared from toluene (CNP 2) in a 1 × 1 cm air-equilibrated fluorescence quartz cell with irradiation at 1064 nm (a) or 532 nm (b) and 2.3 W cm^–2^ flux density. The black lines represent experimental points acquired during continuous excitation at 443 nm with 510 nm monitoring wavelength, while the red lines represent experimental points acquired every 5 min, without continuous irradiation at 443 nm.


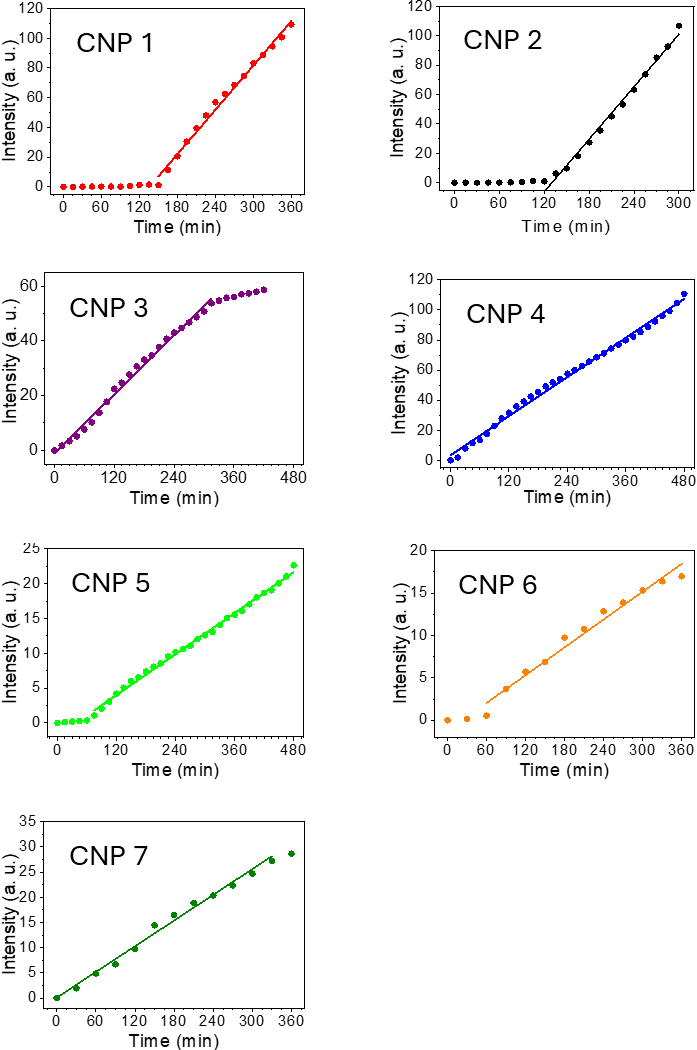


**Figure S14.** Fluorescence intensity vs time plots (*λ*_exc_ = 400 nm, *λ*_det_ = 510 nm for CNP 1–5, and *λ*_exc_ = 320 nm, *λ*_det_ = 385 nm for CNP 6 and 7) monitorizing CNPs formation in the photochemical reactor. Aliquots of 300 μL were drawn from the reactor and diluted to a total volume of 3 mL with 2-propanol prior to acquisition of the emission spectra.

**11. UV-vis. absorption spectra**

**Figure S15.** UV-vis. absorption spectra of the CNPs in 2-propanol corrected for Rayleigh scattering. CNP 1 from benzene (2.90 μg/mL), CNP 2 from toluene (3.33 μg/mL), CNP 3 from toluene-NiO (0.42 μg/mL), CNP 4 from chlorobenzene (63.3 μg/mL), CNP 5 from aniline (8.89 μg/mL), CNP 6 from pyrrole (43.3 μg/mL), CNP 7 from thiophene (66.7 μg/mL).

**Table S4.** Wavelengths of the UV-vis. absorption peaks/(shoulders) of the CNPs in 2-propanol.

| Sample | *λ*_abs_^max^ [nm]^[a]^ | | | | |
| --- | --- | --- | --- | --- | --- |
| CNP 1 | 227 | 271 | - | - | - |
| CNP 2 | 226 | 271 | - | - | - |
| CNP 3 | 226 | (263), 274, (284) | - | 332 | - |
| CNP 4 | 247 | - | 306 | 337 | - |
| CNP 5 | 229 | 276 | - | - | 386 |
| CNP 6 | 222 | 271 | - | 328 | - |
| CNP 7 | 227 | 273, (284) | - | 334 | - |

[a] Experimental uncertainty ± 2 nm.

**12. UV-vis. excitation spectra**

| a  | b  |
| --- | --- |
| c  | d  |
| e  | f  |
| g  |  |

**Figure S16.** UV-vis. excitation spectra of the CNPs prepared from benzene (CNP 1, a), toluene (CNP 2, b), toluene-NiO (CNP 3, c), chlorobenzene (CNP 4, d), aniline (CNP 5, e), pyrrole (CNP 6, f) and thiophene (CNP 7, g) in 2-propanol (25 ± 0.2 ºC). Emission detection wavelengths are 360 nm (gray), 380 nm (red), 400 nm (blue) and 450 nm (green). Insets show the normalized excitation spectra.

**13. UV-vis. emission spectra**

| a   | b  |
| --- | --- |
| c   | d  |
| e   | f  |
| g  |  |

**Figure S17.** UV-vis. emission spectra of the CNPs prepared from benzene (CNP 1, a), toluene (CNP 2, b), toluene-NiO (CNP 3, c), chlorobenzene (CNP 4, d), aniline (CNP 5, e), pyrrole (CNP 6, f) and thiophene (CNP 7, g) in 2-propanol (25 ± 0.2 ºC, optical density <0.1 at the excitation wavelength). Excitation wavelengths are 280 nm (gray), 300 nm (red), 320 nm (blue), 350 nm (green) and 400 nm (purple). Insets show the normalized emission spectra.

**Table S5.** Wavelengths of the UV-vis. emission peaks/shoulders of the CNPs in 2-propanol at different excitation wavelengths.

| Sample | *λ*_em_^max^ [nm]^[a]^ | | | | |
| --- | --- | --- | --- | --- | --- |
|  | *λ*_exc_ 280 nm | *λ*_exc_ 300 nm | *λ*_exc_ 320 nm | *λ*_exc_ 350 nm | *λ*_exc_ 400 nm |
| CNP 1 | 361 | 369 | 361 | 411 | 455 |
| CNP 2 | 365 | 368 | 376 | 411 | 453 |
| CNP 3 | 386 | 381 | 384 | 432 | 457 |
| CNP 4 | 356 | 357 | 369 | 390 | 453 |
| CNP 5 | 409 | 407 | 427 | 438 | 477 |
| CNP 6 | 385 | 391 | 392 | 406 | 478 |
| CNP 7 | 357 | 357 | 354 | 402 | 426 |

[a] 25 ± 0.2 ºC. Experimental uncertainty ± 2 nm. [b] Optical density <0.1 at the excitation wavelength.

**14. Emission lifetimes, deactivation rate constants and Stern-Volmer plots**

**Table S6.** Fluorescence emission decay lifetimes of the CNPs in air-equilibrated 2-propanol, determined at different excitation wavelengths.^[a]^

| Sample | *λ*_exc_ [nm] | *τ*_1_ [ns] | *τ*_2_ [ns] | *τ*_3_ [ns] | *τ*_INT_ [ns] | *τ*_AMP_ [ns] |
| --- | --- | --- | --- | --- | --- | --- |
| CNP 1^[b]^ | 290 | 16.2 | 4.1 | 1.0 | 7.5 | 2.4 |
|  | 375 | 15.2 | 3.9 | 0.9 | 7.2 | 2.6 |
|  | 405 | 12.6 | 4.2 | 1.2 | 6.5 | 3.3 |
|  | 457 | 9.5 | 3.6 | 1.2 | 5.4 | 3.4 |
|  | 502 | - | 4.5 | 1.2 | 3.8 | 2.8 |
| CNP 2^[c]^ | 290 | 15.8 | 4.3 | 1.0 | 6.3 | 2.4 |
|  | 375 | 14.4 | 3.8 | 0.9 | 6.2 | 2.4 |
|  | 405 | 12.5 | 4.3 | 1.3 | 6.1 | 3.2 |
|  | 457 | 10.0 | 3.9 | 1.6 | 4.3 | 2.6 |
|  | 502 | - | 3.5 | 1.3 | 2.2 | 1.8 |
| CNP 3^[d]^ | 290 | 15.0 | 4.2 | 1.2 | 5.7 | 2.9 |
|  | 375 | 9.4 | 2.8 | 0.7 | 3.4 | 2.4 |
|  | 405 | 9.6 | 2.9 | 0.9 | 3.6 | 2.0 |
|  | 457 | 7.9 | 2.9 | 0.9 | 4.0 | 2.3 |
|  | 502 | 8.0 | - | 1.3 | 4.3 | 2.0 |
| CNP 4^[e]^ | 290 | 15.9 | 4.2 | 0.9 | 5.0 | 1.7 |
|  | 375 | 10.3 | 2.5 | 0.6 | 4.0 | 1.5 |
|  | 405 | 10.0 | 2.9 | 0.9 | 3.8 | 1.8 |
|  | 457 | 9.0 | 3.1 | 1.0 | 3.7 | 2.1 |
|  | 502 | - | 3.8 | 0.8 | 2.9 | 1.8 |
| CNP 5^[f]^ | 290 | 15.5 | 4.3 | 1.2 | 6.3 | 3.4 |
|  | 375 | 12.2 | 3.3 | 0.8 | 4.3 | 2.0 |
|  | 405 | 10.3 | 3.2 | 1.1 | 3.9 | 2.3 |
|  | 457 | 8.5 | 3.3 | 1.2 | 3.6 | 2.4 |
|  | 502 | - | 4.3 | 1.1 | 3.3 | 2.2 |
| CNP 6^[g]^ | 405 | 12.3 | 4.2 | 1.0 | 4.9 | 3.3 |
| CNP 7^[h]^ | 405 | 11.2 | 4.6 | 1.0 | 4.1 | 2.3 |

[a] Values determined in air-equilibrated solvent, 25 ± 0.2 ºC. [b] Experimental error 8–13%. [c] Experimental error 9–22%. [d] Experimental error 10–26%. [e] Experimental error 10–21%. [f] Experimental error 14–16%. [g] Experimental error 10–19%. [h] Experimental error 9–18%.

**Table S7.** Fluorescence emission decay lifetimes of the CNPs in 2-propanol under Ar-purged, air-equilibrated and O_2_-purged conditions, determined at excitation wavelengths of 375, 405 or 457 nm.^[a]^

| Sample^[b]^ | % O_2_ | *τ*_1_ [ns] | *τ*_2_ [ns] | *τ*_3_ [ns] | *τ*_INT_ [ns] | *τ*_AMP_ [ns] |
| --- | --- | --- | --- | --- | --- | --- |
| CNP 1^[c]^ | 0 | *16.8* | *3.8* | *0.84* | *7.9* | *2.2* |
|  | 21 | 15.2 | 3.9 | 0.91 | 7.2 | 2.6 |
|  | 100 | 13.8 | 3.6 | 0.90 | 5.4 | 2.2 |
| CNP 2^[d]^ | 0 | *14.9* | *3.7* | *0.88* | *6.8* | *2.1* |
|  | 21 | 14.4 | 3.8 | 0.91 | 6.2 | 2.4 |
|  | 100 | 13.7 | 3.6 | 0.89 | 4.8 | 1.9 |
| CNP 3^[e]^ | 0 | *10.0* | *3.1* | *0.94* | *3.8* | *1.6* |
|  | 21 | 9.4 | 2.8 | 0.66 | 3.4 | 2.4 |
|  | 100 | 8.7 | 2.7 | 0.95 | 3.2 | 1.6 |
| CNP 4^[f]^ | 0 | *10.7* | *2.5* | *0.50* | *4.7* | *1.0* |
|  | 21 | 10.3 | 2.5 | 0.61 | 4.2 | 1.4 |
|  | 100 | 8.9 | 2.8 | 0.70 | 3.9 | 2.4 |
| CNP 5^[g]^ | 0 | *12.7* | *3.1* | *0.93* | *4.8* | *2.1* |
|  | 21 | 12.2 | 3.3 | 0.84 | 4.3 | 2.0 |
|  | 100 | 11.7 | 3.1 | 0.96 | 3.9 | 1.9 |
| Sample^[h]^ | % O_2_ | *τ*_1_ [ns] | *τ*_2_ [ns] | *τ*_3_ [ns] | *τ*_INT_ [ns] | *τ*_AMP_ [ns] |
| CNP 6^[i]^ | 0 | *12.8* | *4.3* | *1.1* | *5.4* | *3.5* |
|  | 21 | 12.3 | 4.2 | 0.99 | 4.9 | 3.3 |
|  | 100 | 11.6 | 4.2 | 1.0 | 4.0 | 3.1 |
| CNP 7^[j]^ | 0 | *11.6* | *4.7* | *1.1* | *4.5* | *2.6* |
|  | 21 | 11.2 | 4.6 | 1.0 | 4.1 | 2.3 |
|  | 100 | 10.6 | 4.5 | 1.0 | 3.3 | 2.2 |
| Sample^[k]^ | % O_2_ | *τ*_1_ [ns] | *τ*_2_ [ns] | *τ*_3_ [ns] | *τ*_INT_ [ns] | *τ*_AMP_ [ns] |
| CNP 1^[c]^ | 0 | *10.6* | *3.4* | *0.95* | *5.7* | *2.9* |
|  | 21 | 9.5 | 3.6 | 1.2 | 5.4 | 3.4 |
|  | 100 | 9.0 | 3.5 | 0.96 | 3.7 | 2.0 |
| CNP 2^[d]^ | 0 | *11.3* | *3.9* | *1.1* | *5.6* | *3.0* |
|  | 21 | 10.0 | 3.9 | 1.6 | 4.3 | 2.6 |
|  | 100 | 9.6 | 3.9 | 0.9 | 3.4 | 1.8 |
| CNP 3^[e]^ | 0 | *8.3* | *3.0* | *0.8* | *4.2* | *1.9* |
|  | 21 | 7.9 | 2.9 | 0.9 | 4.0 | 2.3 |
|  | 100 | 7.2 | 2.6 | 0.7 | 2.9 | 1.5 |
| CNP 4^[f]^ | 0 | *9.4* | *3.2* | *0.8* | *4.0* | *2.7* |
|  | 21 | 9.0 | 3.1 | 1.0 | 3.8 | 2.7 |
|  | 100 | 8.0 | 3.0 | 0.9 | 3.5 | 2.5 |
| CNP 5^[g]^ | 0 | *9.1* | *3.3* | *0.82* | *4.0* | *2.0* |
|  | 21 | 8.5 | 3.3 | 1.2 | 3.6 | 2.4 |
|  | 100 | 7.7 | 3.0 | 0.82 | 3.0 | 1.7 |

[a] Values determined under Ar-purged conditions are displayed in italics. [b] *λ*_exc_ = 375 nm. [c] Experimental error 8–13%. [d] Experimental error 9–22%. [e] Experimental error 10–26%. [f] Experimental error 10–21%. [g] Experimental error 14–16%. [h] *λ*_exc_ = 405 nm. [i] Experimental error 10–19%. [j] Experimental error 8–15%. [k] *λ*_exc_ = 457 nm.

| a  | b  |
| --- | --- |
| c  |  |

**Figure S18.** Stern-Volmer plots of singlet exciton quenching of CNPs 1–7 by molecular oxygen in 2-propanol at 25 ± 2 ºC, with *λ*_exc_ 375 nm (a, CNPs 1–5), 457 nm (b, CNPs 1–5) or 405 nm (c, CNPs 6 and 7). Graphs of steady-state measurements are shown in filled symbols, while open symbols correspond to time-resolved measurements. CNP 1 (■, □), CNP 2 (●, ○), CNP 3 (▲, △), CNP 4 (⯁, ◇), CNP 5 (★, ☆), CNP 6 (⬢, ⬡), CNP 7 (▶, ▷).

**Table S8.** Intensity-weighted average fluorescence lifetimes, and bimolecular deactivation rate constants for fluorescence quenching by molecular oxygen of the CNPs in 2-propanol, determined at excitation wavelengths of 375, 405 or 457 nm.^[a]^

| Sample^[b]^ | *τ*_0INT_ [ns] | *k*_q_^S^_O2_*_τ_*_INT_ [M^–1^s^–1^]^[e]^ | *k*_q_^S^_O2_*_I_*_em_ [M^–1^s^–1^]^[e]^ |
| --- | --- | --- | --- |
| CNP 1 | 7.9 | 5.7 10^9^ (± 1%) | 8.1 10^9^ (± 5%) |
| CNP 2 | 6.8 | 5.9 10^9^ (± 2%) | 8.4 10^9^ (± 9%) |
| CNP 3 | 3.8 | 4.5 10^9^ (± 23%) | 5.8 10^9^ (± 7%) |
| CNP 4 | 4.7 | 4.1 10^9^ (± 23%) | 4.7 10^9^ (± 7%) |
| CNP 5 | 4.8 | 5.3 10^9^ (± 23%) | 5.6 10^9^ (± 7%) |
| Sample^[c]^ | *τ*_0INT_ [ns] | *k*_q_^S^_O2_*_τ_*_INT_ [M^–1^s^–1^] | *k*_q_^S^_O2_*_I_*_em_ [M^–1^s^–1^] |
| CNP 6 | 5.4 | 6.3 10^9^ (± 10%) | 8.0 10^9^ (± 10%) |
| CNP 7 | 4.5 | 7.8 10^9^ (± 10%) | 6.0 10^9^ (± 9%) |
| Sample^[d]^ | *τ*_0INT_ [ns] | *k*_q_^S^_O2_*_τ_*_INT_ [M^–1^s^–1^] | *k*_q_^S^_O2_*_I_*_em_ [M^–1^s^–1^] |
| CNP 1 | 5.7 | 9.6 10^9^ (± 11%) | 5.9 10^9^ (± 7%) |
| CNP 2 | 5.6 | 1.0 10^10^ (± 25%) | 4.9 10^9^ (± 9%) |
| CNP 3 | 4.2 | 1.1 10^10^ (± 10%) | 5.0 10^9^ (± 15%) |
| CNP 4 | 4.0 | 3.3 10^9^ (± 17%) | 4.2 10^9^ (± 12%) |
| CNP 5 | 4.0 | 1.4 10^10^ (± 11%) | 3.8 10^9^ (± 1%) |

[a] Absorbance ≤0.1 at *λ*_exc_. [b] *λ*_exc_ 375 nm. [c] *λ*_exc_ 405 nm. [d] *λ*_exc_ 457 nm. [e] Relative error of the slopes obtained by linear regression in parentheses.

| a  | b  |
| --- | --- |
| c  | d  |
| e  |  |

**Figure S19.** Stern-Volmer plots of triplet exciton quenching of CNPs 1–5 by molecular oxygen in 2-propanol-glycerol mixture (1:1, v/v) at 25 ± 2 ºC with *λ*_exc_ 355 nm. Oxygen solubility values as in neat 2-propanol have been assumed since the bimolecular quenching rate constants obtained from O_2_ solubility values in glycerol are above the diffusion-controlled limit.

**Table S9.** Summary of singlet exciton deactivation rate constants and quantum yields of the CNPs 1–5 in 2-propanol, determined from data with excitation at 355 nm.^[a],[b]^

| Sample | *τ*_0AMP_ [ns] | *Φ*_em_ | *k_r_* [s^–1^] | *k_nr_* [s^–1^] | *P*_O2_^T^ | *Φ*_Δ_ | *Φ*_T_^[c]^ | *k*_isc_ [s^–1^]^[d]^ | *k*_ic_ [s^–1^]^[e]^ | *Φ*_ic_^[f]^ |
| --- | --- | --- | --- | --- | --- | --- | --- | --- | --- | --- |
| CNP 1 | 2.3 | 0.09 | 3.9 10^7^ | 4.0 10^8^ | 0.83 | 0.37 | 0.45 | 1.9 10^8^ | 2.0 10^8^ | 0.46 |
| CNP 2 | 2.1 | 0.09 | 4.3 10^7^ | 4.3 10^8^ | 0.96 | 0.35 | 0.36 | 1.7 10^8^ | 2.6 10^8^ | 0.55 |
| CNP 3 | 1.9 | 0.05 | 2.6 10^7^ | 5.0 10^8^ | 0.97 | 0.28 | 0.29 | 1.5 10^8^ | 3.5 10^8^ | 0.66 |
| CNP 4 | 1.6 | 0.03 | 1.9 10^7^ | 6.1 10^8^ | 0.94 | 0.09 | 0.10 | 6.0 10^7^ | 5.5 10^8^ | 0.87 |
| CNP 5 | 2.0 | 0.03 | 1.5 10^7^ | 4.9 10^8^ | 0.95 | 0.24 | 0.25 | 1.3 10^8^ | 3.6 10^8^ | 0.72 |

[a] Data of *τ*_0AMP_ and *Φ*_em_, *k*_r_ and *k*_nr_ have been taken from **Table 2**. [b] Data of *P*_O2_^T^ and *Φ*_Δ_ have been taken from **Table 3**. [c] *Φ*_T_ = *Φ*_Δ_ / *P*_O2_^T^. [d] *k*_isc_ = *Φ*_T_ / *τ*_0AMP_. [e] *k*_ic_ = (1 / *τ*_0AMP_) – *k*_r_ – *k*_isc_. [f] *Φ*_ic_ = *k*_ic_ × *τ*_0AMP_.

**References**

[46] W. L. F. Armarego, C. L. L. Chai, Purification of Laboratory Chemicals, 5th Ed., Butterworth-Heinemann – Elsevier, Burlington (MA), **2003**.

[47] a) C. Niedermeier, M. Råsander, S. Rhode, V. Kachkanov, B. Zou, N. Alford, M. A. Moram. Band gap bowing in NiₓMg₁₋ₓO, *Sci. Rep*. **2016**, *6*, 31230; b) M. El-Kemary, N. Nagy, I. El-Mehasseb. Nickel oxide nanoparticles: Synthesis and spectral studies of interactions with glucose *Mater. Sci. Semicond. Process*. **2013**, *16*, 1747.

[48] a) D. Galvan, Y. T. Pei, J. Th. M. De Hosson, A. Cavaleiro. Deformation and failure mechanism of nano-composite coatings under nano-indentation. *Surf. Coat. Technol*. **2005**, *200*, 739; b) O. O. Mykhaylyk, Y. M. Solonin, D. N. Batchelder, R. Brydson. Transformation of nanodiamond into carbon onions: A comparative study by high-resolution transmission electron microscopy, electron energy-loss spectroscopy, x-ray diffraction, small-angle x-ray scattering, and ultraviolet Raman spectroscopy. *J. Appl. Phys.* **2005**, *97*, 074302; c) J. Bruley, D. B. Williams, J. J. Cuomot, D. P. Pappas. Quantitative near-edge structure analysis of diamond-like carbon in the electron microscope using a two-window method. *J. Microscopy* **1995**, *180*, 22; d) S. D. Berger, D. R. McKenzie, P. J. Martin. EELS analysis of vacuum arc-deposited diamond-like films. *Philos. Mag. Lett*. **1988**, *57*, 285.

[49] I. Horcas, R. Fernández, J. M. Gómez-Rodríguez, J. Colchero, J. Gómez-Herrero, A. M. Baró. A software for scanning probe microscopy and a tool for nanotechnology. *Rev. Sci. Instrum.* **2007**, *78*, 013705.

[50] D. F. Eaton. Reference materials for fluorescence measurement, *Pure Appl. Chem*. **1988**, *7*, 1107.

[51] C. Buck, B. Gramlich, S. Wagner. Production and properties of the liquid scintillators used in the Stereo reactor neutrino experiment, *J. Instrum.* **2015**, *10*, P09007.

[52] J. Koziol. Spectral characteristics of riboflavin, riboflavin tetrabutyrate and lumichrome. *Photochem. Photobiol.***1966**, *5*, 41.

[53] M. Montalti, A. Credi, L. Prodi, M. T. Gandolfi, Handbook of Photochemistry, 3rd ed., CRC, Boca Raton, FL, 2006, chap. 9, Table 9a, pp. 536–540.

[54] A. Jiménez-Banzo. X. Ragàs. P. Kapusta, S. Nonell. Time-resolved methods in biophysics. Photon counting vs. analog time-resolved singlet oxygen phosphorescence detection. *Photochem. Photobiol. Sci.* **2008**, *7*, 1003.

[55] R. Schmidt, C. Tanielian, R. Dunsbach, C. Wolff, Phenalenone, a universal reference compound for the determination of quantum yields of singlet oxygen O₂(¹Δg) sensitization. *J. Photochem. Photobiol. A* **1994**, *79*, 11.

[56] M. Montalti, A. Credi, L. Prodi, M. T. Gandolfi, Handbook of Photochemistry, 3rd ed., CRC, Boca Raton, FL, 2006, chap. 9, Table 9c, pp. 542–548.
